# Tractometric: A Diffusion Tractography Software Engine for Integration into a Neuronavigation System: Technical Development and Preliminary Clinical Feasibility

**DOI:** 10.3390/brainsci16101123

**Published:** 2026-10-17

**Authors:** William Mendez, Alejandra Bohórquez, Ximena Bohórquez, Oscar Bohórquez, William J. Shelton, Edgar G. Ordóñez-Rubiano

**Affiliations:** 1 Biomedical Engineering, Navimetric Latinoamerica S.A.S., Bogotá 111211, Colombia; wifemendez28@gmail.com (W.M.); ingenieria@navimetriclatinoamerica.com (A.B.);; 2 Neurosurgery Research Department, Navimetric Latinoamerica S.A.S., Bogotá 111211, Colombia; 3 School of Medicine, Universidad de Los Andes, Bogotá 111711, Colombia; wj.sheltonc@gmail.com; 4 Department of Neurosurgery, Hospital Universitario Fundación Santa Fe de Bogotá, Bogotá 110111, Colombia; 5 Department of Neurosurgery, Fundación Universitaria de Ciencias de La Salud–Universidad FUCS, Hospital de San José–Sociedad de Cirugía de Bogotá, Bogotá 110221, Colombia

**Keywords:** neuronavigation system, diffusion tensor imaging, tractography, low-to-middle-income countries

## Abstract

**Highlights:**

**What are the main findings?**

- Tractometric is a diffusion tractography engine that integrates with an existing neuronavigation platform, supporting a complete workflow from diffusion data acquisition to surgical planning.
- In 8 patients, reconstructed motor and language pathways were concordant with intraoperative stimulation findings.

**What are the implications of the main findings?**

- Integrated tractography software may offer a viable option for institutions unable to acquire full commercial navigation platforms, particularly in low-resource settings.
- Compatibility with existing neuronavigation infrastructure supports incremental adoption of advanced imaging tools without requiring system replacement.

**Abstract:**

Background: Diffusion MRI tractography can support patient-specific neurosurgical planning, but implementation may be limited by workflow complexity, software requirements, and resource constraints. We describe Tractometric^®^, a diffusion tractography software engine developed for integration with the Surgimetric^®^ neuronavigation planning environment. Methods: Tractometric^®^ was developed through a multidisciplinary, iterative co-design process involving neurosurgeons and neuroimaging specialists. The software uses a modular Model–View–Controller architecture implemented in Python (version 3.11) and supports multiple diffusion reconstruction models, deterministic and probabilistic tracking, patient-specific and atlas-derived regions of interest (ROIs), visualization, and interoperable export. The present study was designed as a technical development and preliminary clinical feasibility report. Technical workflow characteristics were described retrospectively across 30 cases, while preliminary clinical feasibility was explored in 8 patients undergoing tractography-assisted surgical planning. Intraoperative direct cortical and subcortical electrical stimulation was used as a qualitative clinical reference rather than as a formal diagnostic-accuracy test. Results: Tractometric^®^ implemented a complete workflow from diffusion data loading and preprocessing to tract reconstruction, filtering, visualization, and export to Surgimetric^®^. The 30-case technical series illustrated variation in processing time and user interaction according to operator experience, reconstruction model, registration, ROI strategy, and manual cleaning. In 8 preliminary clinical cases, reconstructed motor and language pathways were qualitatively concordant with positive intraoperative stimulation findings. No formal sensitivity, specificity, spatial agreement, or test–retest reproducibility estimates were calculated. Conclusions: Tractometric^®^ provides an integrated technical workflow for diffusion tractography and neuronavigation planning and was feasible to implement in a preliminary clinical series. The current data support technical feasibility and workflow integration, but do not establish anatomical accuracy, reproducibility, clinical efficacy, or cost-effectiveness. Independent, prospective validation using standardized acquisitions, predefined tractography parameters, quantitative spatial metrics, and systematic intraoperative mapping is warranted.

## 1. Introduction

Despite ongoing advances in surgical technology—including artificial intelligence, augmented reality, intraoperative imaging, and diffusion magnetic resonance imaging (dMRI) [1,2]—access to advanced neurosurgical technologies remains uneven across healthcare systems, particularly in low- and middle-income countries (LMICs) [3,4]. Integrated platforms that combine specialized hardware, proprietary software, technical support, and neuronavigation may require substantial institutional investment. In some Latin American settings, including Colombia, advanced technologies may also be provided through case-based rental models, which can reduce the need for capital acquisition but may still generate substantial per-procedure costs [3,5]. These considerations provide an important rationale for developing interoperable software workflows that can operate within existing imaging and navigation infrastructures. However, the economic value of any individual software platform requires a formal comparison of acquisition, implementation, maintenance, training, and support costs and should not be inferred from software purchase price alone.

Multiple diffusion MRI tractography platforms are available, including DSI Studio, MRtrix3, DIPY, and SlicerDMRI, among others [6,7,8,9]. These tools have expanded access to diffusion reconstruction, but the translation of tractography into clinical workflows remains technically demanding. Differences in preprocessing, diffusion models, seeding strategies, stopping criteria, registration, and streamline filtering can materially influence the resulting tractograms [10,11,12]. Moreover, tractography is intrinsically susceptible to false-positive and false-negative reconstructions, and quantitative validation generally requires reference anatomy, phantoms, tracer data, or other predefined ground-truth approaches [11,12,13]. Accordingly, a software platform intended for neurosurgical use should be evaluated not only by whether it can generate tractograms, but also by the transparency, interoperability, reproducibility, and clinical workflow in which those tractograms are produced.

In this study, we introduce Tractometric^®^ (Bogotá, Colombia), a diffusion tractography software engine designed for integration with the Surgimetric^®^ neuronavigation planning environment. The primary objective was to describe the technical architecture, processing workflow, diffusion reconstruction capabilities, and interoperability of the platform. A secondary objective was to report preliminary clinical feasibility in a small retrospective series in which tractography was incorporated into neurosurgical planning and interpreted alongside intraoperative functional mapping. The study was intentionally structured as a technical development and feasibility report rather than as a formal validation, diagnostic-accuracy, usability, outcomes, or cost-effectiveness study. This distinction is important because the available data do not support quantitative estimates of anatomical accuracy, test–retest reproducibility, clinical efficacy, or economic benefit.

## 2. Methods

### 2.1. System Development

The Tractometric^®^ software (version 1.0.10.3) was developed through a multidisciplinary collaboration between neurosurgeons and neuroimaging specialists. An iterative co-design approach was used to translate clinical requirements into software functions, with repeated feedback concerning user interface and user experience (UI/UX), clinical interpretability of tractographic outputs, workflow sequence, and compatibility with the Surgimetric^®^ surgical planning and navigation environment. Because several members of the development team also participated in subsequent testing or clinical interpretation, the present report emphasizes technical characterization and feasibility rather than independent performance validation.

The system was designed to process dMRI data in standard medical formats (Digital Imaging and Communications in Medicine [DICOM], Neuroimaging Informatics Technology Initiative [NifTI], b-values [BVALs]/b-vectors [BVECs]) and supports the integration of structural MRI as an anatomical reference for the registration and segmentation of regions of interest (ROIs).

The implementation was guided by four software-level attributes: anatomical consistency of the reconstructed output with the processed image space, interoperability with Surgimetric^®^ planning and navigation workflows, interactive performance during computationally intensive operations, and extensibility for additional diffusion models, registration methods, and segmentation approaches. These attributes describe design goals and should not be interpreted as independently validated performance outcomes. The software user manual is provided as Supplementary File S1.

### 2.2. Software Architecture

**Figure 1:**
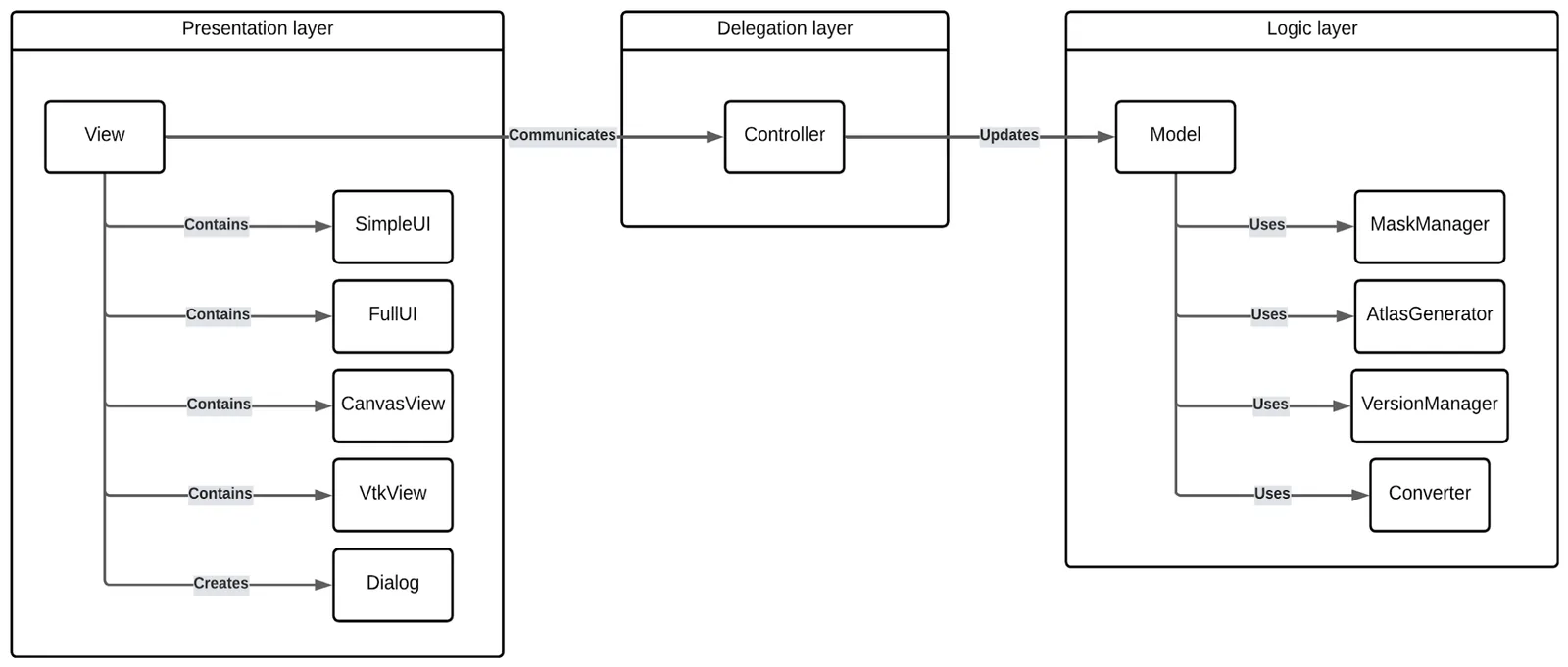
Simplified Unified Modeling Language class diagram of the Tractometric^®^ architecture. The diagram separates the View, Controller, and Model layers and illustrates the main interactions between user-interface components, workflow orchestration, and diffusion-processing modules. The architecture is intended to isolate computational components from presentation logic and to permit future replacement or addition of diffusion models, registration methods, and visualization functions.

Tractometric^®^ was implemented in Python, following a Model–View–Controller (MVC) architecture to ensure modularity, maintainability, and scalability. Each layer was designed with distinct responsibilities to optimize system performance and facilitate future extensions (Figure 1).

#### 2.2.1. View Layer

The View layer manages data presentation and user interactions through a multimodal visualization framework, which includes: 2D visualization of anatomical slices in multiple planes using Matplotlib (version 3.8.4), 3-dimensional (3D) volumetric and tractographic rendering via Visualization Toolkit (VTK) (version 9.3.0), user interface components built with ttkbootstrap (version 1.10.1) and Tkinter (version 8.6.13), providing a modern, modular design, dedicated dialog systems for specialized user interactions, dual-interface design—offering (i) a simplified mode with optimized default parameters for standard workflows, and (ii) an advanced mode allowing granular control of tractography parameters.

#### 2.2.2. Controller Layer

The Controller layer orchestrates communication between user actions and the logic layer, incorporating: (1) Centralized management of execution time for computationally intensive operations, (2) context-aware error handling with immediate user feedback, (3) parallel and asynchronous coordination between the interface and processing modules to maintain real-time responsiveness, and (4) abstraction of multi-step logical processes into single user commands, simplifying workflow complexity.

#### 2.2.3. Model Layer

The Model layer contains the domain-specific logic for diffusion MRI processing. It handles reading, conversion, and storage of DICOM, NIfTI, BVAL, and BVEC data; preprocessing operations such as motion/distortion correction and denoising; diffusion-model reconstruction; orientation-peak estimation; threshold-based mask generation and seed placement; ROI management using either user-defined regions or atlas-derived regions registered to the patient dataset; and tractography using deterministic or probabilistic approaches. The implementation includes DTI [14], SHORE [15,16], GQI [17], CSA [18], and SfM [19] reconstruction frameworks, together with tract filtering, clustering, and visualization-support functions. The layer uses DIPY (version 1.9.0) [6], NiBabel (version 5.2.1), and pydicom (version 3.0.1) for selected data-handling and diffusion-processing operations, while caching and version-control mechanisms support computational efficiency and maintainability.

### 2.3. System Maintainability and Extensibility

The MVC architecture enables independent evolution of each system component. The View layer can be modified or expanded without affecting computational logic, while the Model layer can incorporate new diffusion algorithms, atlas databases, image registration methods, or visualization optimizations without requiring structural changes in other layers. This design ensures long-term adaptability for both research and clinical use.

### 2.4. Implementation Details

The software was developed in Python 3.11, adopting a modular architecture designed to promote component reusability and a clear logical separation of responsibilities. The overall structure follows the MVC paradigm, facilitating code maintenance and enabling the integration of new functionalities without compromising system stability or performance.

### 2.5. Internal Code Organization

**Figure 2:**
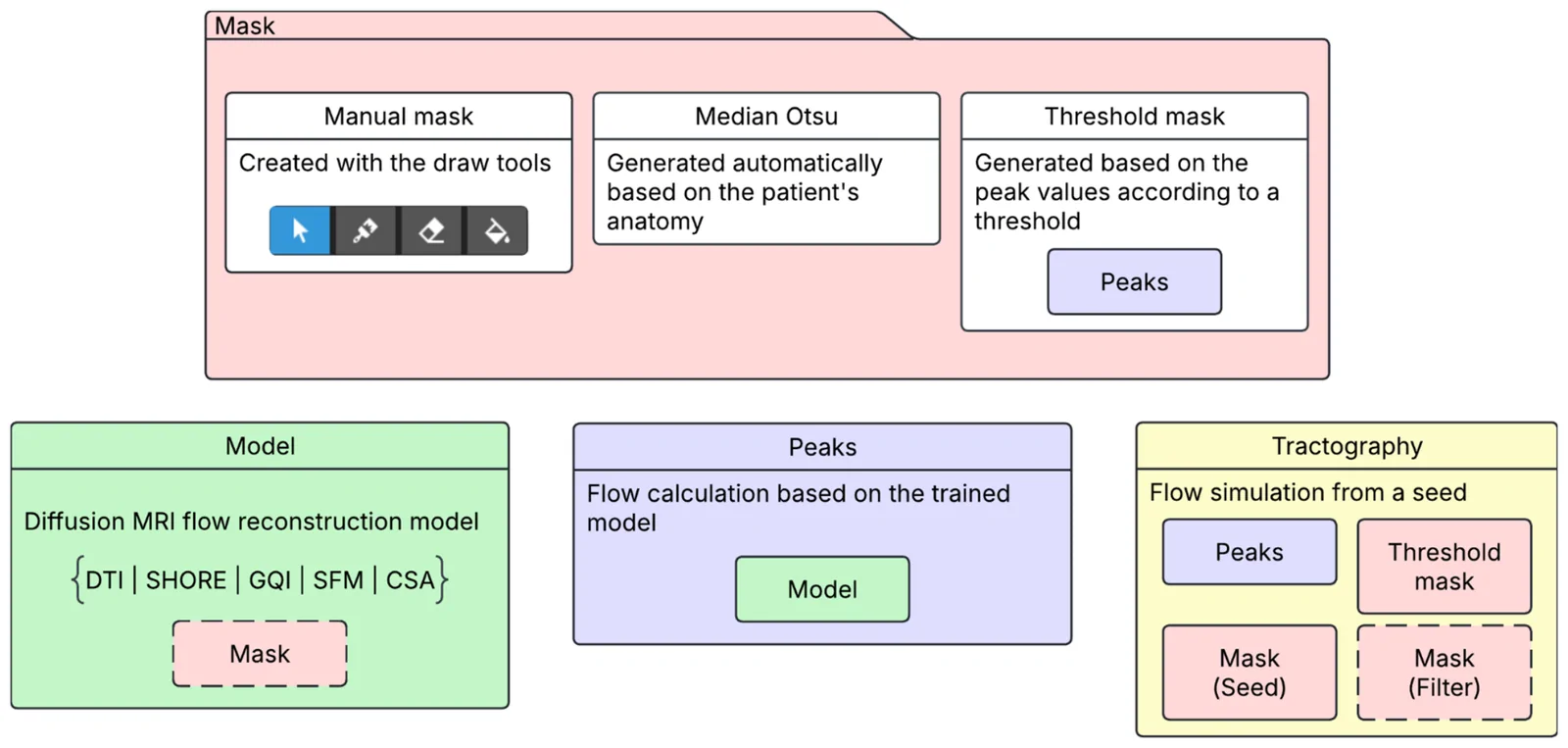
Internal data model and software organization. Directory-level components correspond to classes implementing interchangeable processing functions, including alternative diffusion-reconstruction, orientation-estimation, registration, tractography, and visualization operations. The figure is intended to document modularity and interoperability of the implementation rather than to imply equivalence between the different reconstruction models.

The internal codebase follows a hierarchical organization in which directories correspond to processing-object classes and interchangeable implementations of the same computational stage. This organization allows different reconstruction or tracking strategies to be selected without changing the surrounding workflow. Figure 2 provides a schematic view of these relationships and should be interpreted as a software-organization diagram rather than as a representation of the numerical processing pipeline or a benchmark of computational performance.

### 2.6. Imaging Acquisition and Parameters for Processing Tests

Diffusion-weighted images used for the clinical feasibility analysis (n = 8) were acquired on a single Siemens Aera scanner (Erlangen, Germany) (software syngo MR E11M 1.5T) using a single-shot spin-echo EPI diffusion sequence (TR = 2800 ms; TE = 86 ms; flip angle = 90°; pixel bandwidth = 1235 Hz). Slice thickness was 5 mm with 6.5 mm interslice spacing (a 1.5 mm gap) and in-plane resolution ranging from 1.3068 to 1.4205 mm (not strictly uniform across the 8 studies) (Supplementary File S2). The 5 mm slice thickness and non-isotropic acquisition are important limitations because subsequent interpolation or resampling cannot restore information that was not acquired at higher through-plane resolution. All 8 clinical studies were single-shell acquisitions at b = 1000 s/mm^2^ along 64 non-collinear directions, with 2–3 b = 0 volumes per series (96–131 volumes total). For each series, we recorded a standardized set of acquisition variables, including the exact b-value shells, number of b = 0 volumes, and phase-encoding direction, allowing the 8 clinical feasibility cases to be reported as a single uniform protocol. Effective echo spacing (0.445–0.455 ms) and total readout time (77.9–79.6 ms) were also recorded, but no field map or reverse phase-encoding acquisition was available for any of the 8 patients, so no susceptibility distortion correction was applied.

By contrast, the 30 technical cases were acquired across 8 different scanner models (Aera x15, MAGNETOM Sola Fit x5, SIGNA Explorer x3, SIGNA Prime x2, Avanto_DOT x2, Achieva x1, Optima MR360 x1, Signa HDxt x1) and therefore cannot be described as a single fixed protocol; acquisition parameters for this cohort varied by scanner and are reported at the series level. Fields marked as not recorded in the technical series DICOM headers, chiefly phase-encoding polarity on non-Siemens scanners, should be verified from the original scanner protocols before any prospective validation study. 

For the technical workflow, default processing parameters were otsu_radius = 1, otsu_numpass = 3, CSA_sh_order = 6, relative_peak_threshold = 0.7, min_separation_angle_peaks = 60°, normalize_peaks = True, step_size_streamlines = 0.5, threshold_mask_from_peaks = 0.08, seed_voxel_density = 3, fiber_min_length = 10, and fiber_max_length = 1000. These values were user-adjustable and could be modified according to the selected reconstruction model and clinical objective. ROI registration was performed using DIPY Symmetric Diffeomorphic Registration, AffineTransform3D, or TranslationTransform3D according to the anatomical level and registration task; RigidTransform3D was used for within-subject longitudinal registration. The processing pipeline included motion/distortion correction and denoising as available in the software, followed by model reconstruction, orientation estimation, mask/seed generation, tractography, and optional clustering/manual cleaning. Model-specific parameters were not prospectively harmonized across all cases; therefore, the present series should not be interpreted as a controlled comparison of diffusion models.

Potential limitations related to acquisition quality, preparation time, and heterogeneous clinical scanning were considered during implementation. Navimetric operates under ISO 13485 certification in Colombia, which provides a quality-management framework that includes risk-management processes during medical-device development. This certification is reported as a manufacturer-level quality-management attribute and should not be interpreted as evidence that Tractometric^®^ has undergone independent clinical validation or regulatory approval for a specific clinical indication.

### 2.7. Study Design and Scope

The present work was designed as a technical development and preliminary clinical feasibility report. The primary outcome was successful implementation of the complete Tractometric^®^ workflow, including diffusion-data loading, reconstruction, tractography, visualization, and export into the Surgimetric^®^ planning environment. Secondary descriptive outcomes were processing-time characteristics across selected operators and qualitative concordance between reconstructed tracts and intraoperative functional mapping in a small clinical series. No prespecified statistical hypothesis test, diagnostic-accuracy threshold, test–retest protocol, independent usability assessment, or formal health-economic analysis was performed. The retrospective series was assembled from available technical workflow and operative records rather than through prospective consecutive enrollment, and the available records did not support a standardized analysis of exclusion reasons or technical failures. Postoperative clinical observations were recorded descriptively and were not considered study outcomes.

The 30-case technical series characterized software use under representative clinical conditions, including variation in operator experience, reconstruction models, registration, ROI definition, and manual tract refinement. Its purpose was to describe workflow requirements and processing-time variability rather than assess accuracy, reproducibility, or usability. The DSI Studio (version “Hou”, 2025) comparison was retained as an exploratory technical reference to demonstrate implementation of comparable diffusion-processing tasks; however, because datasets and parameters were not prospectively standardized, it was not intended to establish anatomical equivalence or superiority. Intraoperative direct cortical and subcortical stimulation served as a qualitative functional reference for assessing the anatomical plausibility of reconstructed tracts, with concordant findings interpreted as supportive clinical context rather than formal validation of tract anatomy or diagnostic performance.

### 2.8. Technical Comparison and Clinical Reference

DSI Studio^®^ was included as a reference platform because it is an established dMRI and tractography environment [9]. The available retrospective comparison was exploratory rather than a formal validation experiment. A representative reconstruction was generated in Tractometric^®^ and DSI Studio^®^ using the available case data; however, the records did not provide a standardized, prospectively locked set of identical reconstruction models, seeding/ROI definitions, stopping criteria, streamline counts, or cleaning procedures across platforms. Consequently, processing time and workflow characteristics are reported descriptively, and no quantitative claim of anatomical equivalence or superiority is made. A formal software-validation study would require identical input data, harmonized parameters and ROIs, repeated blinded reconstructions, predefined spatial agreement metrics, and an independent reference such as a validated phantom, tracer dataset, or other ground truth [11,12,13].

## 3. Results

### 3.1. Diffusion Neuroimaging Algorithms

The system integrates a sequence of dMRI processing functions covering motion/distortion correction and denoising, diffusion-model reconstruction, orientation-peak estimation, mask and seed generation, deterministic or probabilistic fiber tracking, and tract filtering/clustering. Supported reconstruction frameworks include DTI, SHORE, GQI, CSA, and SfM, with model selection depending on the input acquisition and intended analysis. Masks may be generated automatically using threshold-based or Otsu procedures or manually using interactive tools. Tracking parameters include step size, angular constraints, stopping thresholds, seed density, and minimum/maximum fiber length. The final tractogram can then be filtered using anatomical ROIs, geometric criteria, clustering, and manual 2D/3D editing. These functions constitute the implemented software capabilities; they do not by themselves establish anatomical accuracy or inter-operator reproducibility.

The workflow was designed to reduce unnecessary manual steps, but manual ROI selection, atlas registration, streamline filtering, clustering, and visual cleaning remained possible and were used in the cases summarized below. Because the present users were members of or closely involved with the development team, the observed workflow times should be interpreted as technical characterization rather than as an independent usability assessment.

The principal data structures use NumPy arrays (version 2.2.4) and VTK PolyData, optimizing interoperability between numerical processing and 3D visualization. Memory management is handled through an intelligent caching system that temporarily stores intermediate results, allowing only modified processing steps to be recalculated. The software accepts DICOM, NIfTI, BVAL, and BVEC inputs, and supports export to VTK format in the dMRI space to store the output fibers, and to BIN format to store the transformation matrix describing the registration between the dMRI and the preoperative MRI, for seamless integration with Surgimetric^®^ surgical planning and navigation systems.

### 3.2. Workflow

**Figure 3:**
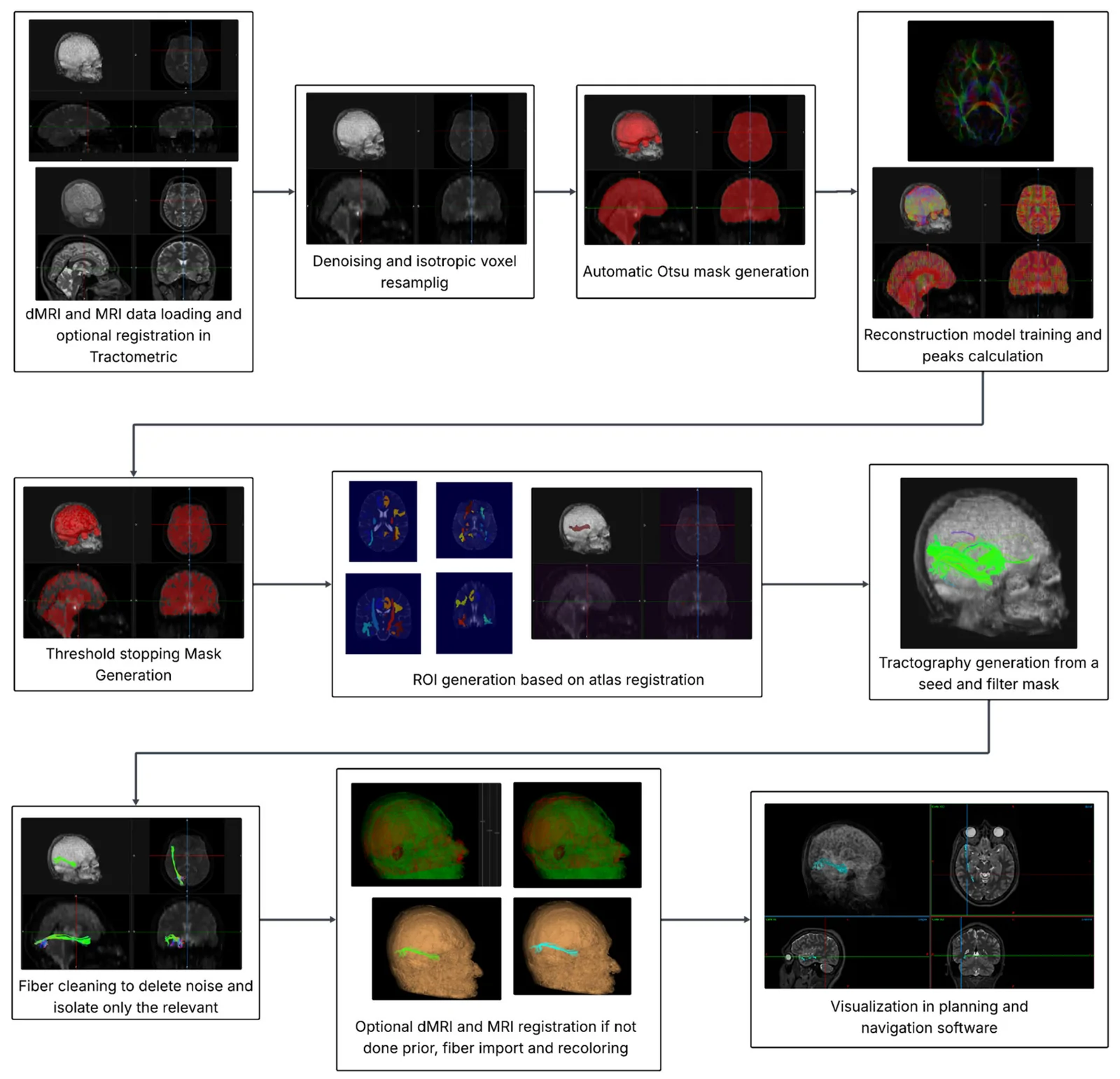
Representative Tractometric^®^ workflow from diffusion-data loading through mask generation, diffusion reconstruction, tractography, streamline refinement, and export to Surgimetric^®^. This figure documents the sequence of software operations and the transition from the diffusion-image space to the neuronavigation planning environment. Because several steps remain user-configurable, the example should be considered illustrative rather than a fixed protocol for all patients.

The Tractometric^®^ workflow comprises seven sequential stages: (1) loading and quality checking of diffusion and structural MRI data; (2) generation or selection of a brain/stopping mask; (3) selection and reconstruction of a diffusion model; (4) estimation of local orientation peaks and generation of tracking constraints; (5) definition of seed and filter ROIs, either manually or through atlas registration; (6) deterministic or probabilistic streamline generation followed by optional clustering and manual cleaning; and (7) export and fusion of the tractogram and registration matrix within Surgimetric^®^. Figure 3 illustrates these stages in a representative workflow. The sequence is flexible: users may return to earlier stages to change the model, ROI, or tracking parameters before generating the final tractogram.

#### 3.2.1. Data Loading

The user selects a directory containing DICOM files or NIfTI volumes. The system identifies the associated diffusion-gradient information from BVAL/BVEC files when available and checks the input structure before processing. Structural MRI volumes used for surgical planning can be loaded as anatomical references. When a preoperative MRI volume has already been registered within Surgimetric^®^, the corresponding transformation information can be stored for subsequent fusion of the tractogram.

#### 3.2.2. Mask Generation

Masks may be generated manually with interactive drawing tools or automatically using threshold-based and Otsu-based segmentation. These masks define the valid processing domain and, depending on the workflow, may also serve as seeding or stopping regions. Because mask selection can materially influence tractography results, the user remains responsible for reviewing the generated mask before proceeding.

#### 3.2.3. Diffusion Model Reconstruction

The user selects an available diffusion reconstruction model and its associated parameters. The model is fitted within the selected processing mask, after which local diffusion information is used to estimate orientation profiles. The software supports multiple reconstruction approaches, but the present study did not prospectively compare their accuracy under a common acquisition protocol.

#### 3.2.4. Directional Peak Estimation and Stopping Mask Generation

Local directional peaks are calculated from the reconstructed diffusion representation and are used to define candidate fiber orientations. A stopping mask can then be derived from diffusion-derived thresholds, including the implemented peak-based thresholding approach. The resulting constraints are reviewed before streamline generation because low signal, lesions, edema, resection cavities, and other anatomical abnormalities can affect the reliability of local orientation estimates.

#### 3.2.5. Region Definition and Tractography

Seeding regions may be defined manually from patient-specific anatomy or obtained from an atlas registered to the individual study. Atlas registration can use rigid, affine, or diffeomorphic transformations according to the selected anatomical level. A second ROI may be used as a filter region so that only streamlines satisfying the specified anatomical constraints are retained. The resulting tractogram is generated using the selected deterministic or probabilistic tracking strategy.

After streamline generation, the user can apply geometric or anatomical filters and inspect the result in 2D and 3D. The software supports automatic clustering based on spatial and geometric similarity as well as manual removal of outliers or anatomically implausible streamlines. This cleaning step is an important source of operator dependence and is therefore reported as part of the workflow rather than treated as an objective measure of software performance.

#### 3.2.6. Tractogram Export and Fusion with Preoperative MRI

The final tractogram can be exported as a VTK 3D object together with the transformation matrix required for fusion with the preoperative MRI environment. Within Surgimetric^®^, the tractography can be displayed with other imaging datasets and used as an additional anatomical planning layer. The present study evaluated the technical ability to complete this transfer and visualization; it did not prospectively measure whether the exported tractogram changed a surgical decision.

#### 3.2.7. Technical Series and Clinical Feasibility Subset

A retrospective technical series of 30 cases was reviewed to characterize workflow use and processing-time variability (Supplementary File S3). These cases were not analyzed as a formal reproducibility study because the available records did not include repeated blinded reconstructions of the same datasets under identical parameters or a prespecified agreement metric. Preliminary clinical feasibility was assessed in 8 patients who underwent tractography as part of neurosurgical planning, with intraoperative direct cortical and/or subcortical electrical stimulation used as a qualitative functional reference (Supplementary File S3). The clinical series was not designed to estimate sensitivity, specificity, false-positive or false-negative rates.

Among the 8 clinical cases, 3 had lesions adjacent to descending motor pathways, and 5 had lesions involving or adjacent to language-related networks. In the motor cases, positive cortical and subcortical stimulation findings were qualitatively concordant with the reconstructed corticospinal tract (CST). In the language cases, positive stimulation sites were qualitatively concordant with reconstructed trajectories of the arcuate fasciculus, superior longitudinal fasciculus (SLF), inferior fronto-occipital fasciculus (IFOF), and uncinate fasciculus, with stimulation producing language disturbances such as anomia, paraphasic errors, or grammatical impairment. The operative plan was reported as unchanged in the available series; therefore, these cases demonstrate workflow integration and qualitative anatomical-functional concordance rather than a measurable change in surgical strategy. Operative notes documented positive and negative stimulation findings; however, the retrospective records were not sufficiently standardized to permit quantitative site-level analysis. A standardized log containing all stimulation sites, stimulation intensities, coordinates, brain-shift correction, and tract-to-site distances was not available for retrospective quantitative analysis.

Postoperative observations were recorded descriptively. In the 8 reported cases, no postoperative neurological or functional deterioration was documented, and Karnofsky Performance Status (KPS) was described as unchanged in the immediate postoperative period. Preoperative and postoperative neuropsychological assessments were performed by a clinical neuropsychologist, and intraoperative evaluation included the Boston Naming Test and multitasking paradigms tailored to lesion location. These observations were not predefined study outcomes and were not used to infer clinical efficacy.

### 3.3. Technical Workflow Characterization and Preliminary Clinical Reference

The three sessions (Table 1), performed by a software developer, neurosurgeon, and biomedical engineer, demonstrated the practical workflow and operator-dependent processing requirements of tract reconstruction. Variations in reconstruction models, registration, ROI definition, and manual refinement affected processing times; therefore, the 30-case series was considered a technical workflow characterization rather than a formal reproducibility or performance analysis. The exploratory DSI Studio^®^ comparison provided a practical reference for diffusion-processing tasks but, given the lack of standardized parameters, matched ROIs, independent operators, and quantitative spatial measures, did not establish anatomical equivalence, accuracy, or superiority. In the eight clinical cases, qualitative concordance between reconstructed motor and language pathways and positive intraoperative stimulation findings provided preliminary clinical context and supported workflow integration but was not considered formal anatomical or diagnostic validation [11,12].

### 3.4. Illustrative Cases

#### 3.4.1. Case 1

**Figure 4:**
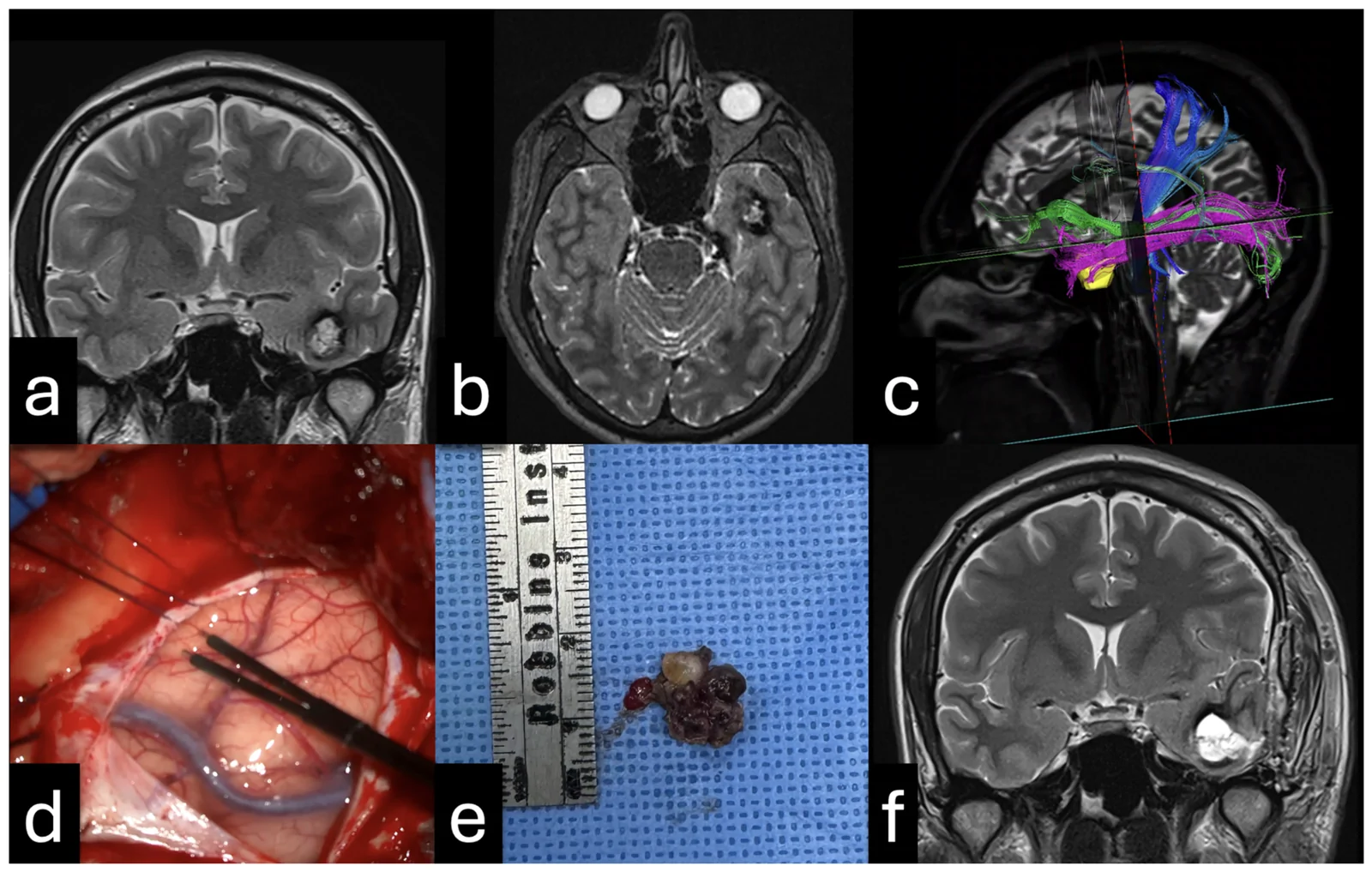
Illustrative integration of Tractometric^®^ tractography during resection of a left temporal cavernous malformation. (**a**,**b**) Preoperative MRI demonstrating the cavernous malformation and surrounding hemosiderin. (**c**) Patient-specific tractography of the inferior longitudinal fasciculus (purple) and inferior fronto-occipital fasciculus (green) integrated into the neuronavigation planning environment. (**d**) Intraoperative functional mapping using direct electrical stimulation. (**e**,**f**) Postoperative imaging demonstrating gross-total resection. The figure illustrates multimodal workflow integration and qualitative correspondence between tractography and functional mapping; it is not intended as a quantitative validation of tract location.

A 32-year-old man presented with a 3-month history of focal seizures. Neurological examination was unremarkable. Brain MRI demonstrated a left temporal cavernous malformation. Because the lesion was anatomically close to the left inferior longitudinal fasciculus (ILF) and IFOF, patient-specific tractography was reconstructed and incorporated into the preoperative planning environment (Figure 4). The patient underwent an awake (asleep-awake-asleep) craniotomy for image-guided resection. The tractography did not alter the planned trans-sulcal surgical corridor, but it provided an additional anatomical reference during cortical and subcortical mapping. Direct stimulation produced paraphasic errors when the inferior longitudinal fasciculus region was stimulated. Gross-total en bloc resection was achieved. A transient postoperative paraphasia improved after 2 months, and no further neurological decline was reported during 1 year of follow-up. This case illustrates technical integration and multimodal interpretation rather than a demonstrable improvement in outcome attributable to the software.

#### 3.4.2. Case 2

**Figure 5:**
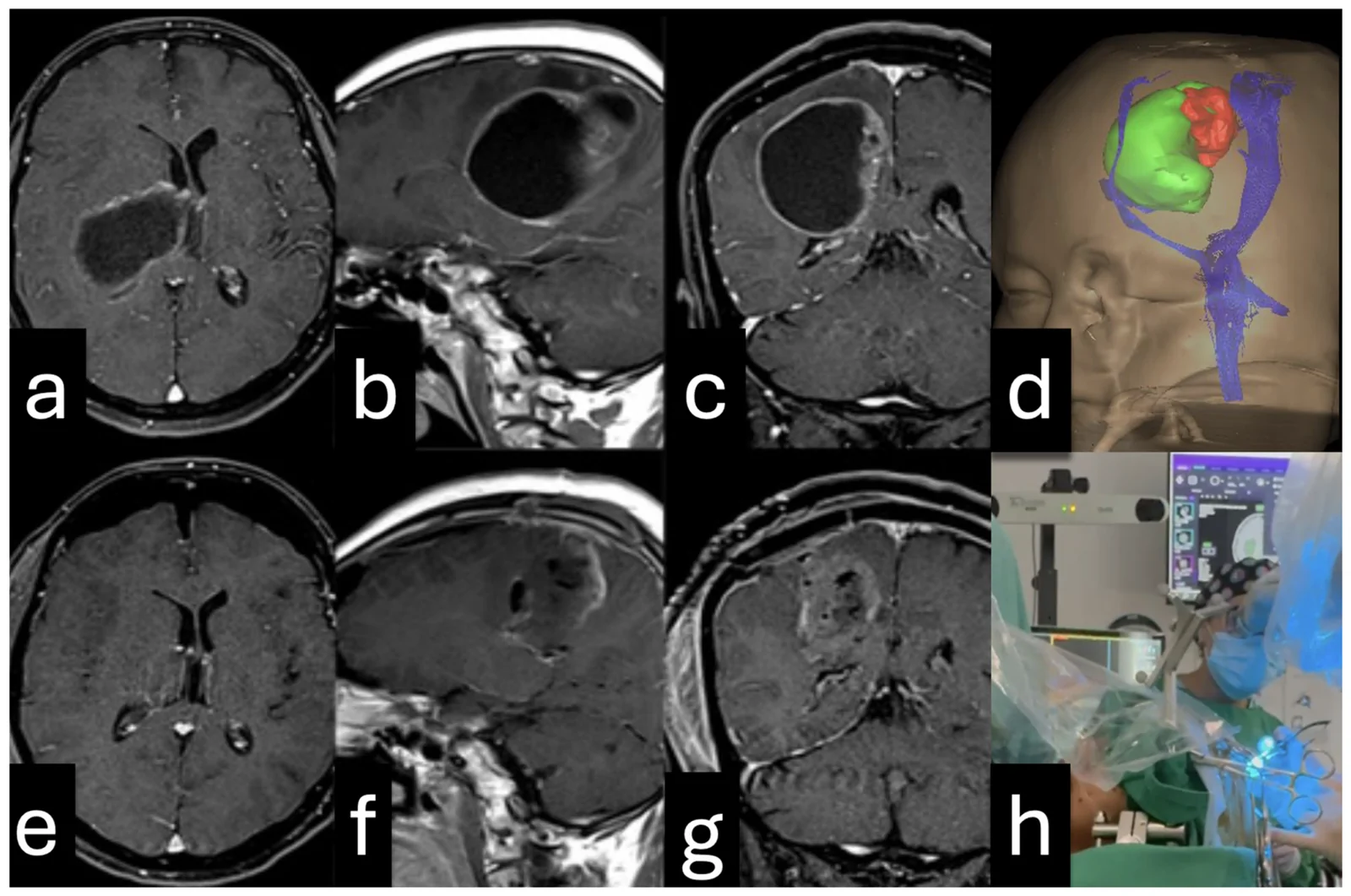
Illustrative integration of Tractometric^®^ corticospinal tractography during resection of a right frontal metastasis. (**a**–**c**) Preoperative contrast-enhanced MRI showing the lesion. (**d**) Patient-specific corticospinal tractography demonstrating distortion of the tract in relation to the lesion. (**e**–**g**) Postoperative MRI showing gross-total resection. (**h**) Intraoperative image-guided mapping and subcortical stimulation. The figure illustrates the technical use of tractography as an anatomical reference and its interpretation alongside intraoperative functional mapping; it should not be interpreted as proof of tractography accuracy or clinical efficacy.

A 68-year-old woman presented with confusion and left hemiparesis (2/5 strength) in the setting of a right deep frontal lesion concerning for metastasis. Her history included previously treated breast carcinoma. MRI demonstrated a lesion anatomically related to the CST, prompting patient-specific tractography (Figure 5). She underwent an awake craniotomy with image guidance and fluorescence-assisted resection. The reconstructed CST and its perirolandic endpoints were incorporated into planning and used as an anatomical reference during subcortical stimulation. Gross-total resection was achieved, and postoperative strength progressively improved over the following 3 months with physical therapy. Histopathology was consistent with metastatic breast carcinoma. As with Case 1, the tractography served as an additional planning and mapping reference; the case does not establish causal benefit or diagnostic accuracy.

## 4. Discussion

The principal contribution of this work is the development and clinical workflow integration of Tractometric^®^, a diffusion tractography software engine designed to transfer patient-specific tractograms into a neuronavigation planning environment. The scientific contribution of the present study should be interpreted at the level of technical implementation and preliminary clinical feasibility. The motivation for this development is particularly relevant in LMIC settings, where implementation of advanced imaging and navigation may be constrained by acquisition, software, infrastructure, maintenance, and training requirements [4,20]. The current study does not provide a formal economic evaluation and therefore does not establish that Tractometric^®^ is cost-effective or less expensive than complete commercial neuronavigation ecosystems. Its contribution should instead be understood as a technical approach intended to address workflow and interoperability barriers within the existing Surgimetric^®^ environment. The implemented algorithms support conventional DTI as well as several advanced diffusion reconstruction frameworks, but their comparative anatomical performance was not tested prospectively in this study.

The present findings are best interpreted as technical feasibility evidence. The 30-case retrospective series describes workflow use and processing-time variability rather than reproducibility, because repeated blinded reconstructions and quantitative agreement metrics were not performed. The 8-patient series shows that tractography could be incorporated into surgical planning and interpreted alongside intraoperative mapping; it was not designed to quantify diagnostic accuracy or clinical effectiveness. Positive stimulation findings therefore provide qualitative clinical context rather than an independent reference standard for complete tract anatomy. Prospective studies with standardized acquisitions, locked parameters, independent operators, systematic recording of positive and negative stimulation sites, and quantitative tract-to-reference metrics are needed to evaluate these outcomes [11,12,13,17].

### 4.1. Tractography and Neurosurgical Workflows

Diffusion tractography has become an increasingly important adjunct in neurosurgical planning because it can provide patient-specific information about the spatial relationship between lesions and white matter pathways [10,21,22,23]. Recent literature also emphasizes that the clinical utility of tractography depends on acquisition quality, model selection, ROI strategy, filtering, and multimodal interpretation, and that intrinsic limitations such as false-positive/false-negative streamlines and intraoperative brain shift remain important [10,11,12,13,24]. These considerations support the approach adopted here, in which tractography is presented as an additional planning layer rather than a stand-alone anatomical truth.

Clinical studies have reported potential associations between tractography-assisted planning and neurosurgical outcomes [22,25], but those observations do not establish that a particular software platform caused the outcomes. In the present series, operative plans were reported as unchanged and postoperative observations were not compared with a control group. The cases therefore illustrate integration of tractography with direct functional mapping rather than a measured effect on surgical decision-making or postoperative function.

Similarly, recent studies have demonstrated that advanced diffusion imaging techniques may extend beyond white matter reconstruction alone. For example, Klint et al. integrated DTI- and QTI- derived diffusion metrics into a navigated brain tumor biopsy workflow to investigate the microstructural characteristics of high-grade gliomas [26]. By combining diffusion MRI tractography with intraoperative fluorescence and neuropathology, the authors demonstrated that high-grade glioma tissue exhibited decreased anisotropy, kurtosis, and order parameter-related diffusion metrics compared with contralateral white matter, supporting the potential role of tractography for intraoperative tissue characterization and surgical guidance.

Additionally, different tractography platforms have demonstrated utility across multiple clinical applications. For example, Abbasian et al. utilized the MRtrix3 platform to preprocess diffusion MRI data and reconstruct white matter tracts for the development of a glioblastoma tumor spread map. This approach enabled the generation of a volumetric modulated arc therapy (VMAT) treatment plan targeting regions with a higher predicted probability of tumor spread along white matter pathways, highlighting the potential role of diffusion tractography in personalized radiotherapy planning [27].

Recent work has also highlighted the importance of reproducibility, interoperability, and quantitative validation across tractography pipelines [10,11,12,13,24]. DSI Studio, MRtrix3, DIPY, and SlicerDMRI provide complementary approaches to diffusion reconstruction and tractography [6,7,8,9]. The present implementation adds a workflow-oriented option focused on integration with Surgimetric^®^, but the study does not establish superiority over these platforms. In particular, differences in acquisition, reconstruction model, ROI placement, tracking thresholds, and manual cleaning can produce materially different tractograms, making uncontrolled software-to-software comparisons difficult to interpret.

### 4.2. Comparison with Available Software Platforms

Available tractography platforms differ substantially in their intended use, licensing model, degree of integration, hardware requirements, and regulatory context. For this reason, a direct numerical cost comparison between stand-alone research software, software modules embedded in commercial neuronavigation ecosystems, and complete navigation systems is not methodologically equivalent [28,29]. Table 2 provides a qualitative comparison of software characteristics and interoperability rather than a quantitative economic ranking. Tractometric^®^ is not presented as cost-effective based on the present data. A future health-economic analysis should include the complete implementation cost of the relevant ecosystem, including hardware, software licensing, maintenance, technical support, training, and integration.

Several limitations should be emphasized. First, the study did not include a formal reproducibility protocol. The 30 retrospective cases were heterogeneous with respect to reconstruction model, ROI strategy, registration, and manual cleaning, and the operators were not independent of software development. Preprocessing was operator-driven rather than a fixed pipeline: motion correction, denoising, and resampling are optional steps, so what was applied has to be stated per case. Future studies should include repeated blinded reconstructions by independent users, predefined tasks, operator training, completion/error rates, and quantitative agreement metrics. Second, the diffusion acquisitions were not prospectively harmonized. The technical series is retrospective and multi-center; its heterogeneity is a property of the material, not a design choice, and no conclusion about acquisition-dependent performance can be drawn from it. The available records do not provide complete series-level information for all variables required to reproduce each acquisition, including exact b-value shells, b = 0 count, phase encoding, susceptibility correction, and model-specific preprocessing settings. The relatively coarse through-plane resolution also limits anatomical interpretation, and interpolation cannot recover information lost during acquisition. In the clinical subset, slice thickness is 5 mm with 6.5 mm interslice spacing, a 1.5 mm gap; through-plane information that was never acquired cannot be recovered by interpolation or resampling, and this bounds the anatomical accuracy of any reconstructed tract. Additionally, all acquisitions are single-shell, at b = 1000 s/mm^2^; models of crossing fibers fitted to a single shell are limited by the data rather than by the software.

Third, no susceptibility distortion correction is possible: no field map and no reverse phase-encoding acquisition exist for any patient, so EPI distortion is uncorrected (the effective echo spacing and total readout reported above at least allow the magnitude of the residual distortion to be quantified). Diffusion gradient vectors are also not reoriented after motion correction, and the gradient table is not recomputed, so a residual orientation bias is possible in patients who moved appreciably. There is no dedicated eddy-current correction and no automated quality control; quality control was visual, through the 2D and 3D views.

Fourth, the DSI Studio comparison was exploratory and cannot be considered a validation experiment; it did not use a standardized, identical parameter set, matched ROIs, blinded operators, or quantitative spatial agreement measures. Fifth, direct cortical and subcortical stimulation provides valuable functional information but does not constitute a complete reference standard for subcortical tract anatomy. The present retrospective dataset lacked a systematic record of all positive and negative stimulation sites, stimulation intensities, spatial coordinates, brain-shift correction, and predefined tract-to-site distance thresholds. These limitations preclude estimates of sensitivity, specificity, or false-positive/negative rates. Sixth, the atlas-registration component may be affected by mass effect, edema, resection cavities, or other anatomical distortions given the nature of neuronavigation systems; future work should evaluate registration error quantitatively and consider deformable or learning-based approaches with independent reference data.

From a usability perspective, the current workflow remains partly dependent on expert judgment, particularly for ROI definition, atlas selection, streamline filtering, clustering, and final anatomical cleaning. The three operators represented members of the development or implementation team and therefore cannot provide an independent assessment of usability. Future evaluation should involve external neurosurgeons, radiologists, and biomedical users performing predefined tasks with standardized training and validated usability measures. Expanded documentation of atlas anatomy, functional correlations, and inter-atlas relationships would also improve transparency and clinical interpretability.

Finally, the clinical cases were selected from patients in whom tractography was incorporated into neurosurgical planning and therefore may be subject to selection and confirmation bias. The series is too small and uncontrolled to determine whether the platform changes operative decisions, reduces neurological deficits, or improves extent of resection. Future prospective studies should prespecify the clinical decision that tractography is expected to influence, record cases in which tractography is discordant with intraoperative mapping, and report unsuccessful or technically failed reconstructions. A trajectory-planning module and additional atlas functionality may be useful future developments, but these components require separate validation before being presented as clinically validated decision-support tools.

## 5. Conclusions

Tractometric^®^ is a diffusion tractography software engine developed to provide a modular processing workflow and interoperable transfer of patient-specific tractograms into a neuronavigation planning environment. In the present technical development and preliminary feasibility study, the platform successfully supported diffusion-data loading, reconstruction using multiple models, ROI-based tracking, tract refinement, visualization, and export to Surgimetric^®^. A retrospective series of 30 cases characterized workflow and processing-time variability, while 8 clinical cases demonstrated that tractographic information could be incorporated into surgical planning and interpreted alongside intraoperative cortical and subcortical mapping. These findings support technical feasibility and workflow integration. They should not be interpreted as evidence of anatomical accuracy, inter-operator reproducibility, independent usability, clinical efficacy, improved postoperative outcomes, or cost-effectiveness. Future prospective studies should use standardized diffusion acquisitions, locked processing parameters, independent operators, quantitative spatial validation against reference datasets, systematic recording of positive and negative stimulation sites, and predefined clinical endpoints. Until such data are available, Tractometric^®^ should be considered a technical and workflow-integration platform with preliminary clinical feasibility rather than a formally validated tractography or clinical decision-support system.

## Figures and Tables

**Table 1:** Descriptive workflow times and operator configurations in three Tractometric^®^ sessions. The sessions differed in operator experience, diffusion model, registration strategy, ROI generation, and cleaning approach; therefore, they are not directly comparable performance tests.

User	Previous Knowledge	Initial Time for Introducing Information to Tractometric *	Interpolation	Number of Registrations	Model	Generation Method	Cleaning Method
Developer	Main developer with software background	Total time 18:36 - 3:05 image load and registrations. - 3:01 otsu mask generation and cleaning. - 1:50 model generation and peaks calculation. - 3:30 Manual seed, threshold and filter mask generation. - 7:10 Streamlines generation and refinement.	Yes	3	CSA	Seed mask based on the highest peak values and a filter mask on the brainstem	Manual deletion of the outliers and iterative clustering to narrow down the fibers.
Neurosurgeon	Main medical consultant during development, has seen several planings and used the software in surgery	Total time 37:12 - 5:58 image load, interpolation and registration - 6:20 otsu mask generation - 2:33 model generation and peaks calculation - 20:31 threshold mask generation and ROI registration - 2:10 streamlines generation and refinement.	Yes	1	DTI	ROI atlas for seeding	Clustering to select closest fibers and manual cleaning.
Biomedical engineer	Surgimetric^®^ developer with access to the user manual and vague knowledge of the user interface and workflow.	Total time 47:57 - 5:10 image load, interpolation and registration - 7:00 otsu mask generation - 3:00 model generation and peaks calculation - 5:33 threshold and ROI generation - 20:18 initial streamlines generation and refinement - 9:16 filter mask generation - 3:50 final streamlines generation and refinement.	Yes	1	DTI	ROI atlas for seeding and filter mask on the brainstem.	Not documented in the available session record.

* These sessions are presented to illustrate workflow burden and the contribution of operator-dependent steps rather than to compare operator performance. Technical sessions were performed on hardware with Intel Core i7-12700 at 2100 MHz, 32 GB DDR5 RAM, and 512 GB SSD. Processing times are descriptive, include user-dependent operations, and should not be interpreted as benchmarks against other platforms.

**Table 2:** Qualitative comparison of selected diffusion tractography and neuronavigation ecosystems. The table is descriptive and does not constitute a cost-effectiveness analysis or a statement of regulatory equivalence.

Platform	Access Model	Advanced Diffusion Models/Frameworks	Neuronavigation Integration	Input/Export Compatibility	Clinical-Use Context
DSI Studio [9] https://dsi-studio.labsolver.org/ (accessed on 20 August 2026	Open-source research plat form	DTI, GQI, QSDR	Export-based workflows; integration depends on receiving system	DICOM, NIfTI, TRK/FIB	Widely used in diffusion MRI research and clinical studies; regulatory/intended-use status should be verified for the specific application.
MRtrix3 [8] https://www.mrtrix.org/ (accessed on 20 August 2026	Open-source research platform	DTI, CSD, FOD and related tools	Export-based integration	DICOM, NIfTI, .tck	Extensive research use; clinical implementation depends on local workflow and regulatory requirements.
SlicerDMRI [7] https://dmri.slicer.org/ (accessed on 20 August 2026	Open-source research platform	DTI and HARDI/ODF-based methods	Export/DICOM-based workflows	DICOM and supported research formats	Research and translational clinical workflows.
Brainlab https://www.brainlab.com/ (accessed on 20 August 2026	Commercial neuronavigation ecosystem	Diffusion/tractography options depend on installed modules	Native integration within commercial navigation ecosystem	DICOM and proprietary formats	Established clinical navigation ecosystem; exact capabilities depend on configuration.
StealthViz/Medtronic (Medtronic)	Commercial ecosystem	Publicly available details vary by product/configuration	Native integration within Medtronic ecosystem	DICOM and proprietary formats	Commercial clinical ecosystem; exact tractography and regulatory capabilities should be verified for the installed configuration.
Tractometric	Proprietary software engine developed by Navimetric	DTI, SHORE, GQI, CSA, SfM	Designed for export/integration with Surgimetric^®^	DICOM, NIfTI, BVAL/BVEC, VTK, BIN	Technical development and preliminary clinical feasibility reported in the present study; independent validation remains necessary.

Platform characteristics are based on information available in the current manuscript and on the documented capabilities of the cited software ecosystems. Licensing terms, integration options, and regulatory status may change over time and should be verified directly with the relevant manufacturer or project before procurement or clinical implementation.

## Data Availability

Data available on request from the authors. The data that support the findings of this study are available from the corresponding author upon reasonable request, due to privacy and legal restrictions.

## Supplementary Materials

The following supporting information can be downloaded at: https://www.mdpi.com/article/10.3390/brainsci16101123/s1, Supplementary File S1: Tractometric: User Manual; Supplementary File S2: Diffusion MRI acquisition and processing report: series-level description; Supplementary File S3: Technical series and Clinical series information.

## Abbreviations

The following abbreviations are used in this manuscript:

AI	artificial intelligence
AR	augmented reality
BVALs	b-values
BVECs	b-vectors
CSA	constant solid angle
DICOM	Digital Imaging and Communications in Medicine
DIPY	diffusion imaging in Python
dMRI	diffusion magnetic resonance imaging
DTI	diffusion tensor imaging
FA	fractional anisotropy
GQI	generalized Q-Sampling imaging
HICs	high-income countries
LMICs	low- and middle-income countries
MRI	magnetic resonance imaging
MVC	Model–View–Controller
NifTI	Neuroimaging Informatics Technology Initiative
ROI	region of interest
SfM	Structure from Motion
SHORE	simple harmonic oscillator-based reconstruction and estimation
3D	3-dimensional
