# Supplementary material for: Tractometric: A Diffusion Tractography Software Engine for Integration into a Neuronavigation System: Technical Development and Preliminary Clinical Feasibility
Source: Brain Sci. 2026 Oct 17;16(10):1123. doi: 10.3390/brainsci16101123 (PMC13702410; doi:10.3390/brainsci16101123)

Tractometric - User Manual

Etiquetas	Versión PDF
Versión de último cambio	1.0.10

✅ This is the static user manual for the tractography module of the Surgimetric application.
To access the online version (with navigation and real-time updates), scan the following QR code or visit https://navimetric-latinoamerica.notion.site/

- Key Objects and Concepts
  - Conventions in this section
  - Mask
    - Manual mask
    - Otsu median mask
    - Threshold mask
    - Automatic ROIs
  - Model
    - Diffusion Tensor Imaging (DTI) model
    - Simple Harmonic Oscillator Reconstruction and Estimation (SHORE) Model
    - Generalized Q-sampling imaging (GQI) model
    - Scattered Fiber Model (SFM)
    - Constant Solid Angle (CSA) model
  - Peaks
    - Differences between the parameters
  - Tractography
    - Cluster / Subgroup of Tracts
- Interface description
  - Output Folder Selector
  - File Selector
  - Main panel
  - View panel
- Mask editing tools
  - Drawing tools
    - Pointer
    - Brush
    - Eraser
    - Fill
  - Editing Tools
    - Rotations
    - Expand | Contract
    - Clear

- Save tools
  - Save
  - Load
- Using automatic regions
- User tutorial (simple interface)
  - 1. Load the resonance data
    - 1.1. Upload the DICOM files
    - 1.2. Load the data one by one
    - 1.3. Upload data from a folder
  - 2. Generate model and peaks
    - 2.1. Choose type of tractography
  - 3. Generate tractography
    - 3.1. Group the tracts to clean them
  - 4. Load the tractography into the scheduler
    - 4.1 Files generated by the program
    - 4.2. Fuse diffusion resonance with a planning study
    - 4.3. Select the color of the tracts and locate them in the planner
    - 4.4. Clean the tracts
- User tutorial (advanced interface)
  - 1. Load the MRI data
    - 1.1. Loading DICOM Files
    - 1.2. Loading data one by one
    - 1.3. Loading data from a folder
  - 2. Generate a mask
    - 2.1. Drawing the manual mask
    - 2.2. Generate Otsu Mask
  - 3. Train a model
  - 4. Generate Peaks
  - 5. Generate a threshold mask
    - 5.1. Draw a seed mask
  - 6. Generate a tractogram
    - 6.1. Grouping the tracts to clean them up
  - 7. Load the tractography into the planner
    - 7.1 Files generated by the program
    - 7.2. Merge the diffusion MRI with a planning study
    - 7.3. Select the color of the tracts and place them in the planner
    - 7.4. Clean up the tracts
- List of automatic regions
  - Deep white matter fiber bundles
  - DiFuMo Atlas (64 dimensions) (v1)
  - DiFuMo Atlas (128 dimensions) (v1)
  - DiFuMo Atlas (256 dimensions) (v1)
  - Stanford HARDI
  - AAL Atlas
  - Destrieux 2009
  - Harvard-Oxford
  - Jülich
  - Pauli 2017
  - Schaefer 2018
  - Fiber bundles from the HCP dMRI dataset
  - Jülich-Brain Cytoarchitectonic Atlas (v1.18)
  - Julich-Brain Cytoarchitectonic Atlas (v2.9)

Julich-Brain Cytoarchitectonic Atlas (v3.0.3)
Julich-Brain Cytoarchitectonic Atlas (v3.1)
MarsAtlas cortical parcellation model
Von Economo-Koskinas Brain Atlas
Desikan-Killiany Atlas (2006)

Key Objects and Concepts

▼ Conventions in this section

The application is based on the creation and modification of certain objects described below; each object will be described using the following structure:

- The folder will be used to describe different objects that are interchangeable within a type.
- The object contains a description of what it is, the types it can be, and the objects needed to generate it

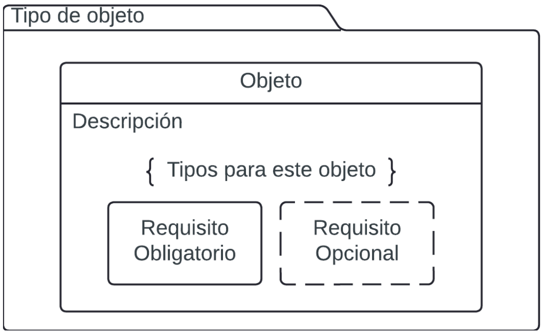

Mask

The mask restricts the area where processing occurs within the object that uses it; it is used to avoid processing the edges or noise in the image.

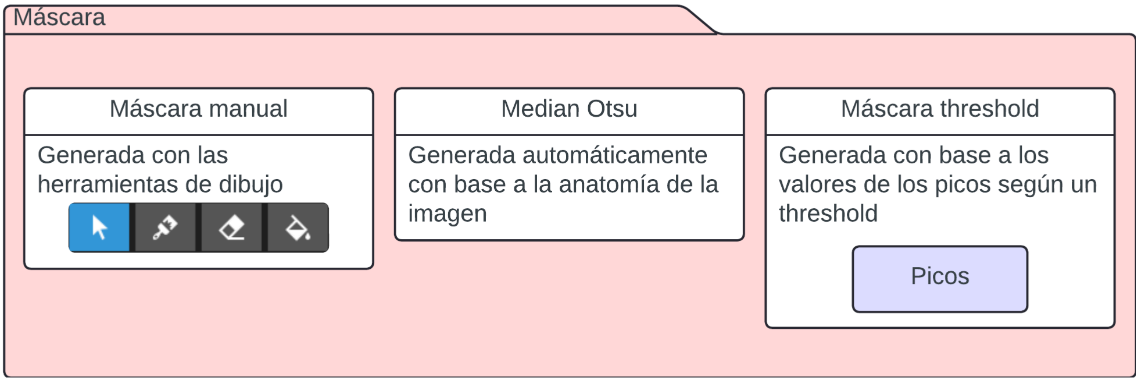

💡 Masks can be exported and imported from NIFTI files generated in the application or from applications that export a compatible format; any mask can be used to limit models or for tractography seeds

Manual mask

The manual mask is generated as blank when the image is loaded and can be edited using the tools in the mask tab to select regions of the study or specific points on a layer

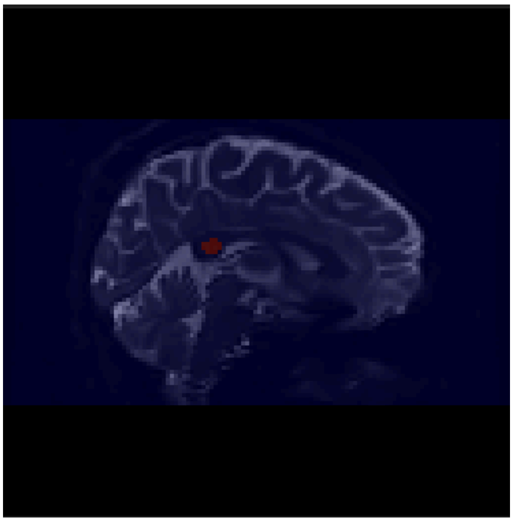

Máscara en un punto con herramienta manual

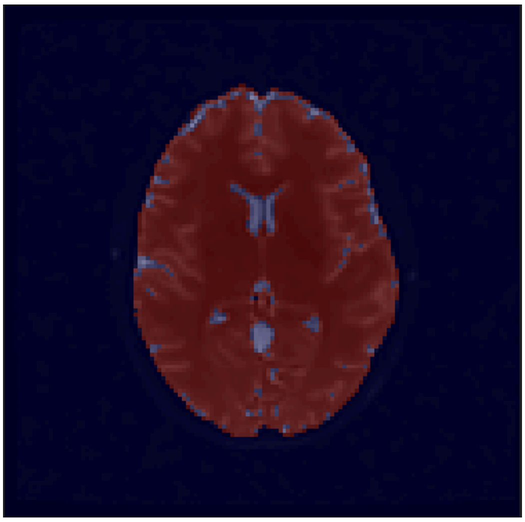

Máscara manual según threshold de color de la imagen creada con la herramienta de fill

> See Herramientas de edición para máscara

Otsu median mask

This mask is generated based on the anatomy of the loaded image and attempts to automatically cover the entire brain; when generating it, the parameters allow you to adjust the algorithm to cover more or fewer details, modify the smoothing, and expand the area during generation.

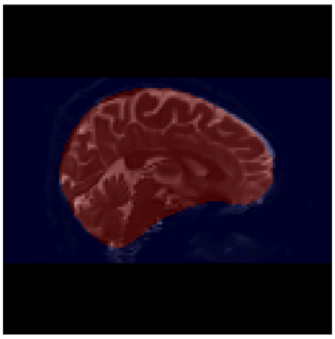

Máscara generada con radio y # pasadas altos

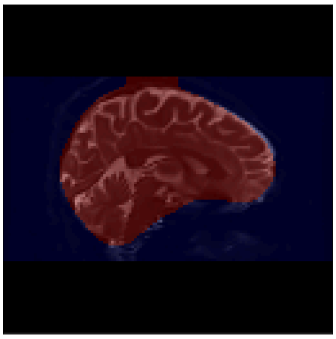

Máscara generada con radio bajo y # de pasadas alto

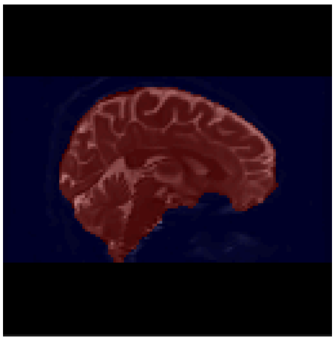

Máscara generada con radio bajo y # de pasadas alto

📢 This mask can be useful for filtering out noise values at the edges in subsequent steps of the tractography process, but you must ensure that it covers important areas

Threshold mask

This mask is generated similarly to the fill tool but based on the peak values generated from a model, so it can be more precise when selecting voxels that meet the condition

💡 The threshold value depends on the peak values; therefore, if they were normalized, the threshold will be in the range of 0 to 1

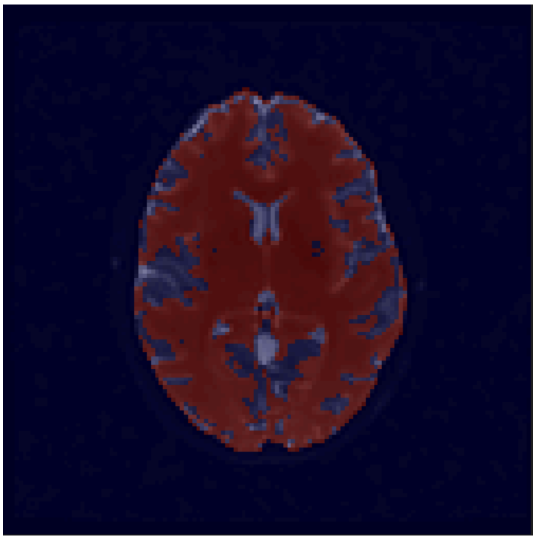

Máscara con un threshold bajo

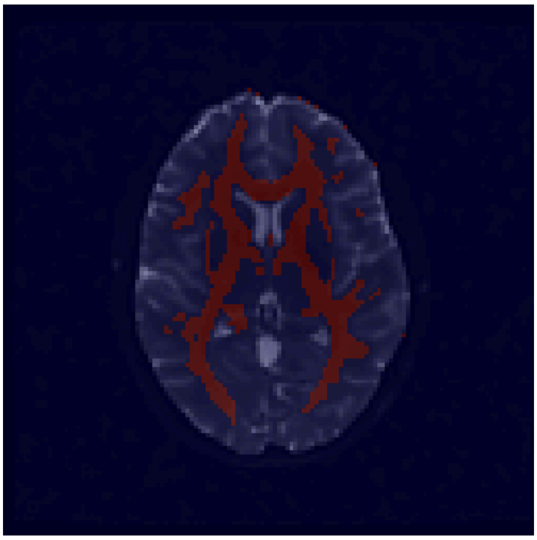

Máscara con un threshold alto

📢 Generating this mask is mandatory for tractography because it will be used as a stop criterion for the tracts.

Automatic ROIs

When automatic tractography is selected, masks can be generated for predefined anatomical regions; these are adapted to the patient's uploaded study through transformations that, in order, are **rigid** → **affine** → **diffeomorphic**. When generated, these regions are added as masks, and the program allows tractography to be generated for all created regions collectively. (See more at 🌐 Uso de regiones automáticas)

The transformations applied to the dataset are as follows:

- **Rigid:** Maintains the shape of the original dataset and attempts to register it to the loaded study using simple translations

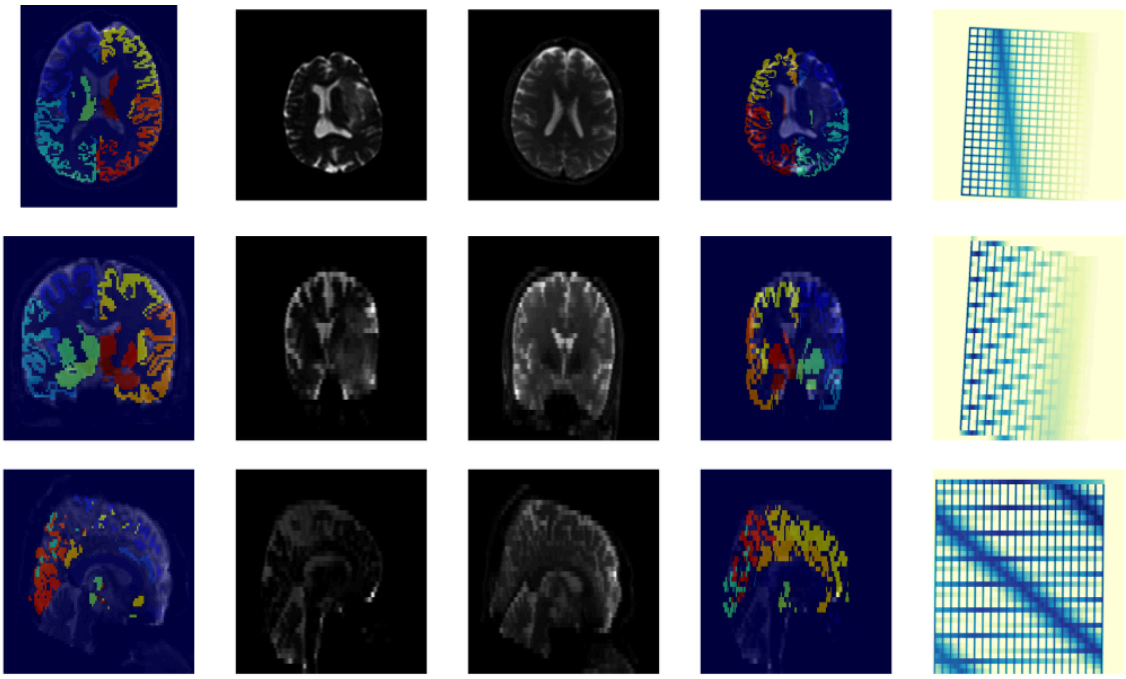

- **Affine: Performed** after the rigid transformation, it attempts to uniformly deform the original dataset to make it as similar to the study as possible

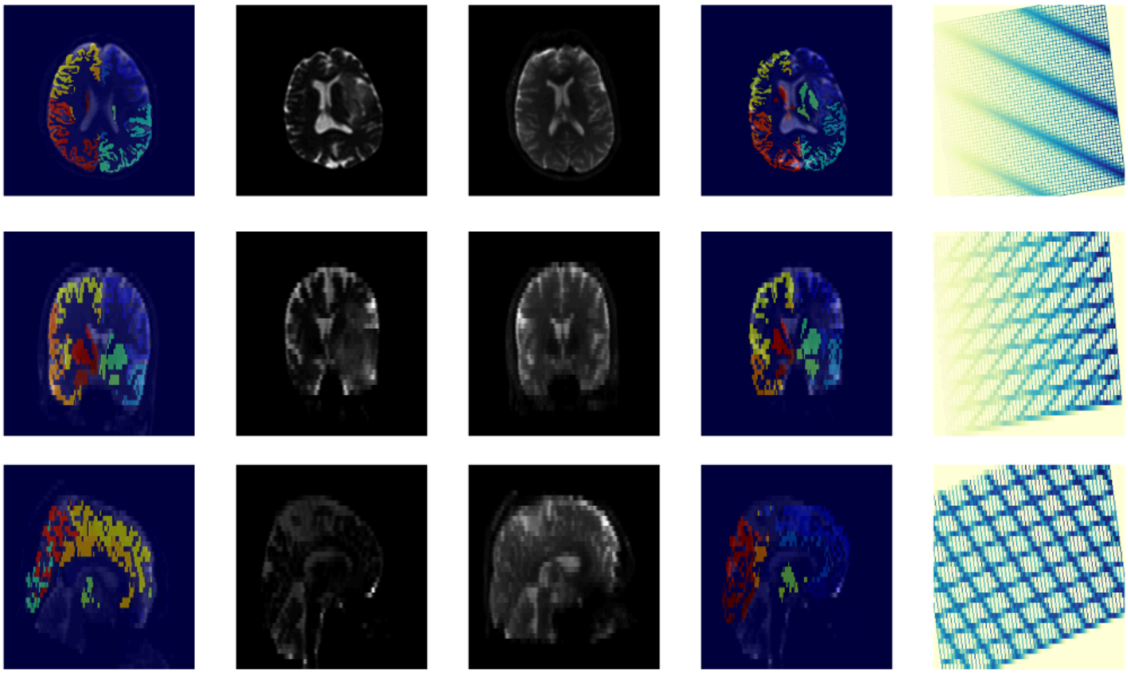

- **Diffeomorphic:** Performed after the affine transformation; as the final step, it irregularly deforms the volumetric image of the dataset so that the regions conform to the patient's anatomy, using the values (intensity) of both images as a reference

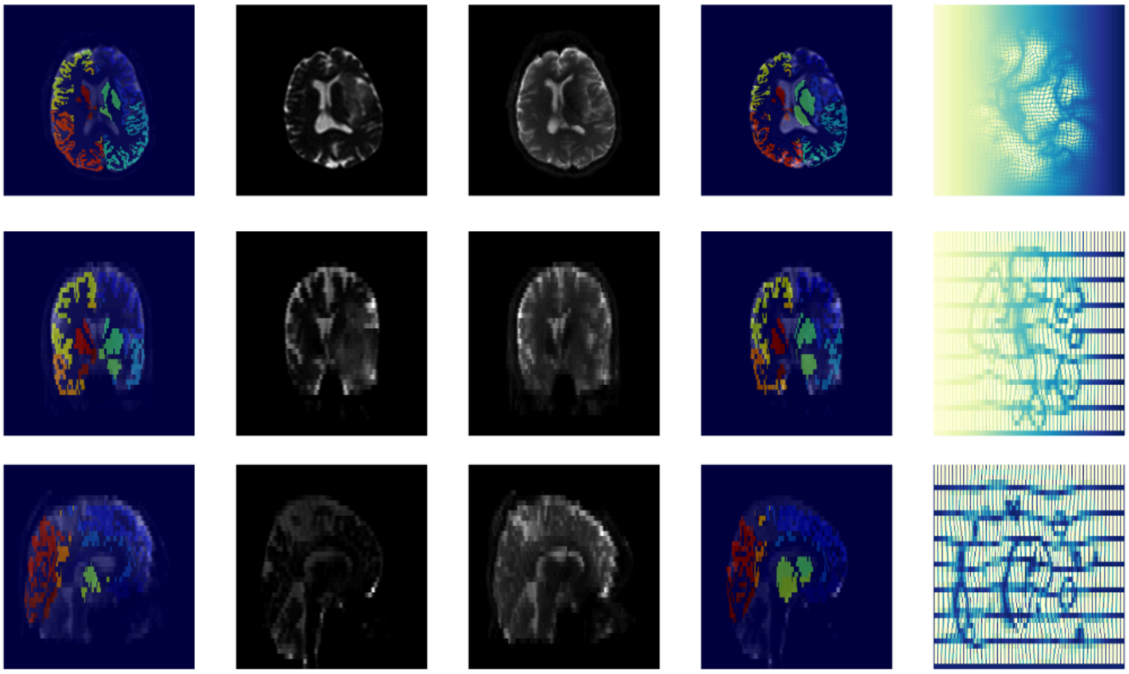

> ⚠️ It cannot be guaranteed that automatic registration will be perfect for studies where the patient's anatomy is severely compromised; it is advisable to verify the created regions and evaluate their validity on a case-by-case basis

Model

The model is the method used for diffusion flow reconstruction; it is essential for tractography, and once generated, it can provide information about the patient.

Below is a list of the models and their results with an example image

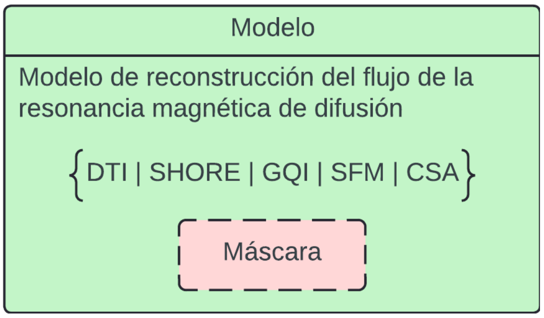

▼ Diffusion Tensor Imaging (DTI) model

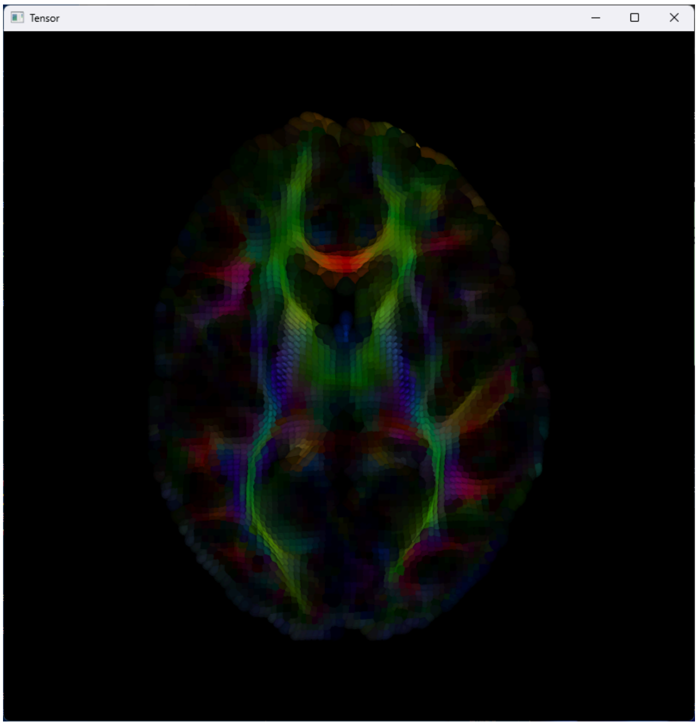

DIPY — dipy 1.9.0 documentation

All the models in the reconst module follow the same template: a Model object
is used to represent the abstract properties of the model, that are independent
of the specifics of the data . These properties are reused whenever fitting a

https://docs.dipy.org/stable/reference/dipy.reconst.html#dipy.reconst.dti.TensorModel

▼ Simple Harmonic Oscillator Reconstruction and Estimation (SHORE) Model

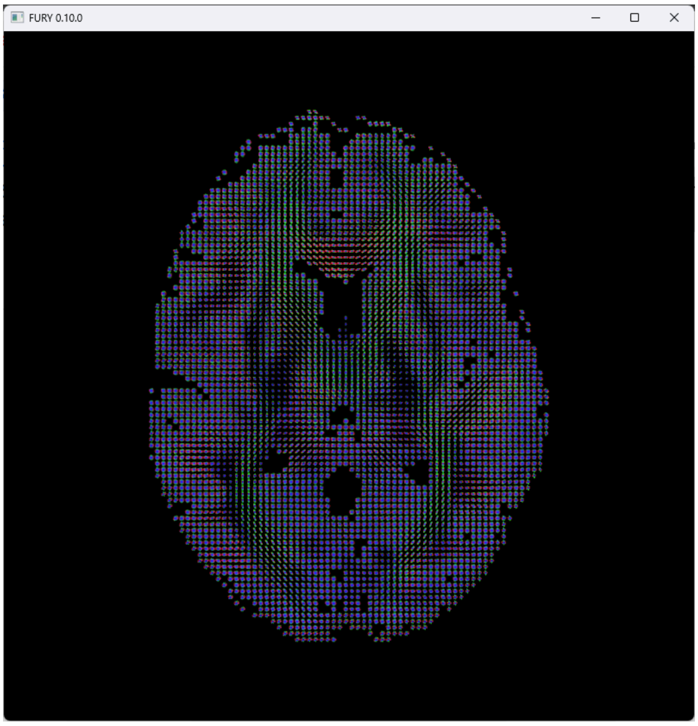

DIPY — dipy 1.9.0 documentation

All the models in the reconst module follow the same template: a Model object is used to represent the abstract properties of the model, that are independent of the specifics of the data . These properties are reused whenever fitting a

https://docs.dipy.org/stable/reference/dipy.reconst.html#shoremodel

▼ Generalized Q-sampling imaging (GQI) model

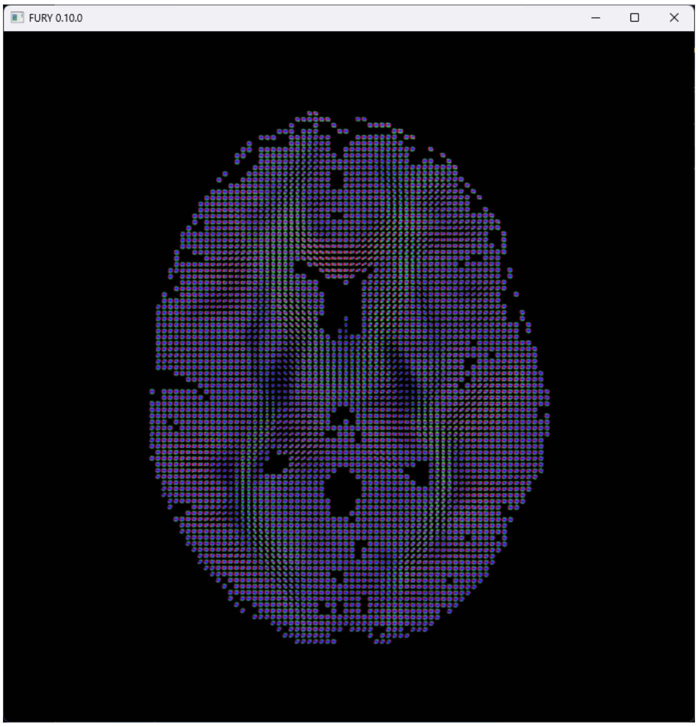

DIPY — dipy 1.9.0 documentation

All the models in the reconst module follow the same template: a Model object is used to represent the abstract properties of the model, that are independent of the specifics of the data . These properties are reused whenever fitting a

https://docs.dipy.org/stable/reference/dipy.reconst.html#dipy.reconst.gqi.GeneralizedQSamplingModel

▼ Scattered Fiber Model (SFM)

⚠️ This model has long training times

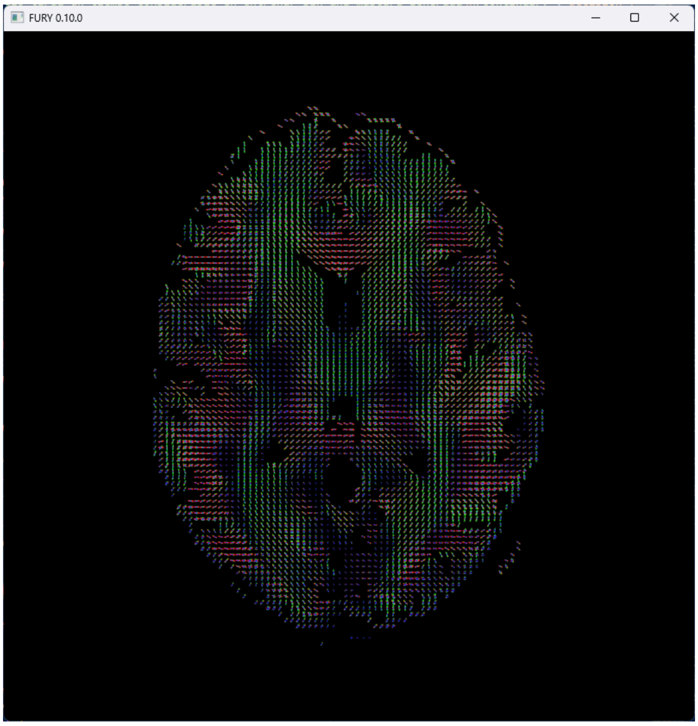

DIPY — dipy 1.9.0 documentation

All the models in the reconst module follow the same template: a Model object is used to represent the abstract properties of the model, that are independent of the specifics of the data . These properties are reused whenever fitting a

https://docs.dipy.org/stable/reference/dipy.reconst.html#dipy.reconst.sfm.SparseFascicleModel

▼ Constant Solid Angle (CSA) model

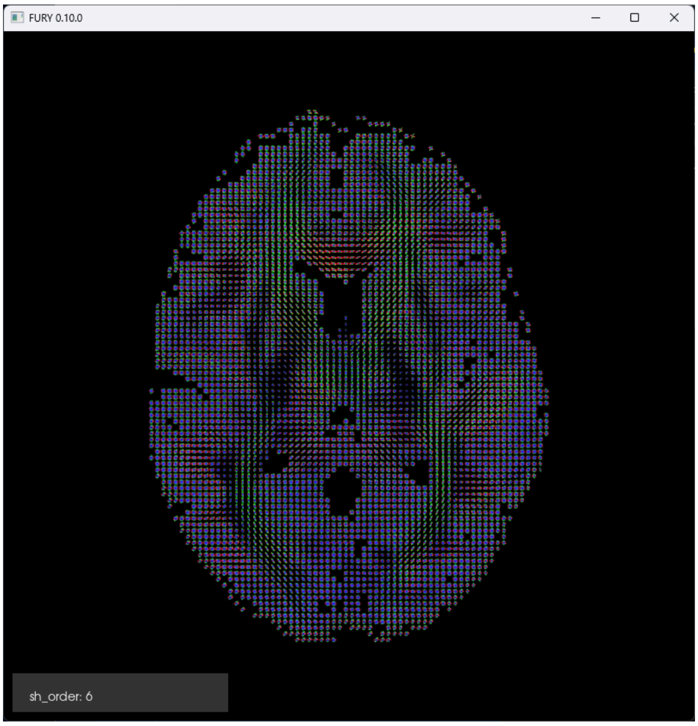

DIPY — dipy 1.9.0 documentation

All the models in the reconst module follow the same template: a Model object is used to represent the abstract properties of the model, that are independent of the specifics of the data . These properties are reused whenever fitting a

https://docs.dipy.org/stable/reference/dipy.reconst.html#dipy.reconst.shm.CsaOdfModel

Peaks

The peaks represent the flows obtained based on the trained model, and tractography is performed based on these. When generating them, you must choose the model to be used for generation.

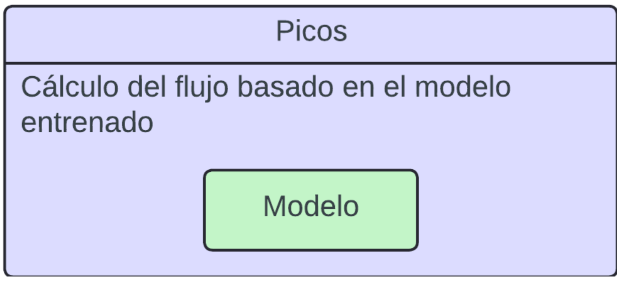

Once generated, they can be previewed at the layer or global level within the application, and their quality depends on the trained model and the image quality.

Differences between the parameters

- **Model**: The model used to generate them

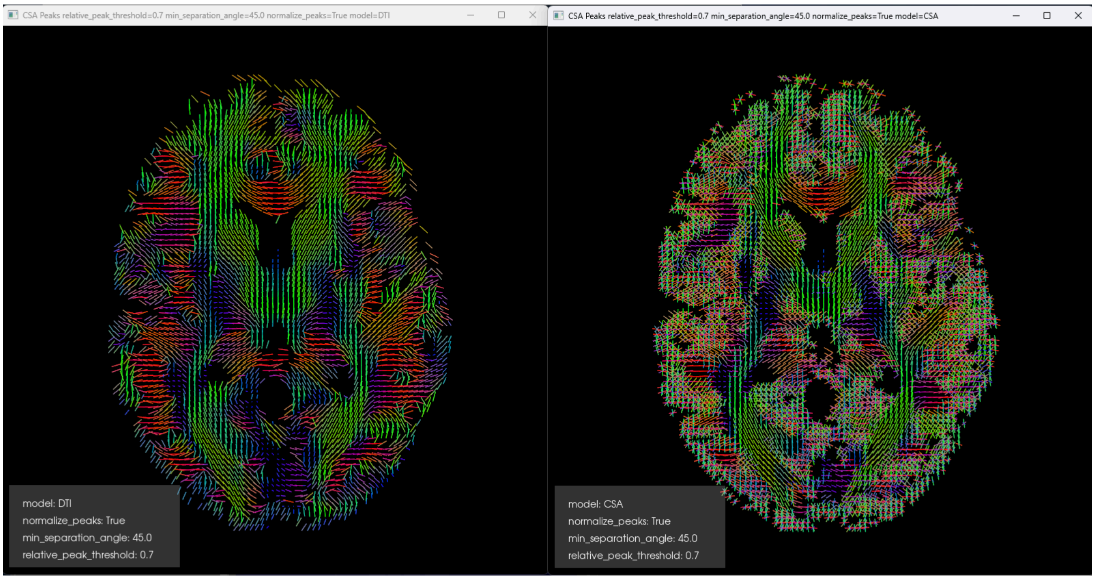

Comparación de los picos de un modelo DTI y uno CSA, muestra que el DTI transforma el ruido en tensores basado al promedio por lo que es menos preciso

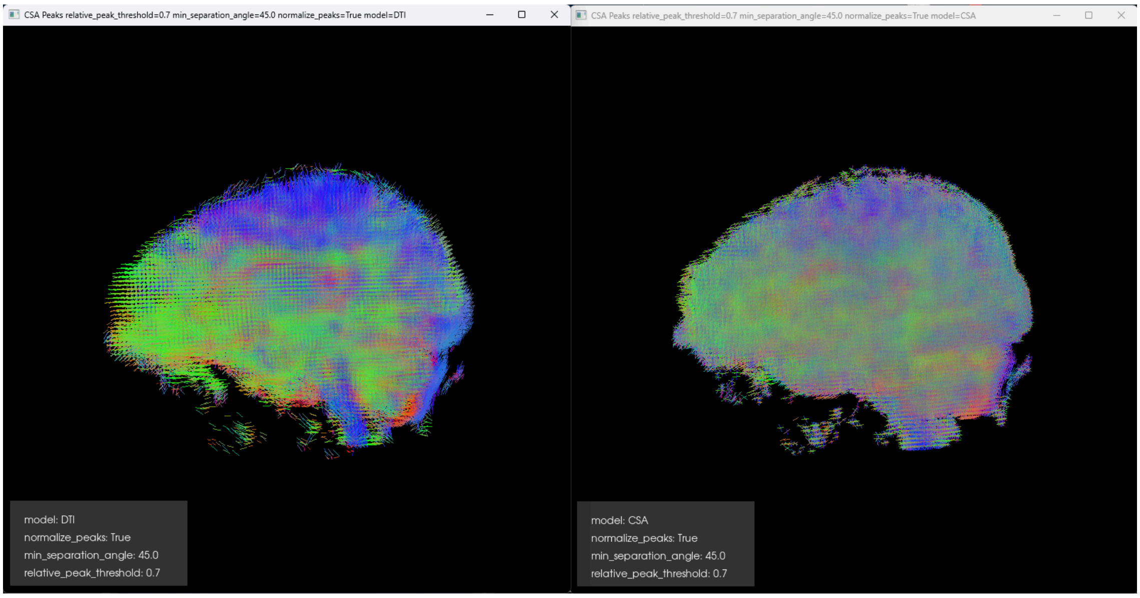

- **Relative threshold**: the threshold for filtering peaks [0,1]

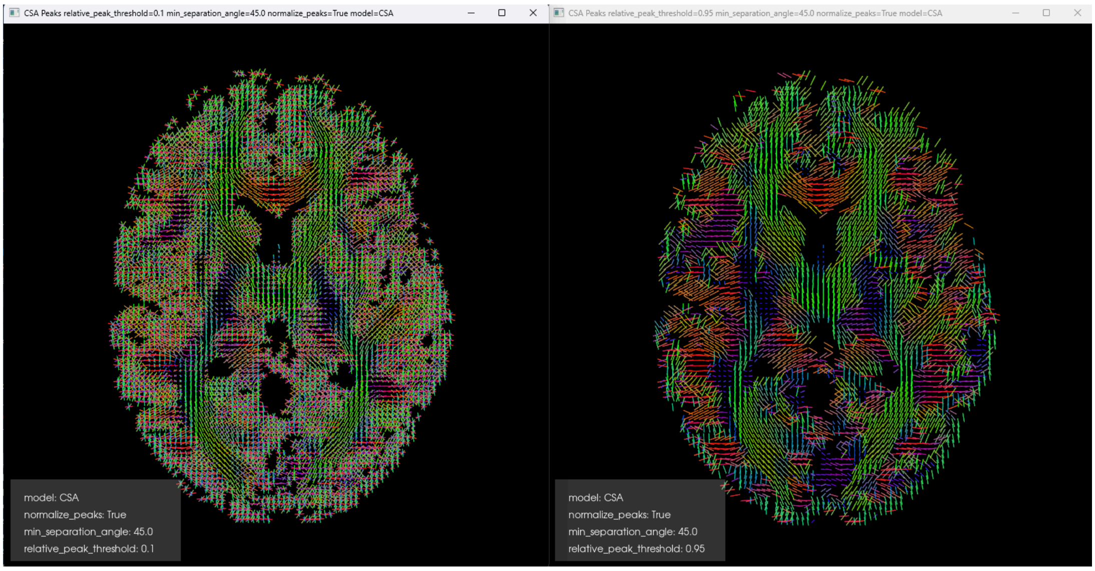

- **Minimum separation angle**: The minimum distance between two directions; if two peaks are below this value, only the larger one is considered [0.90]

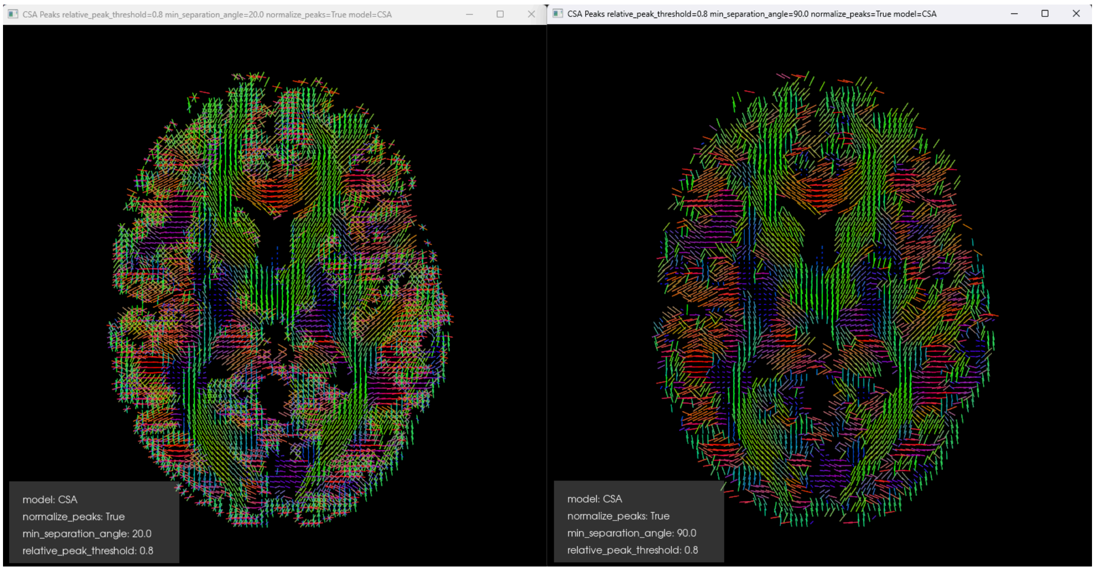

- **Normalize values**: If selected, all peaks are calculated relative to `max(odf)`

> ⚠️ If not normalized, visualizations may vary in clarity from lines too short to be visible to lines too long to be visible
>
> ▼ Example
>
> 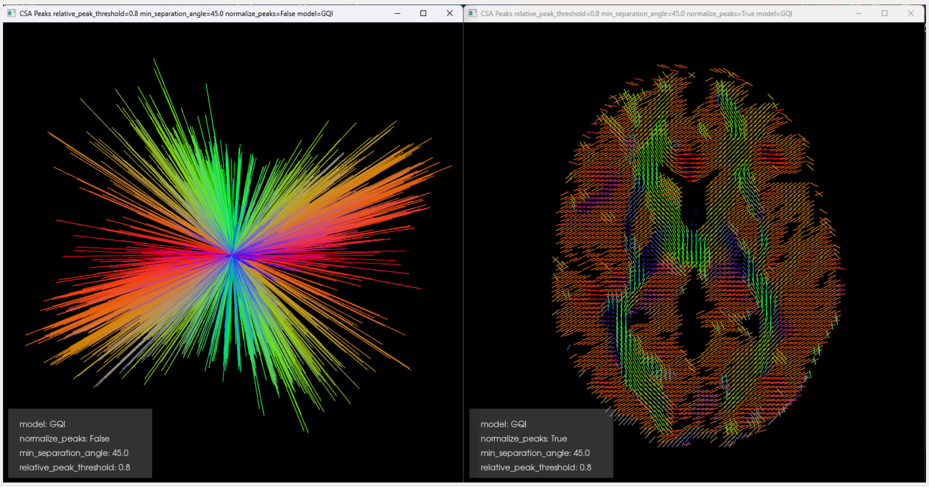
>

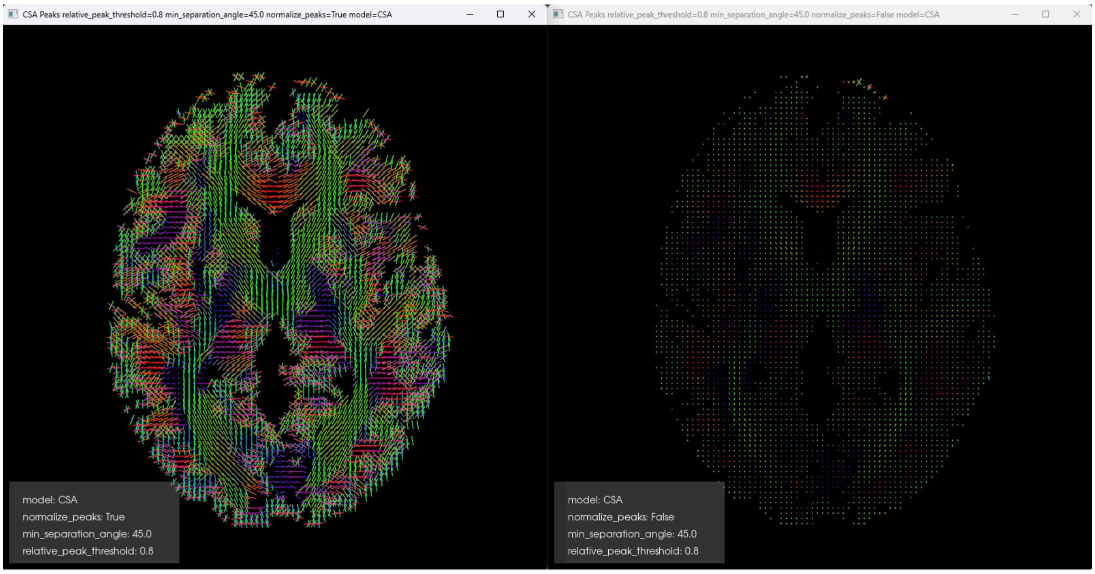

Tractography

Deterministic tractography can be generated once the peaks and the threshold mask are generated, and it displays the continuous tracts found from a seed

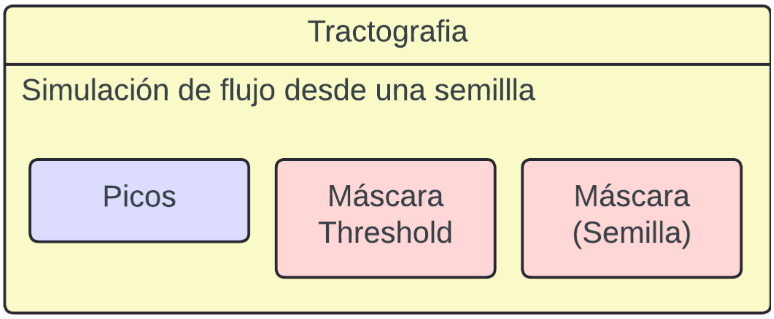

When generating it, any mask can be used for the seed; masks covering an area equal to or larger than the threshold mask will result in a complete tractography, and depending on the image, generation time may increase considerably.

💡 If a small mask is used, the tractography will be performed locally, and it is recommended to increase the density for greater clarity, since, unlike large masks, this does not significantly affect the generation time.

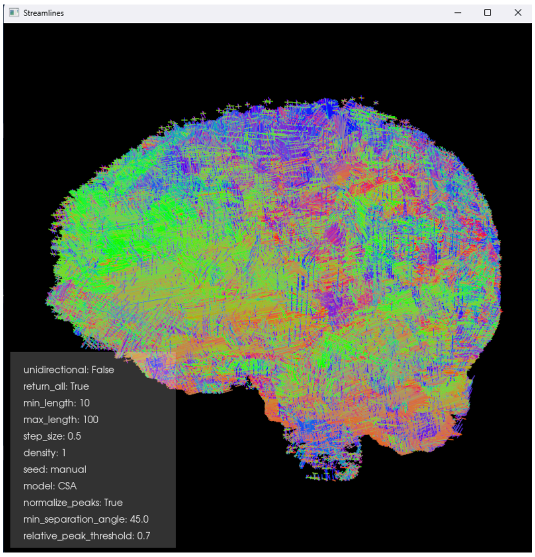

Tractografia total

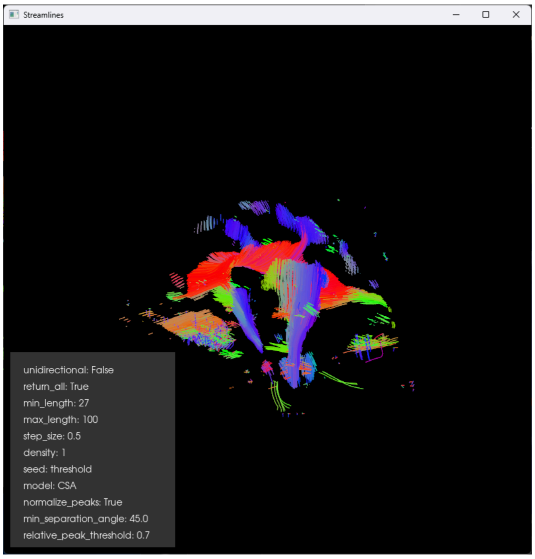

tractografia de las zonas con los picos más altos

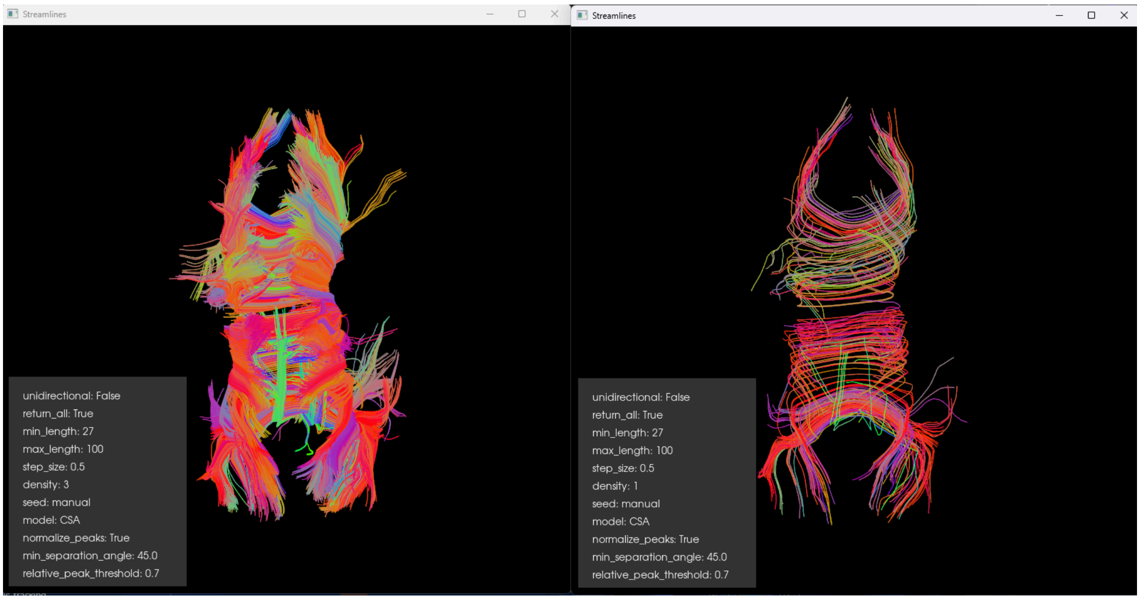

Tractografia hecha con una máscara manual y diferentes densidades

Once the tractography is generated, a histogram showing the distances of the tracts is displayed in the interface; this can be used as a guide to filter the minimum and maximum distances of the tracts by changing the `"Return all tracts` " parameter and adjusting the threshold values

Like masks, the results of a tractography can be exported and loaded into the application, as well as loaded into the main navigation module.

Cluster / Subgroup of Tracts

A cluster is a subgroup of tracts generated based on their similarity

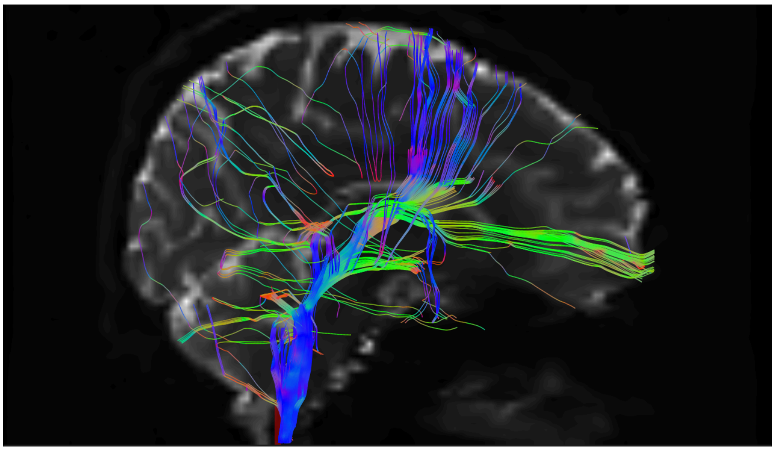

Grupo completo de tractos

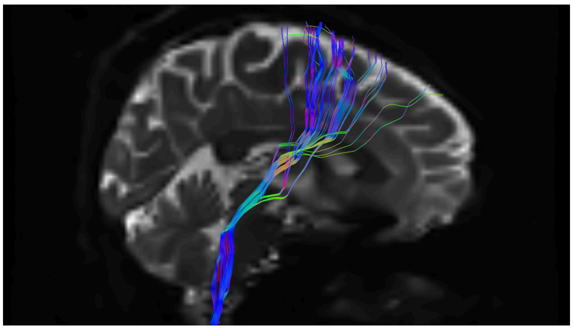

Clúster 1 generado de los tractos

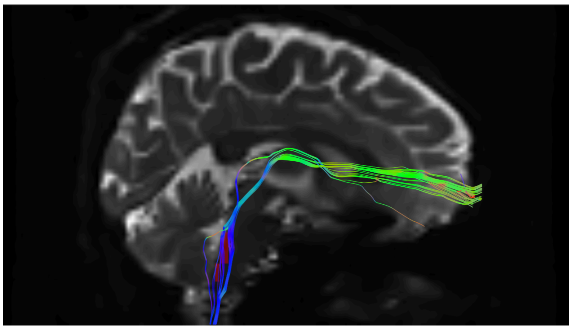

Clúster 2 generado de los tractos

When clustering the tracts, the program will take the specified target number and perform an iterative process until the desired number of subgroups is reached or until the number of subgroups stops increasing; thus, it will retain the highest number of subgroups that does not exceed the target value.

After clustering, the results will filter out subgroups with very few fibers and those where the fibers are very short to avoid insignificant groups and noise generated by the clustering process.

Interface description

💡 Within the interface, you can hover your mouse over buttons or titles to display an explanation of the parameter or section

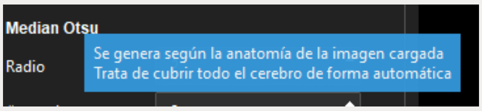

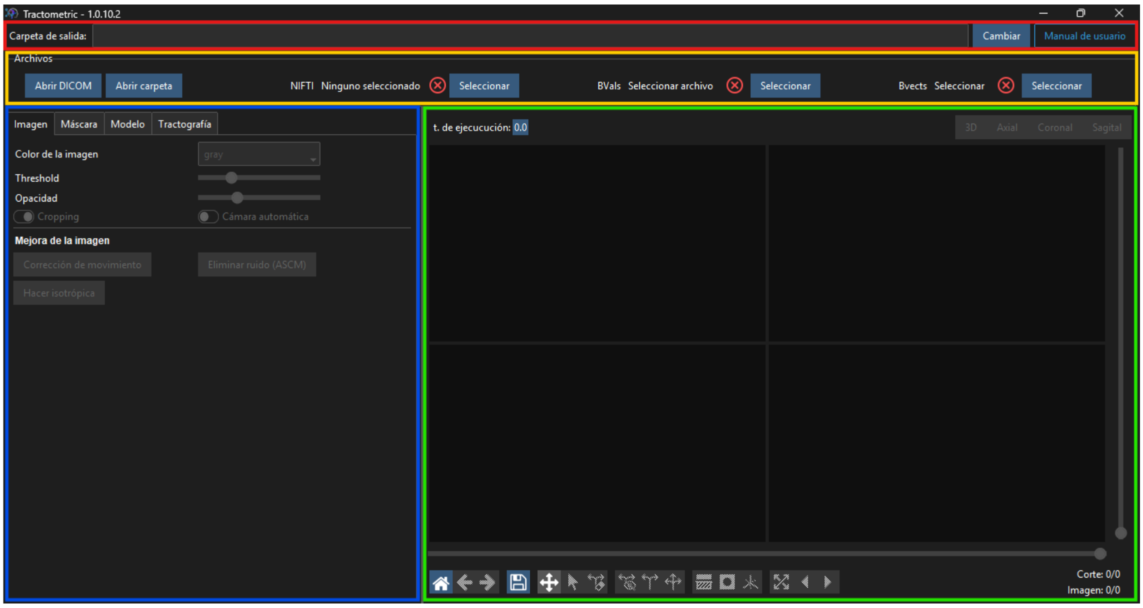

The interface is divided into 4 main sections:

Output Folder Selector

Displays the current output folder and allows you to change it using the `Change` button; clicking it opens a folder selector in the catalog where you must select the folder for the patient for whom planning is being performed.

It also has a `User Manual` button that links to this manual.

File Selector

Contains the buttons to load diffusion MRI files

Clicking the `Open from DICOM` button opens a dialog to select a folder containing DICOM files; once selected, the necessary conversions will be performed and the files will be exported to the selected or automatically set output folder.

Using the `Select` buttons for NIFTI, BVALS, and BVECTS will display a window to select a .nii, .nii.gz, or .txt file; the respective file must be selected.

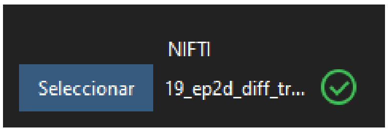

Botón para cargar el archivo NIFTI

If the three selected files are valid, the program will notify you; if not, it will display an error window with the reason.

Alternatively, if the three processed MRI files are in the same folder, you can click the `Open from Folder` button and select the folder containing the three files.

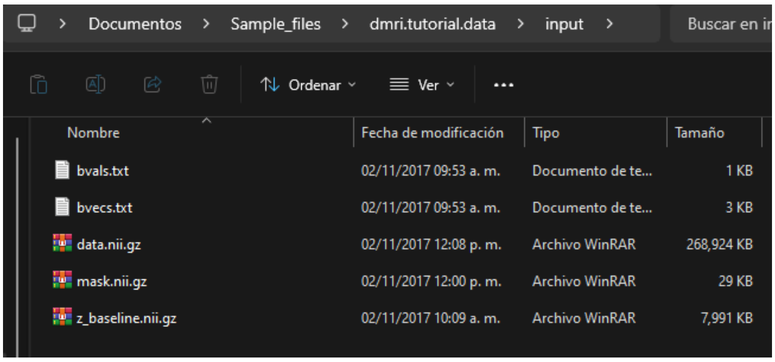

Ejemplo de carpeta con los tres archivos en ella

This is equivalent to selecting them one by one.

Main panel

> This panel differs between the simple and advanced interfaces; in the simple interface, some options are missing, and all available options are located on the same tab

This panel is divided into 4 tabs:

- Image: Contains options for editing the image view
  - Color: Palette for 2D views
  - Threshold: Percentage of low values to be ignored in the 3D volume
  - Opacity: The opacity of the 3D volume
  - Cropping: Crop at the current point in the volume
  - Image enhancement:
    - Motion correction: Corrects motion in a series by blending each volumetric image together; execution time will depend on the number of images in the series
    - Noise Reduction (ASCM): Removes noise from the resonators using the ASCM algorithm
    - Make Isotropic: Resamples the image so that the voxels have the same representative distance; this helps with reconstructions if one of the axes has very low resolution compared to the others. **It is important that if this option is to be used, it be done before performing tract reconstructions**.

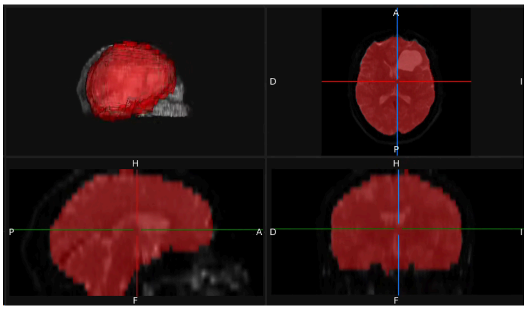

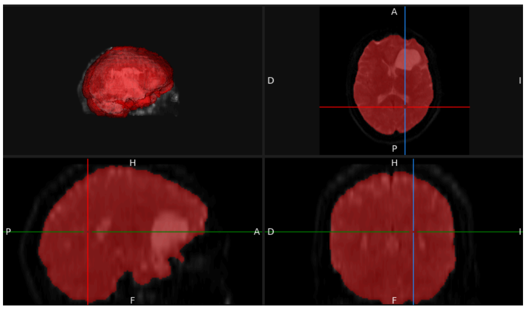

- Mask:
  - 1: Color picker to display the mask
  - 2: Slider for transparency
  - 3: Active mask selector; this is used as a basis for generating models, peaks, and threshold masks, and can also be used as a seed for tracts
  - 4: Herramientas de edición para máscara
  - 5: Options for generating an Otsu mask
  - 6: Options for generating a threshold mask

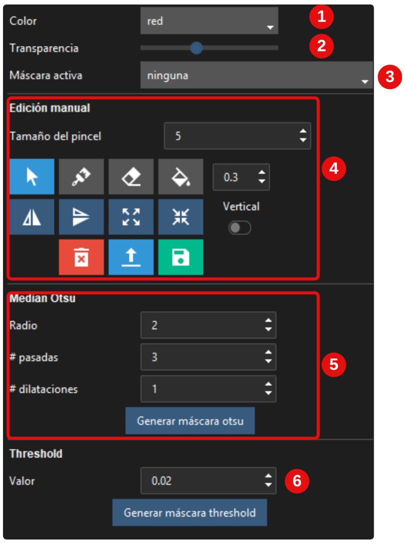

- Model:
  - List of models with their options, buttons to generate and display in 3D
  - Options for generating peaks, the model selector used to generate them, the button to generate peaks, and the selector for active generated peaks
- Tractography:
  - Selector for automatic or manual tractography
  - Options for manual tractography
    - Selector for the mask to be used as a seed
  - Options for automatic tractography
    - Selector for dataset to use
    - Region of interest selector
    - Selector for the type of transformation to perform
  - Density selector for tractography
  - Tract generation options
  - Button to generate the tractography
  - Active tracts selector (Allows switching between those generated in the session)
  - Selector for the number of target clusters and group button
  - Buttons to view the tractography in 3D and save it
  - Histogram of generated tracts by distance for filtering

View panel

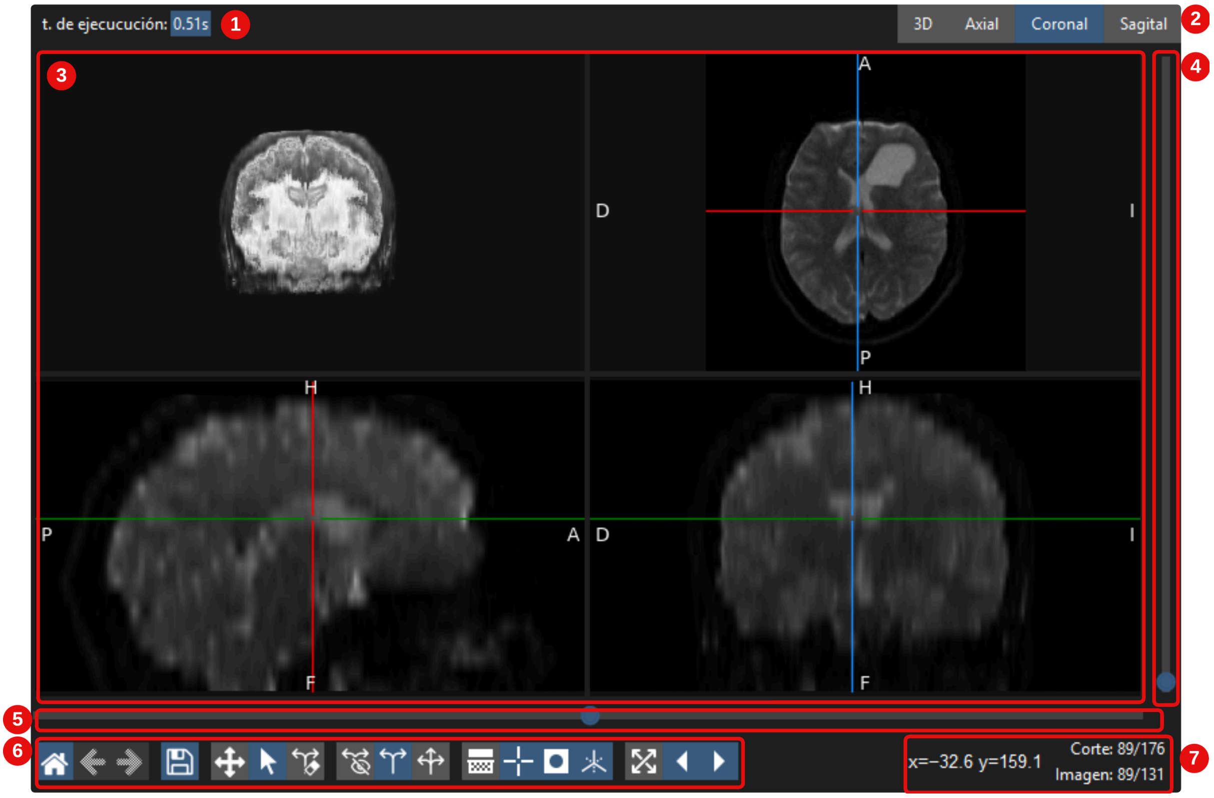

1. Timer for the last task executed
2. Active view selector
3. 3D, axial, coronal, and sagittal views
4. Slider to change the current series
5. Slider to change the current slice
6. Toolbar for modifying views
    - Buttons to change the view state: The home button resets the active view, and the arrows move to the previous/next states
    - Button to save the current view: Opens a file explorer to save the view as an image
    - Button to activate the move tool: Activates move mode; left-click to move and right-click to adjust the zoom
    - Button to activate the selection tool: When active, clicks change the point displayed in the views
    - Button to activate the fiber deletion tool: When active, it locks the 3D camera and allows you to delete fibers by clicking on them in the 2D and 3D views
    - Buttons to toggle view options: Enables and disables interpolation, shows and hides guide crosses, shows and hides mask, shows and hides peaks
    - Buttons to toggle 2D tract views:
        - Hide tracts
        - Show tracts in the current slice
        - Show complete tracts projected in 2D

- Buttons for full-screen view of the active view and to switch sections ◀ ▶

7. Information view of the current image

Mask editing tools

To edit masks, there is a set of tools designed to help select both masks for studies and seeds for tractography

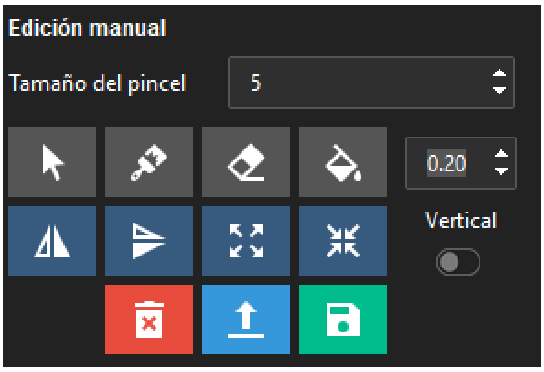

Herramientas para editar máscaras

> All tools will edit the `active mask`

Drawing tools

These tools work in conjunction with the Move tool; only one can be selected at a time

Pointer

The Pointer is the default tool, and its function is to provide a state in which clicking on the view will not change anything

Brush

The brush allows you to draw the mask over the current image crop in a circular area of the size specified in `Brush Size`. This tool is useful for making small modifications to automatic masks and for generating the seeds used for manual tracings

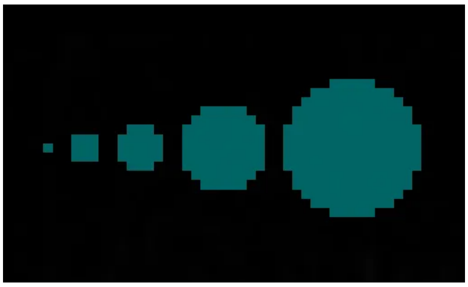

Pincel de tamaño 1, 2, 3, 5 y 8

> Right-clicking uses the `Eraser` tool

Eraser

Like the brush, the eraser uses the `Brush Size` and allows you to edit the current crop, but its function is the opposite of the brush's; clicking removes the area from the mask

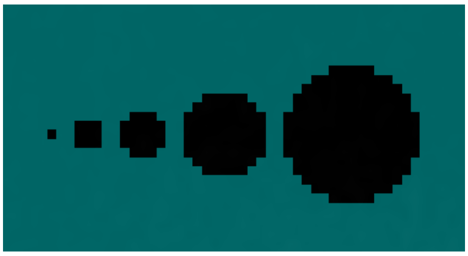

Borrador de tamaños 1, 2, 3, 5 y 8

> Right-clicking activates the `Brush` tool

Fill

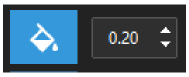

> This tool affects multiple selections

The Fill tool automatically paints the area of the image that has a similar color based on the threshold indicated in the number selector next to it. Adjusting the threshold is essential for using this tool effectively.

Holding down the mouse button and dragging can help select the area if it is very large or if you want to select the entire brain

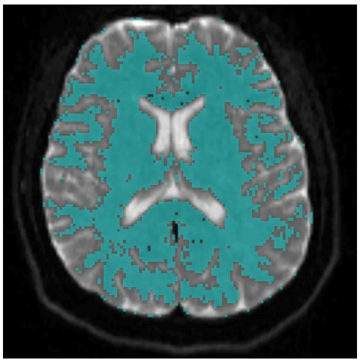

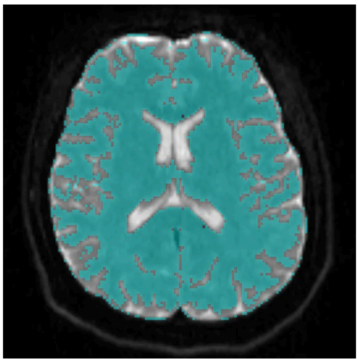

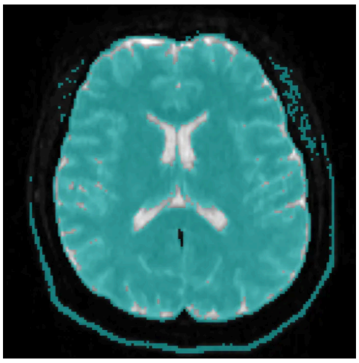

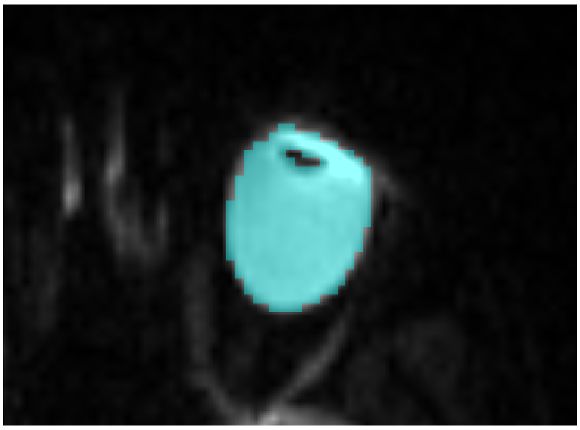

selección de un ojo con la herramienta rellenar

> Just like the `Brush` and `Eraser` tools, right-clicking has the opposite effect of a normal click; in this case, it removes the selected area from the mask

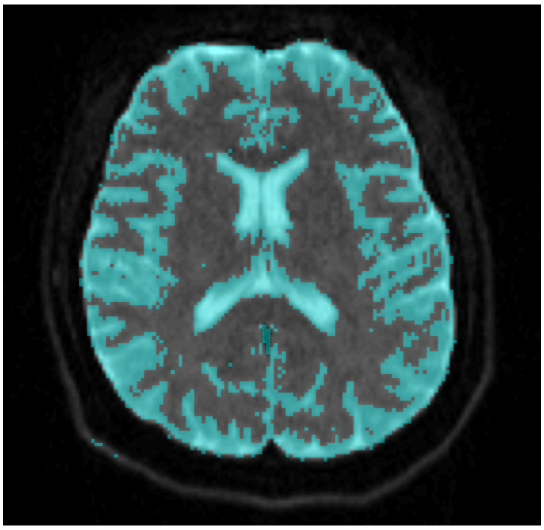

Editing Tools

These tools allow you to make changes to the existing mask

Rotations

> These tools affect all cuts

When using this tool, the mask will be mirrored horizontally or vertically in its entirety

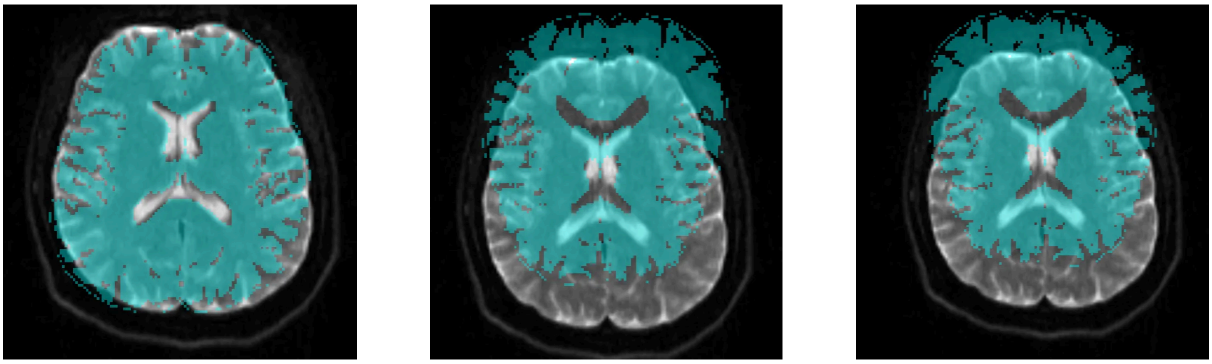

Expand | Contract

These tools expand or contract the drawn mask by one pixel; if the `Vertical` switch is turned on, they will apply their action to all cuts

> Contracting a mask that exists only in one slice using the switch will remove it because contracting a one-pixel mask

Contracting and expanding can help refine automatic masks so they select more or less area

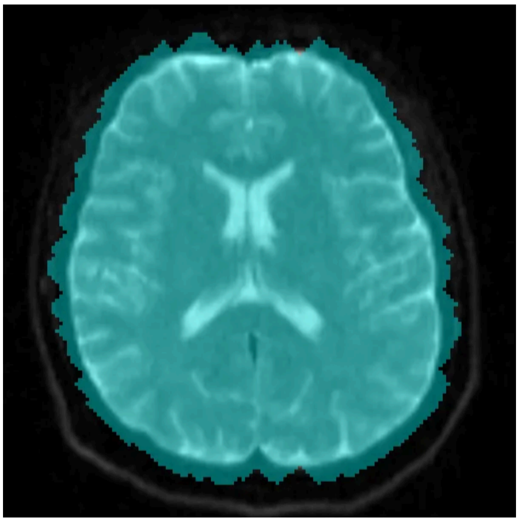

Máscara expandida 3 veces

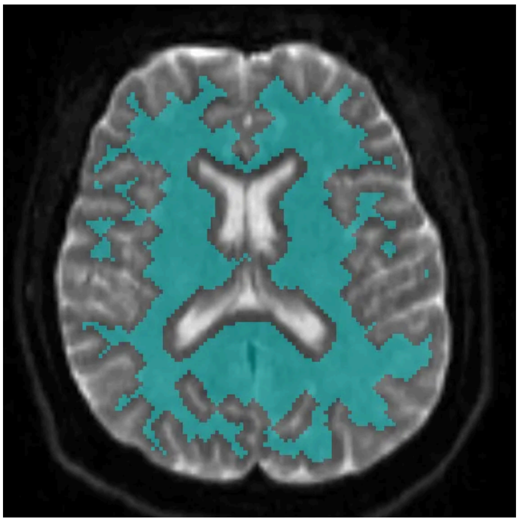

Máscara contraida 3 veces

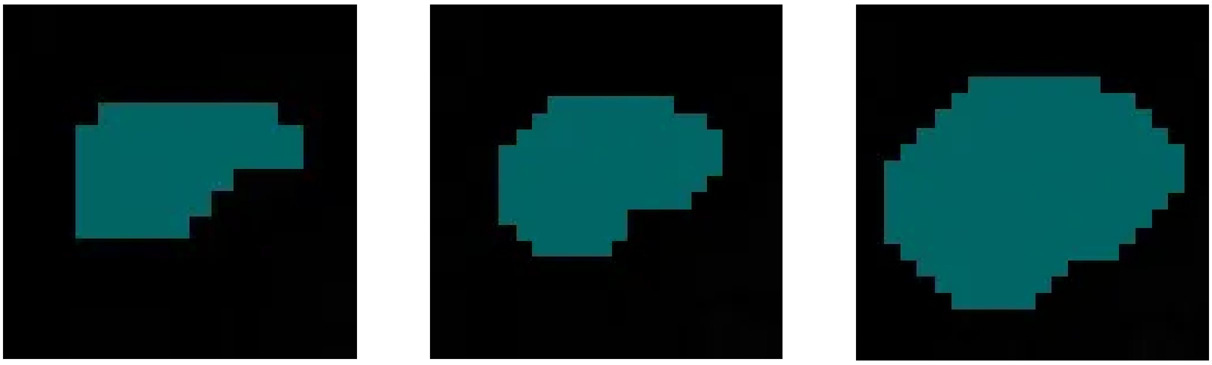

Clear

This tool removes the entire current mask, useful for starting from scratch.

Save tools

Save

Clicking this will open a window to save the selected mask in NII format to the program's output folder; you must enter a name, and it can be loaded again when using this study

Load

This opens a file dialog to load a previously saved mask in NII format.

> You cannot load a mask of a different size than the current study

Using automatic regions

> ⚠️ As with other tractography processes, knowledge of the anatomy of the target region is required, and any validation should be performed with the assistance of a trained professional

To generate tracts automatically, you can use the atlases available in the tractography tab; these replace seed masks with predefined masks corresponding to anatomical regions. These are available in the tractography tab or as an option when selecting automatic tractography in the simple interface.

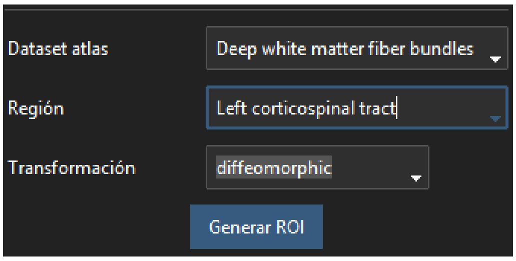

In the interface, you can select:

- The atlas to be used (see Automatic regions list); each atlas includes a T2 MRI scan that will be used to fuse with the patient's scan and assign its regions to the patient's scan as masks.
- The region or subregions to generate. The "All" option generates all those available for the current selection, whether all those in the atlas or all subregions of the current region

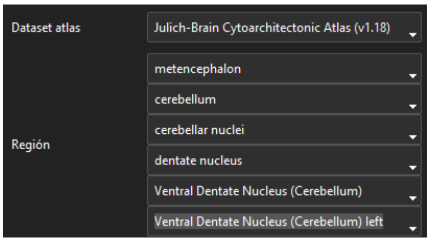

Selector de regiones

- The transformation to be applied to the atlas; these transformations determine how the atlas regions will be fused with the patient's study
    - Rigid: maintains the scale of the original atlas; not recommended since it does not ensure the location or size at all

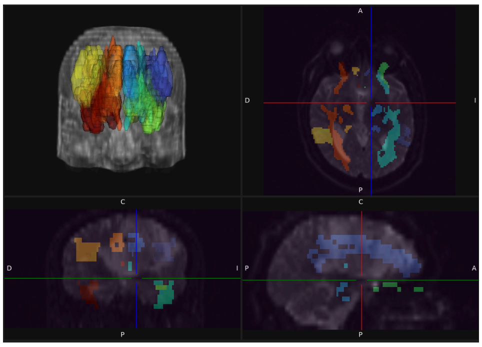

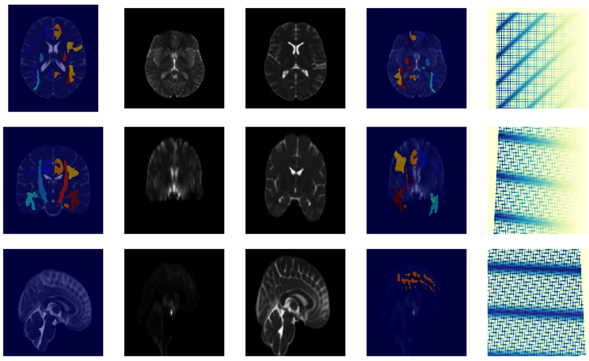

    - Affine: Merges the studies, allowing the scale of the atlas study to change to better match that of the patient.

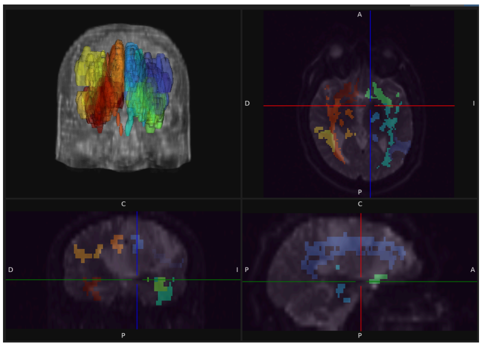

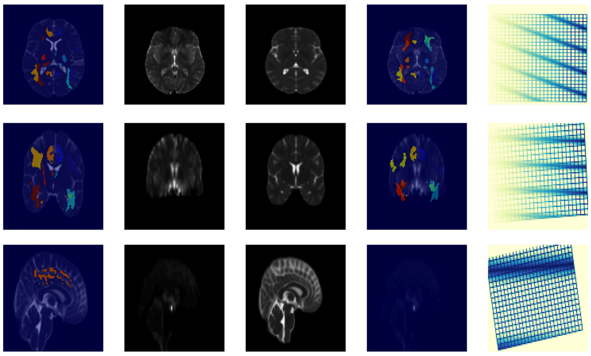

    - Deformative: Deforms the atlas study to conform to the anatomy of the patient's study; this may compromise the accuracy of the regions in patients

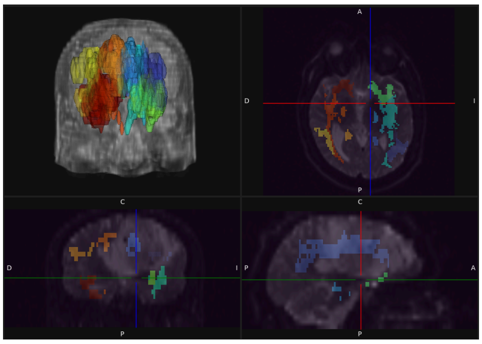

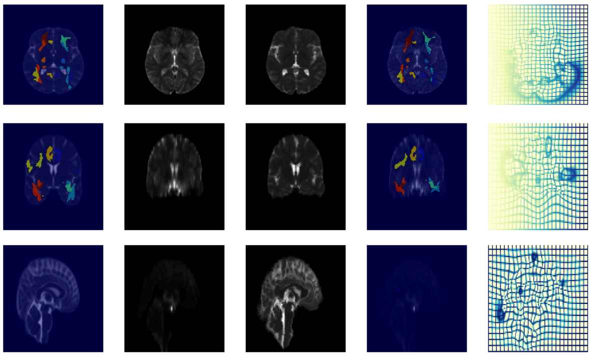

When selecting one of these options, the previous steps are performed as prerequisites, and the results can be validated using images exported to the folder `users/<user>/sm_tracto/img`

💡 The result of this fusion depends on the quality of the generated Otsu mask; it is important that it contain only the patient's brain. If noise was present in the anterior region (nose, eyes, etc.) during generation, it is best to manually edit the mask to remove these areas to ensure better results

⚠️ It may happen that when transforming the ROIs, some are lost due to their size or the transformation applied, resulting in empty regions or tracts without fibers

User tutorial (simple interface)

Below is a step-by-step explanation for generating a tractograph from 0

To perform a tractography it is necessary to generate the objects in the image in the order from top to bottom, the simple interface is responsible for grouping the creation of these objects in fewer steps.

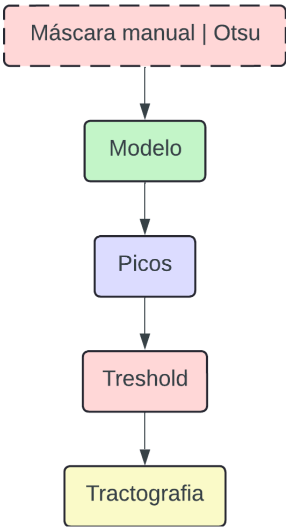

💡 When you open the program from the NVML Planner, the output folder is selected automatically. If you open it otherwise, you must select the patient folder in the catalog

1. Load the resonance data

The application allows you to upload the DICOM files of the diffusion resonance or the processing files for tractography (NIFTI, BVALS, BVECTS) one by one, or if all three are in the same folder, open them in a single search.

📢 To maintain compatibility with the Planner/Navigation software you must import from DICOM at least the first time, if opened from files for tractography they cannot be imported into a schedule and displayed in the navigation.

1.1. Upload the DICOM files

By clicking the button `Open from DICOM` A selector will open to open from a folder with DICOM files, when selected the necessary conversions will be made and exported to the selected or automatically placed output folder.

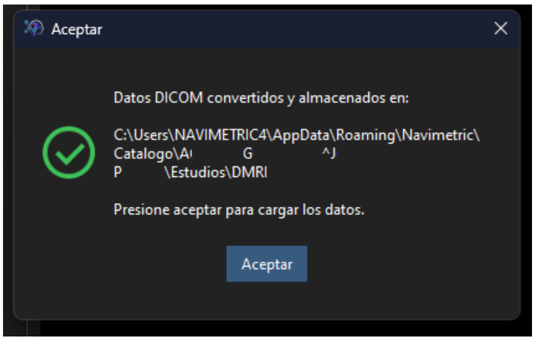

DICOM file conversion confirmation

1.2. Load the data one by one

To open one by one we are going to click one by one on the open buttons in the top panel of the window

Clicking will show us a window to select a .nii or .nii.gz file, an MRI file must be selected. Then we will click on the buttons to load the B values and B vectors and select their respective files.

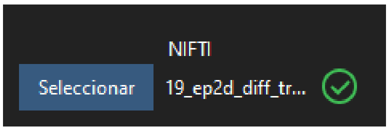

Button to load the Nifti data

if the three selected files are valid, the program will inform us, if not, it will show us an error window with the reason.

1.3. Upload data from a folder

On the other hand, if we have the three resonance files processed in the same folder, we can click on the button `Open from folder from` the top panel and select the folder where we have the three files.

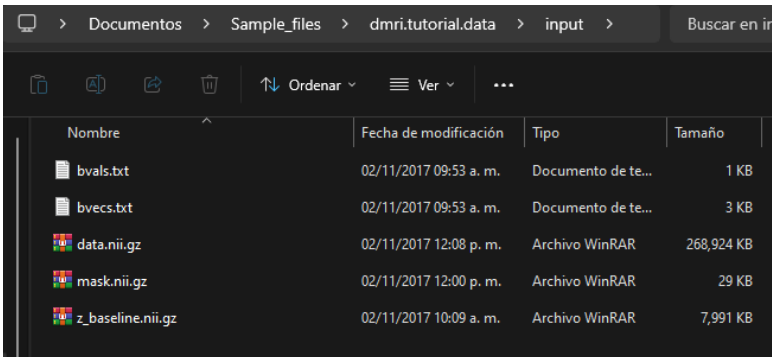

Ejemplo de carpeta con los tres archivos en ella

Once this is done, the program will go through the folder looking for the files and inform us of the result with a message. If the search fails, any of the files can be replaced individually to continue with the process.

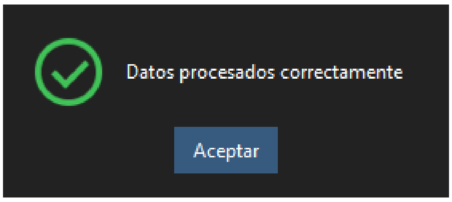

Ventana de confirmación de carga exitosa

Once the file is loaded we can change the display color and orientation in the image tab of the left panel

> 💡 After opening an image we can use the arrow keys on the keyboard (⬅️⬆️⬇️➡️) to change the current cut and image. And the keys `z` , `x` and `c` to change axis.

2. Generate model and peaks

With the data loaded we are going to select the model we want to train in the list `Model from` the section `2. Generate the reconstruction` model and then we will click on the generate button to start the process, once it finishes generating the otsu mask, the selected model and the peaks generated by that model will have been created.

For this tutorial we are going to train a CSA model to show tract cleanliness in the planner.

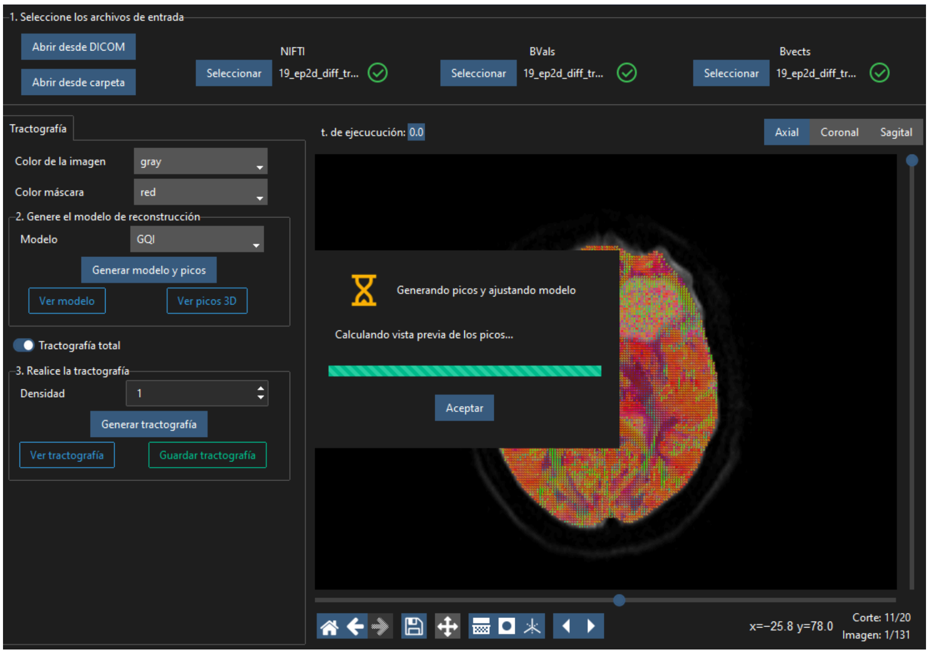

Interfaz al terminar de generar los objetos en el paso 2

Press the button `See model` take the current cut from the right panel and start the 3D view of the result, so you can review the results at specific points if you want.

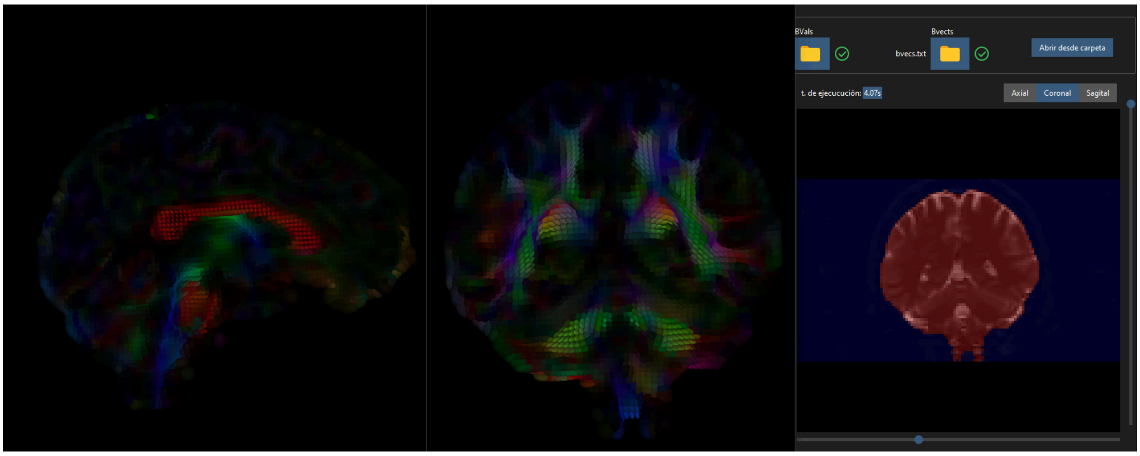

Vistas previas de un modelo de tensor entrenado

Below the generate button you can open a view of the peaks of the entire studio using the button S `ee 3D` peaks , the latter may take longer in high-quality images

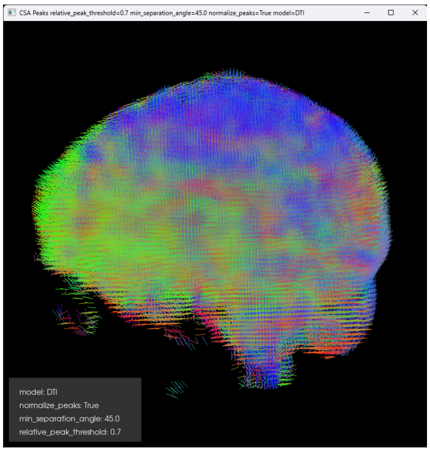

Vista completa de picos generados

> 💡 In 3D view you can rotate with left click, move with center click and zoom with right click or mouse wheel

2.1. Choose type of tractography

Below section 2 there is a switch to choose whether you want to do a total or seed tractography, changing its status will show different options in the interface.

2.1.1 Draw seed for tractography

Disabling the switch will unfold the section `2.1.` to draw the seed for tractography

We are going to descend on the axial axis and with the size 2 brush we are going to paint as seen in the image below, guiding ourselves with the peaks.

(See Mask Editing Tools)

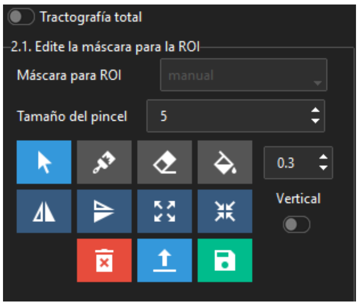

Panel de edición de máscara

the small region corresponds to the speech zone and will be our seed of tractography

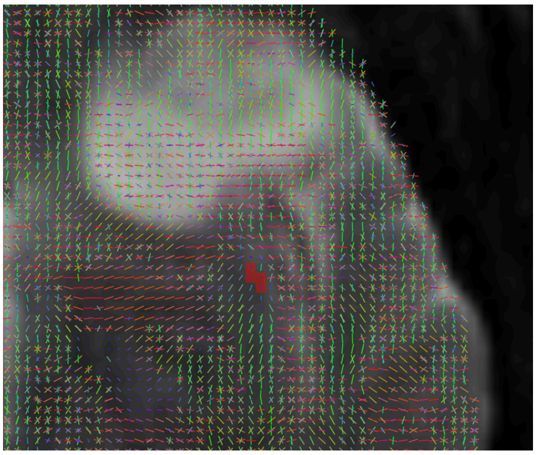

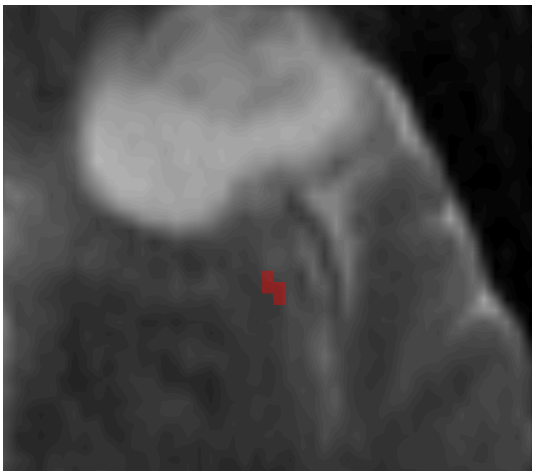

▼ **Another easy tract to see and generate**

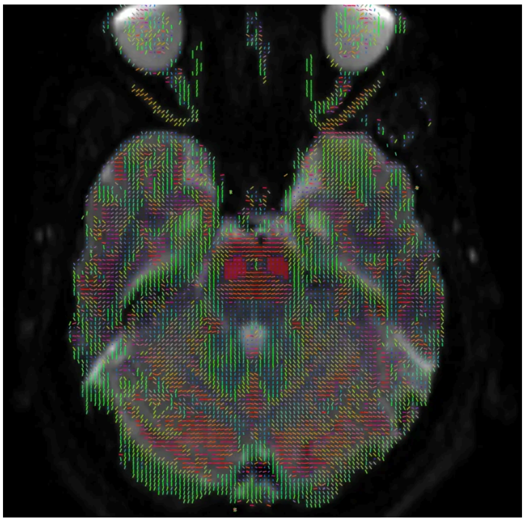

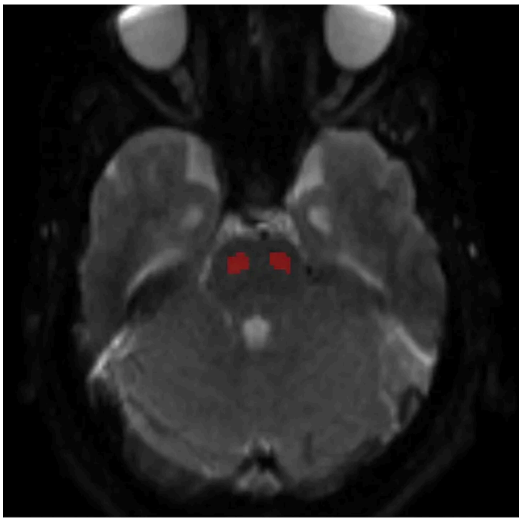

💡 To draw the mask with the peaks activated it is useful to change the `Color` from the mask at the top of the left panel to one that matches the tone with the peaks you are looking to select

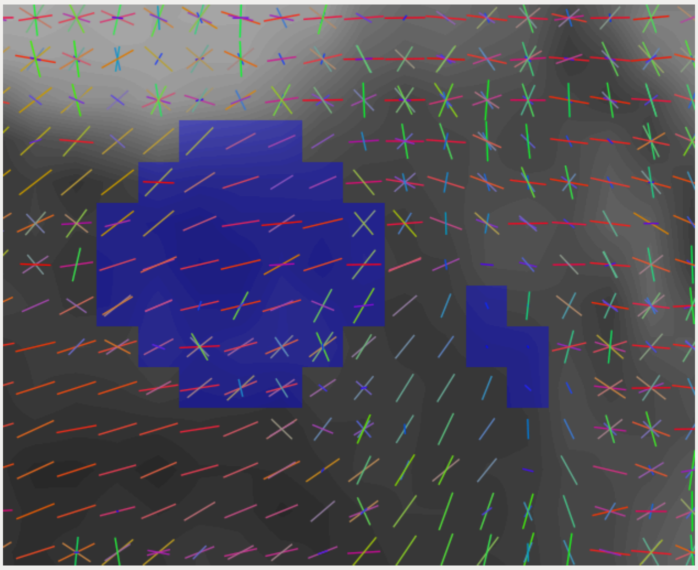

Los picos que no son azules resaltan y nos muestran que hace falta deseleccionar la zona o limpiar los tractos luego de generarlos

3. Generate tractography

In the section `3` we are going to generate a tractography from the point we draw, we will click on `Generate` and in `See tractography`

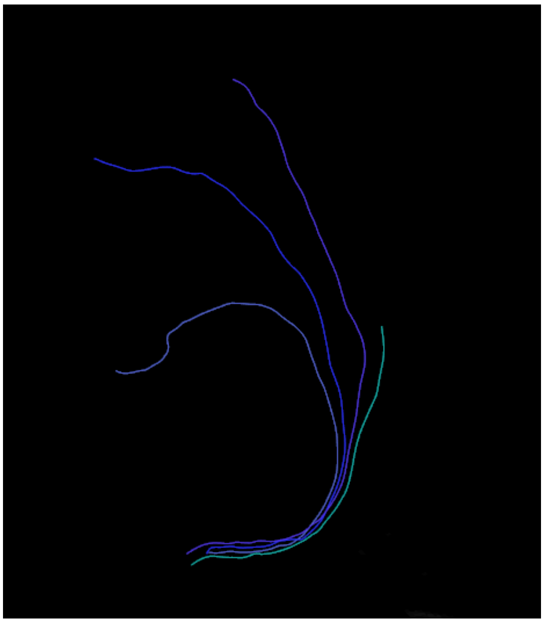

Since our seed is small, we can generate a new tractography with greater density to see the region in more detail, so we are going to raise the density to 4 and press again `generate` and in `show`

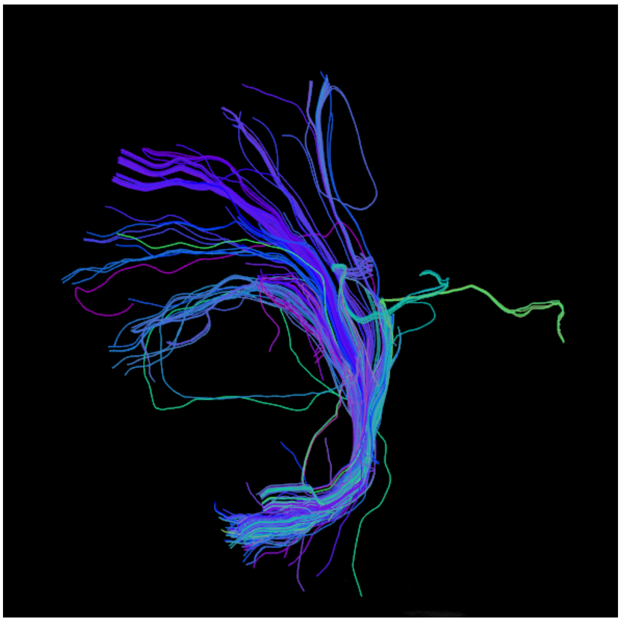

This tractography is now ready to be exported and used in the main module, to press the button Save `tractograph`y and a name is given to it simple_tutorial, this will save the tracts to the patient folder for import into the scheduler.

3.1. Group the tracts to clean them

Below the active tract selector is the select number of target groups, in this we are going to put `2` and we press on `Group` , This will generate two subgroups of tracts that we can see if we select them in the list generated `tracts` (the name is the name of the tracts generated with _N at the end)

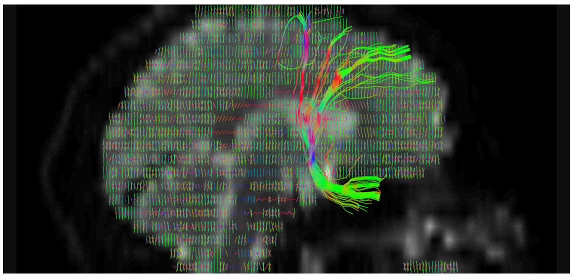

Grupo de tractos 1

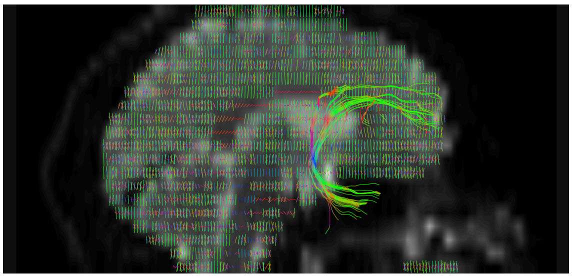

Grupo de tractos 2

Additionally we can save these to add to the planner.

4. Load the tractography into the scheduler

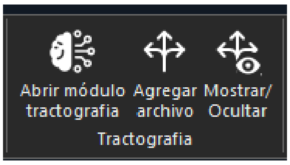

After generating all the tracts that you want to see in the navigation, we return to the planner and press `Add file in` the top bar, this will open a window to select the files needed to merge.

4.1 Files generated by the program

First we will select the file .vtk that we generate and press accept.

The second parameter, `the dMR` file folder it must be correctly selected automatically if the process was carried out starting in the scheduler. If not, the output folder selected in the tractography module must be selected.

For `volume selection` we select RM, as it will give better results and press next.

4.2. Fuse diffusion resonance with a planning study

When the next page loads, we will use the sliders to position the two surfaces as close together as possible.

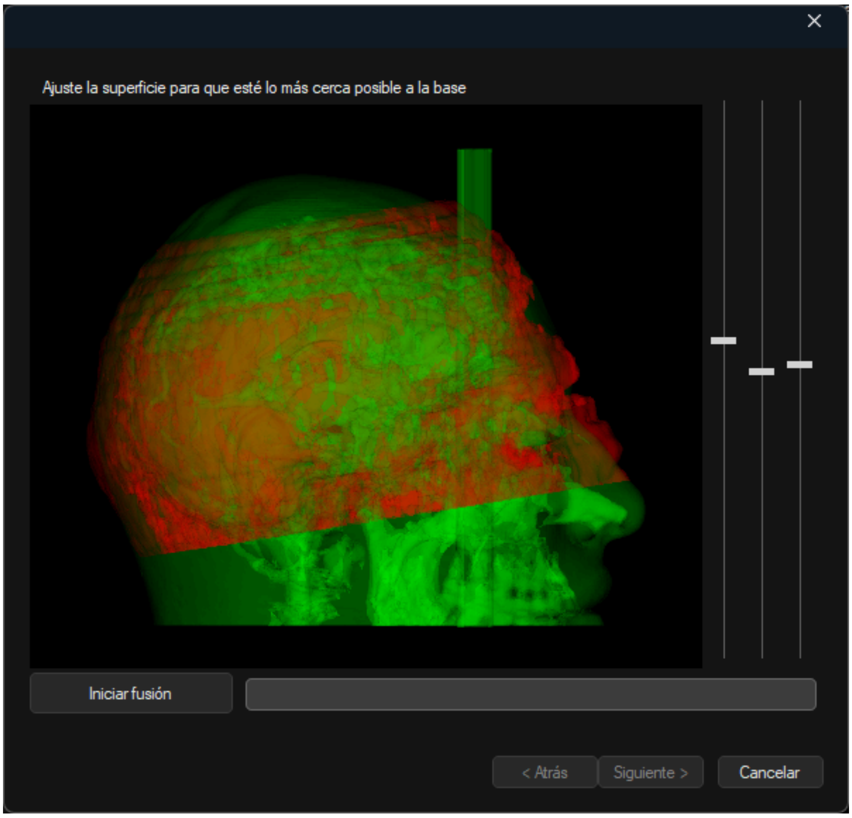

💡 For this process it is useful to look for common reference points on both surfaces such as the nose. the shape of the brain or skull and forehead. You can also move the 3d view to check from more than one angle.

Once the surface is located, we press St`art fusion` and we will wait for it to finish to give next.

4.3. Select the color of the tracts and locate them in the planner

On the next page, we will select the color with which we want to see the tracts in the planning/navigation software (if we want to repaint them), press `Convert` and we wait for the transformation to finish to give next, the transformation will locate the tracts with the fusion of the previous page and if it is a secondary study it will adjust the position with the fusion of studies done in the planner.

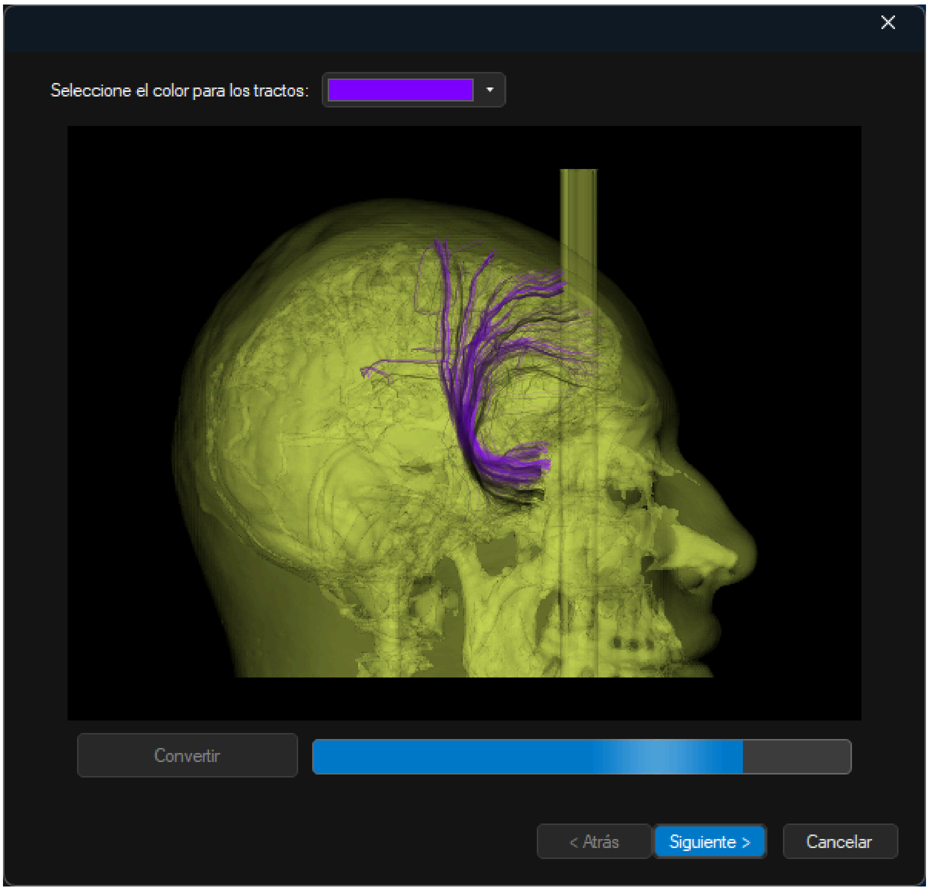

4.4. Clean the tracts

On the next page you can clean the tracts to eliminate fibers that we do not see necessary, to eliminate we will hover the mouse over them by pressing right click, alternating between turning the camera and deleting with the switch at the top of the dialog.

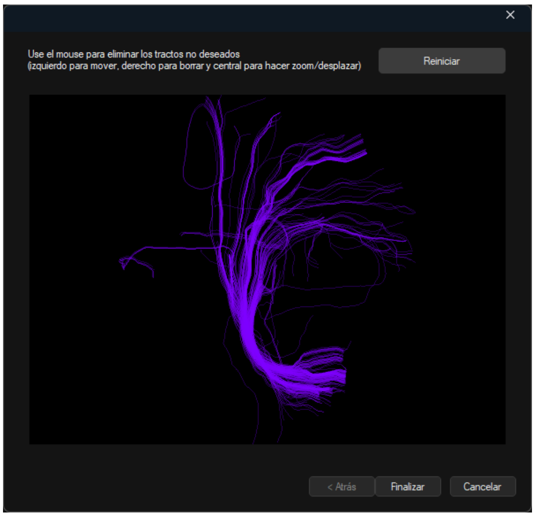

Antes de la limpieza

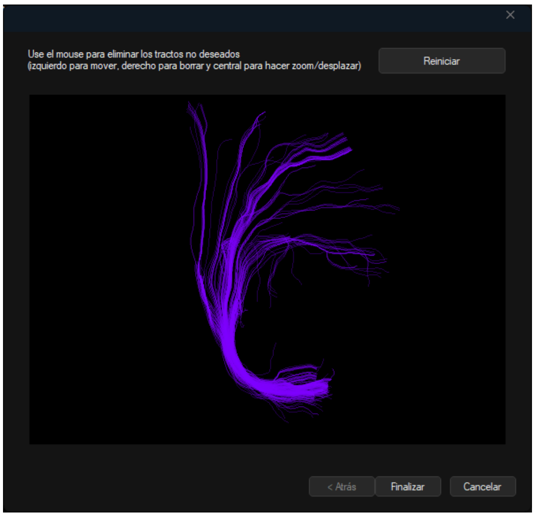

Después de la limpieza

When we finish cleaning we can click on `finish` and review the result in the planner

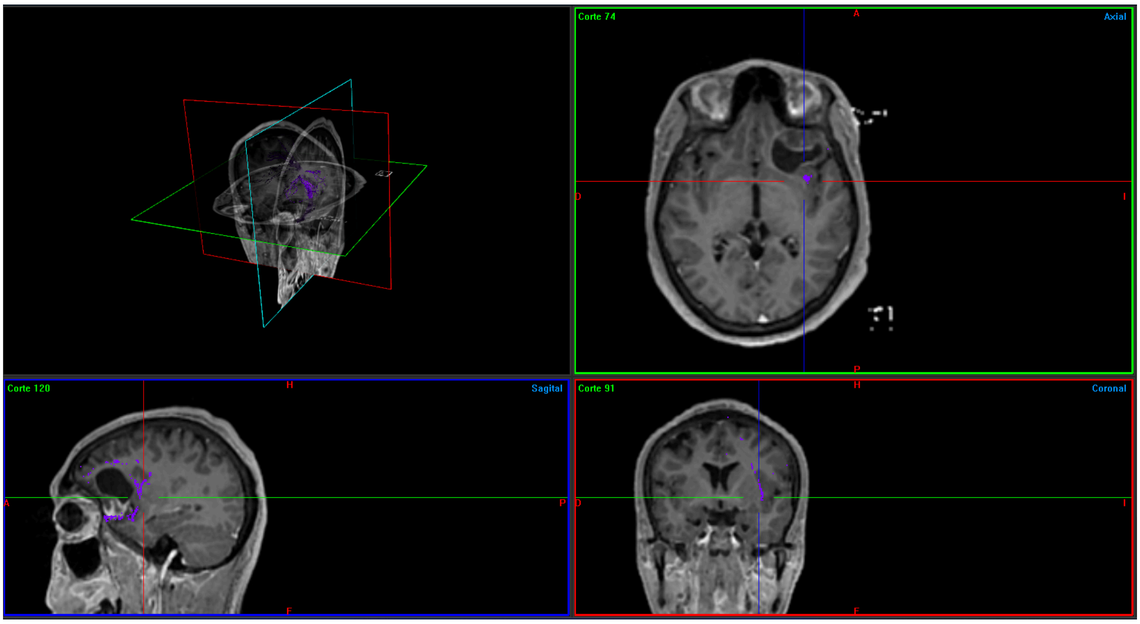

User tutorial (advanced interface)

Below is a step-by-step guide to generating a tractography from scratch

To perform a tractography, you need to generate the image objects in top-to-bottom order; the first mask is optional but recommended to speed up the following steps.

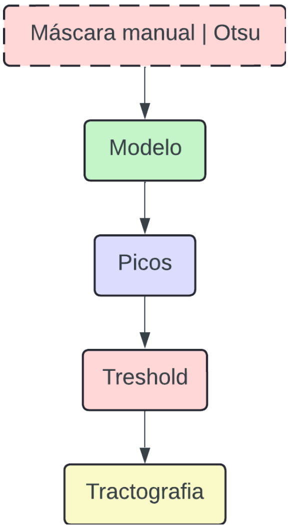

> 💡 When you open the program from the NVML Planner, the output folder is selected automatically; if you open it another way, you must select the patient's folder in the catalog

1. Load the MRI data

The application allows you to load the DICOM files from the diffusion MRI or the processing files for tractography (NIFTI, BVALS, BVECTS) one by one, or if all three are in the same folder, open them in a single search.

> 📢 To maintain compatibility with the Planner/Navigator, you must import from DICOM at least the first time; if you open from tractography files, they cannot be imported into a planning session and displayed in navigation.

1.1. Loading DICOM Files

Clicking the `Open from DICOM` button will open a file browser to select a folder containing DICOM files. Once selected, the necessary conversions will be performed, and the files will be exported to the selected output folder or the default folder.

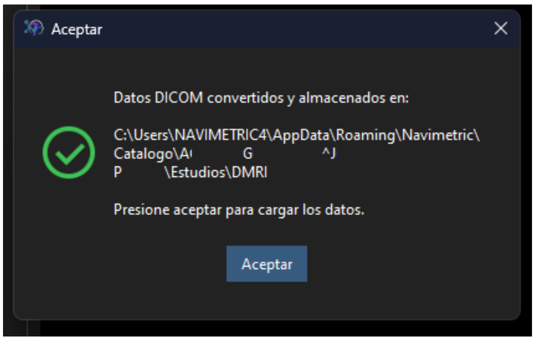

Confirmación de conversión de archivos DICOM

1.2. Loading data one by one

To open files one by one, click the Open buttons in the top panel of the window one at a time

Clicking will display a window to select a .nii or .nii.gz file; you must select an MRI file. Then click the buttons to load the B values and B vectors and select their respective files.

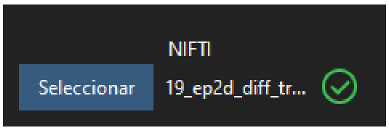

Botón para cargar el archivo NIFTI

If the three selected files are valid, the program will inform you; if not, it will display an error window with the reason.

1.3. Loading data from a folder

Alternatively, if the three processed MRI files are in the same folder, you can click the `Open from Folder` button on the top panel and select the folder containing the three files.

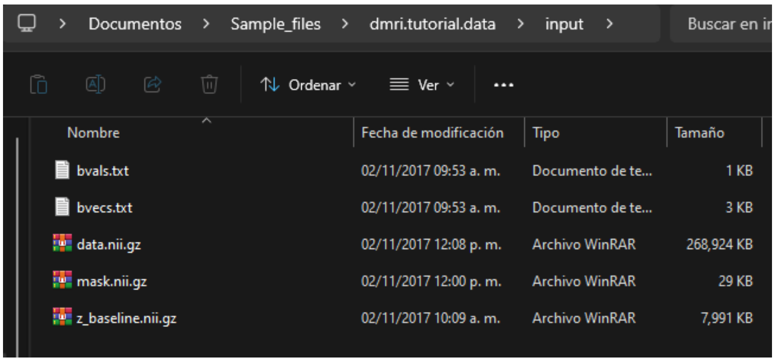

Ejemplo de carpeta con los tres archivos en ella

Once this is done, the program will scan the folder for the files and display a message with the result. If the search fails, you can replace any of the files individually to continue the process.

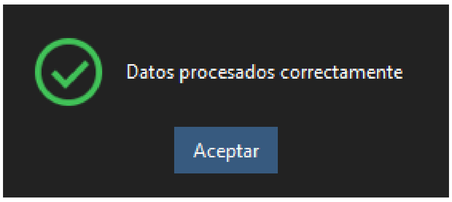

Ventana de confirmación de carga exitosa

Once the file is loaded, you can change the display color and orientation in the Image tab of the left panel

> 💡 After opening an image, you can use the arrow keys (⬅️⬆️⬇️➡️) to change the current crop and view. Or press `Ctrl/Shift` and scroll the mouse wheel 🖱️

2. Generate a mask

Clicking the `Mask` tab or pressing `Ctrl+2` displays the mask options. To generate the initial mask, you can draw it using the tools or generate a median Otsu mask.

> 📢 When opening a new MRI image, an empty `manual` mask is automatically created. To select it, you must change the `Active Mask` option by clicking or using the mouse wheel.

2.1. Drawing the manual mask

To draw the manual mask, you can use the brush from the mask tools section and draw the brain layer by layer, or you can use the fill tool and select the area you want to fill, making sure to adjust the tolerance value to the right of the tool button so that it selects more or less depending on where you click.

(See Tools for Mask Editing)

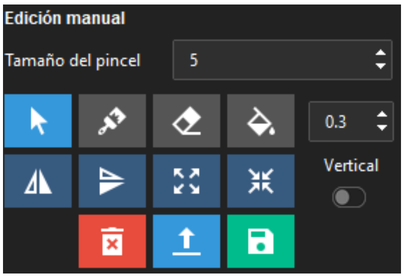

Panel de edición de máscara

2.2. Generate Otsu Mask

Below the manual mask editing section is the Otsu median mask section, where we can edit the three parameters explained in detail on {·} Objetos y conceptos clave to generate a mask. When you click the Generate button, the mask will be calculated based on the image and added to the list of possible masks for `Active Mask`

It is important to verify that the generated mask meets the image's requirements, as a very high radius or a very low number of passes can cause regions necessary for mapping to be lost.

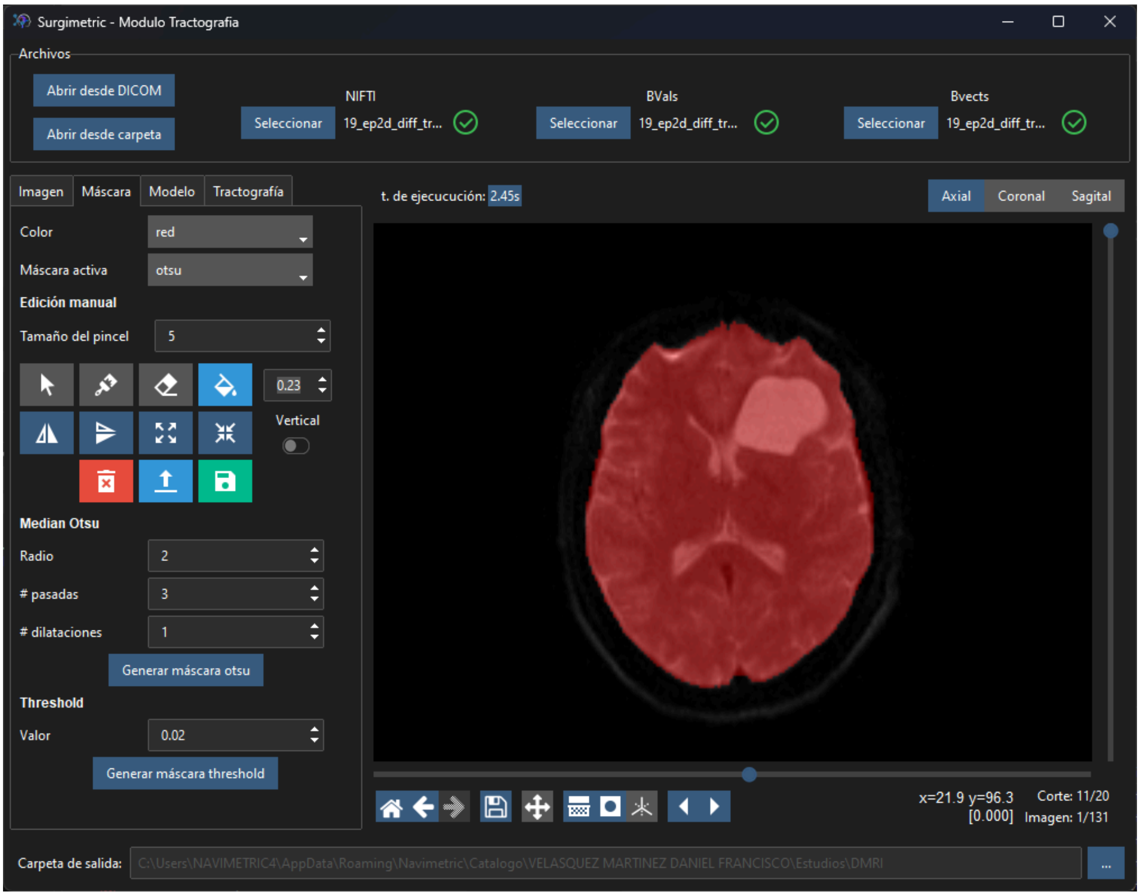

Resultado de generar máscara otsu y rellenar espacios en blanco con la herramienta fill

💡 Generated or uploaded masks can also be edited with the manual tools to make them more precise

3. Train a model

With the generated mask, you can go to the `Model` tab to generate the model you will use. In the list of models, there is a button to generate the model and one to display the result in the current view section; all models automatically use the active mask.

Models are independent and remain stored for future steps, so it is possible to train them all and use them as desired for comparison.

Opening the preview of any model uses the current cross-section from the right panel, so you can review specific points of the model if desired

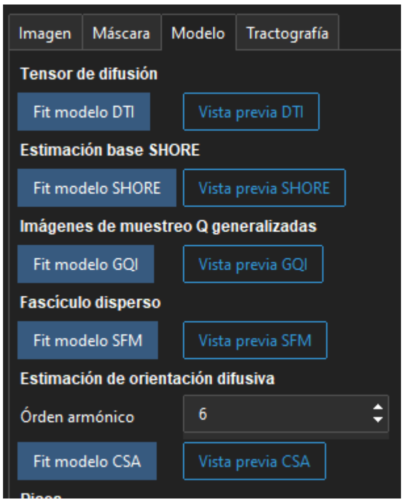

Sección de modelos

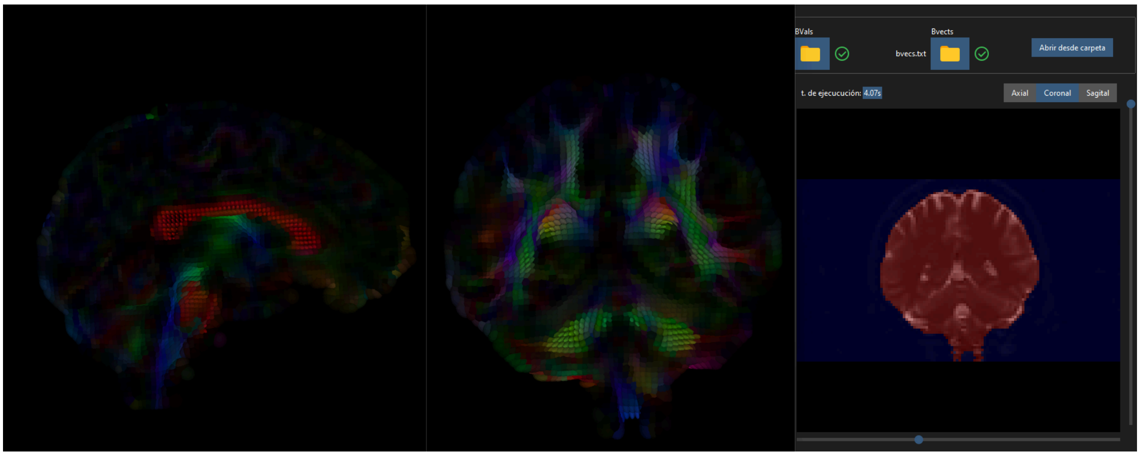

Vistas previas de un modelo de tensor entrenado

4. Generate Peaks

Below the models section are the peak generation options. Here, we will select the model we trained and generate the peaks. Once generation is complete, a preview of the current cross-section will be displayed over the image, and the peaks can be viewed in both 2D and 3D views; the 3D view may take longer to render for high-quality images

> 💡 In the 3D view, you can rotate by left-clicking, pan by middle-clicking, and zoom by right-clicking or using the mouse wheel

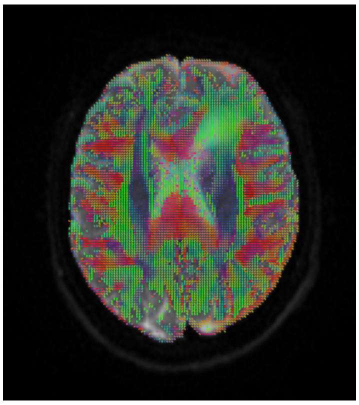

Vista previa de lo picos en un corte

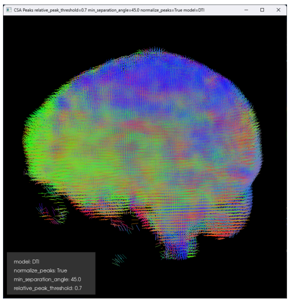

Vista completa de picos generados

By generating peaks with different parameters, you can compare their effects by switching between them and find the appropriate values for the current study.

> 💡 The 3D view can be useful for viewing the edges of the mask and adjusting the parameters or the mask to cut out noisy areas or gray matter

5. Generate a threshold mask

Once the peaks have been generated, you can generate this mask to use as a stop criterion for tractography. To do this, return to the `Mask` tab and, in the Threshold section, adjust `the Value` until you have a mask that filters as desired. Like the masks seen in step (2), the threshold mask can also be edited with the manual tools.

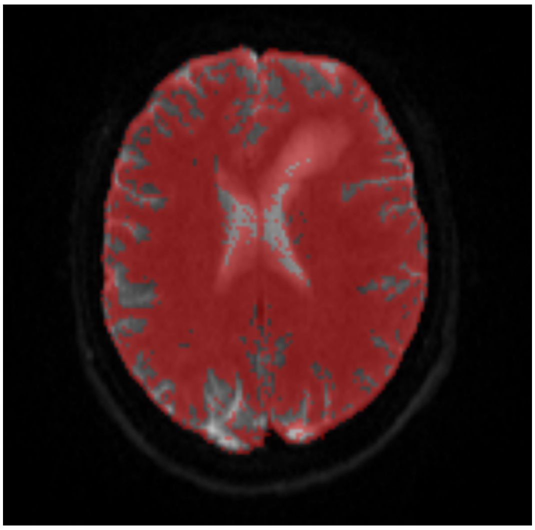

5.1. Draw a seed mask

To generate a tractography of a specific region of interest, you need to draw the seed as a mask; therefore, at this point, you can edit a mask to serve this purpose. In this example, we will use the manual mask after selecting it.

We will move down the axial axis and, using the size 2 brush, paint as shown in the image below, guided by the peaks.

The small region corresponds to the speech area and will be our tractography seed

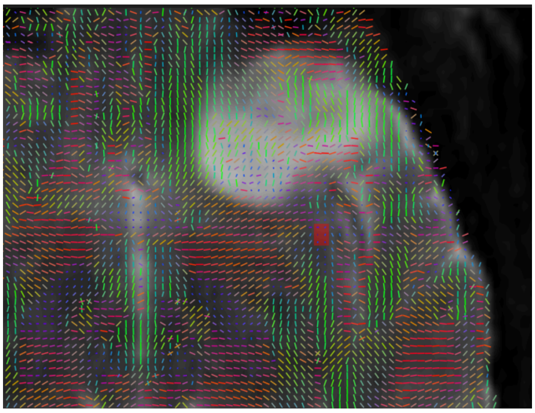

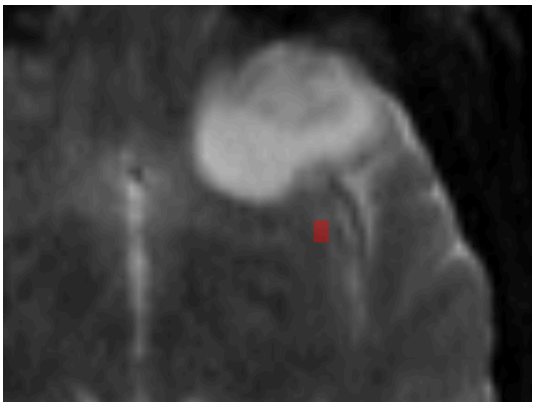

▼ **Another tract that is easy to see and generate**

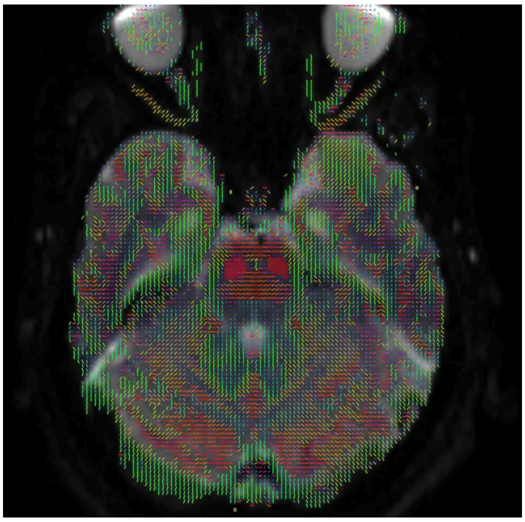

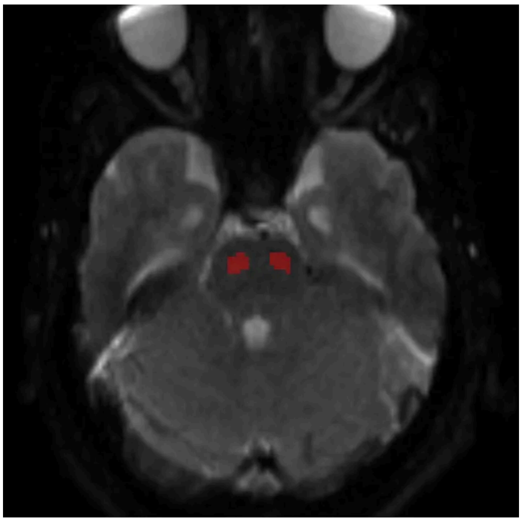

💡 To draw the mask with the peaks active, it is helpful to change the mask `color` to one that matches the tone of the peaks you want to select

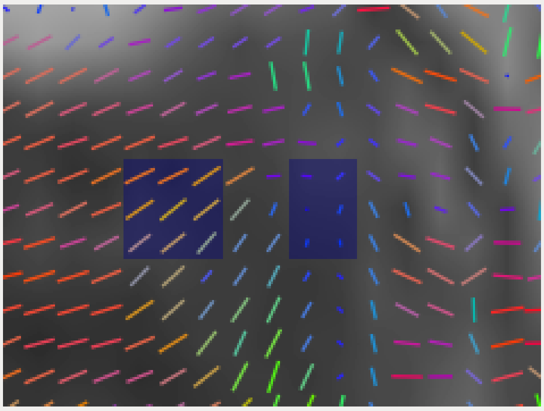

Los picos que no son azules resaltan y nos muestran que hace falta deseleccionar la zona o limpiar los tractos luego de generarlos

6. Generate a tractogram

In the `Tractography` tab, we will select the manual mask we just edited to generate a tractography from that point, then click `Generate` and `Show`

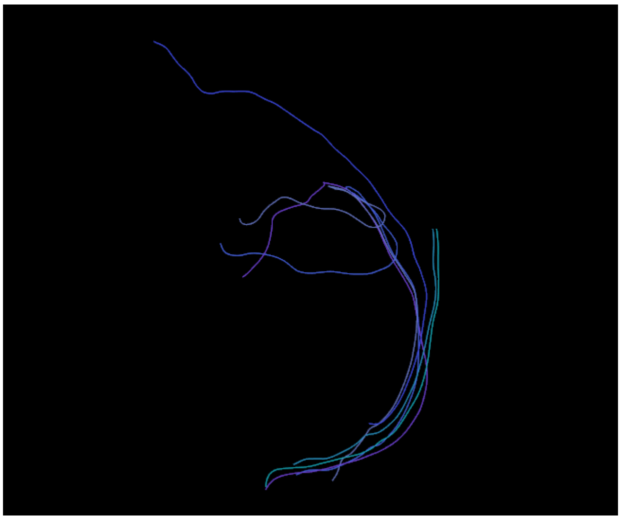

Since our seed is small, we can generate a new tractography with higher density to view the region in greater detail, so we will increase the density to 4 and click `Generate` and `Show` again

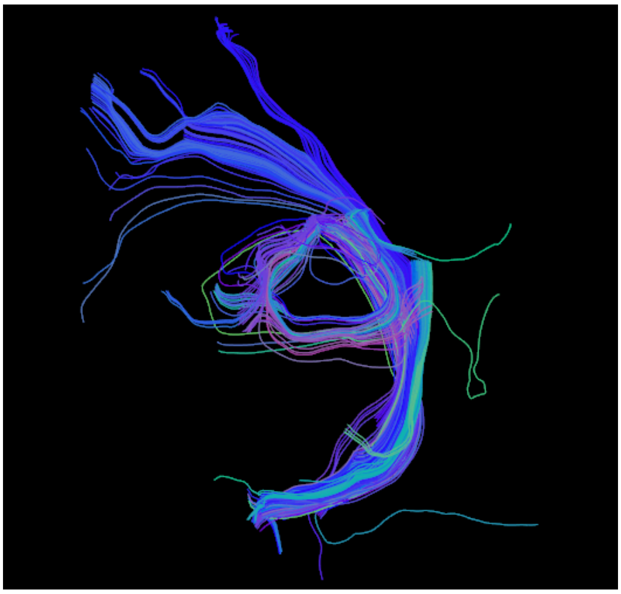

This tractography is now ready to be exported and used in the main module. To do this, click the `"Save Tractography"` button and name it "tutorial"; this will save the tracts in the patient's folder so they can be imported into the planner.

6.1. Grouping the tracts to clean them up

Below the active tracts selector is the target group number selector. Here, we'll set it to `2` and click `"Group`." This will generate two subgroups of tracts, which we can see by selecting them in the list of `generated tracts` (the name is the name of the generated tracts with _# at the end).

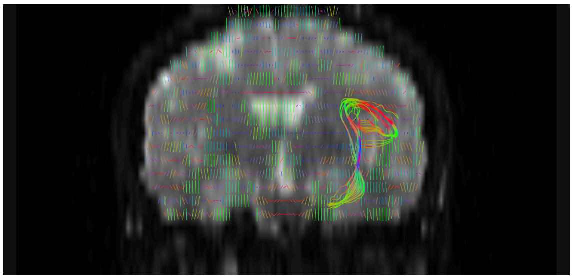

Grupo de tractos 1

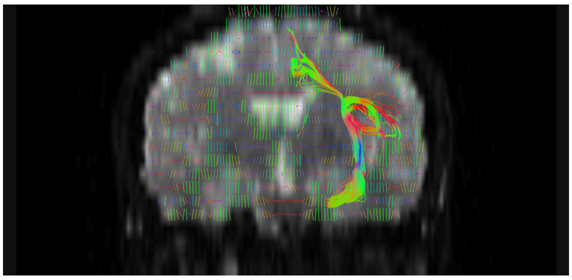

Grupo de tractos 2

Additionally, we can save these to add them to the scheduler.

7. Load the tractography into the planner

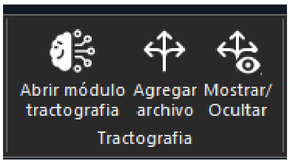

After generating all the routes you want to see in the navigation, return to the planner and click `"Add File` " on the top bar; this will open a window to select the files needed for merging.

7.1 Files generated by the program

First, select the .vtk file you generated and click OK.

The second parameter, the `dMR File Folder`, should be automatically selected correctly if the process was started in the planner; if not, you must select the output folder chosen in the tractography module.

When `selecting the volume` for fusion, choose MRI, as this will yield better results, and click Next.

7.2. Merge the diffusion MRI with a planning study

When the next page loads, use the sliders to align the two surfaces as closely as possible.

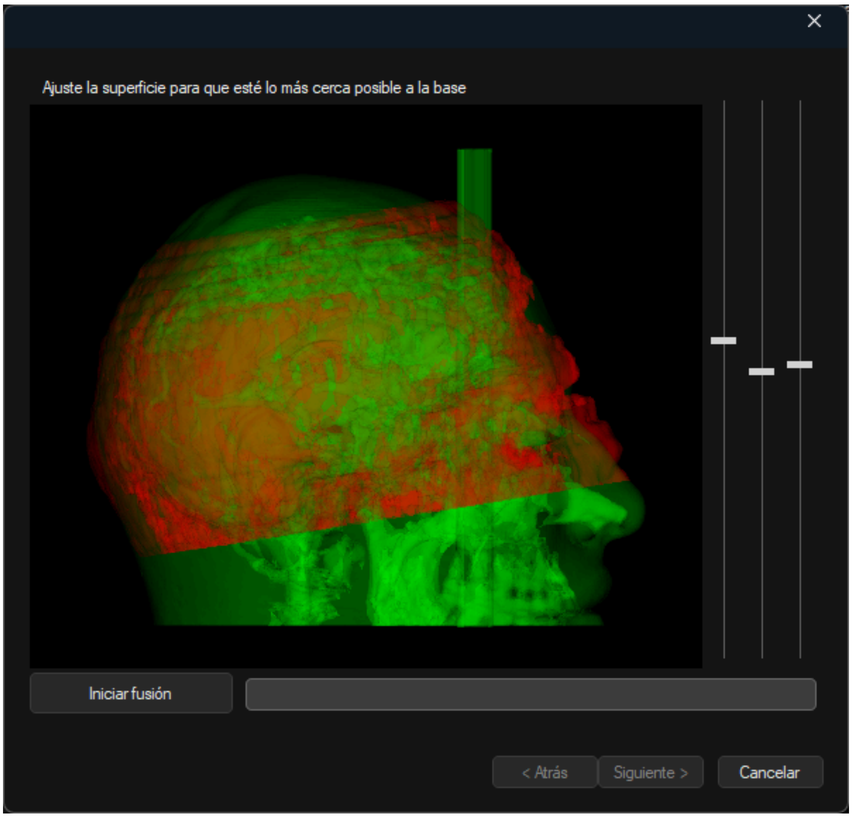

💡 For this process, it is helpful to look for common reference points on both surfaces, such as the nose, the shape of the brain or skull, and the forehead. You can also move the 3D view to verify the alignment from multiple angles.

Once the surface is aligned, click `Start Merge` and wait for it to finish before clicking Next.

7.3. Select the color of the tracts and place them in the planner

On the next page, select the color you want to use to view the tracts in the planner/navigator (if you want to repaint them), click `Convert`, and wait for the transformation to finish before clicking Next. The transformation will align the tracts with the fusion from the previous page, and if this is a secondary study, it will adjust the position based on the study fusion performed in the navigator.

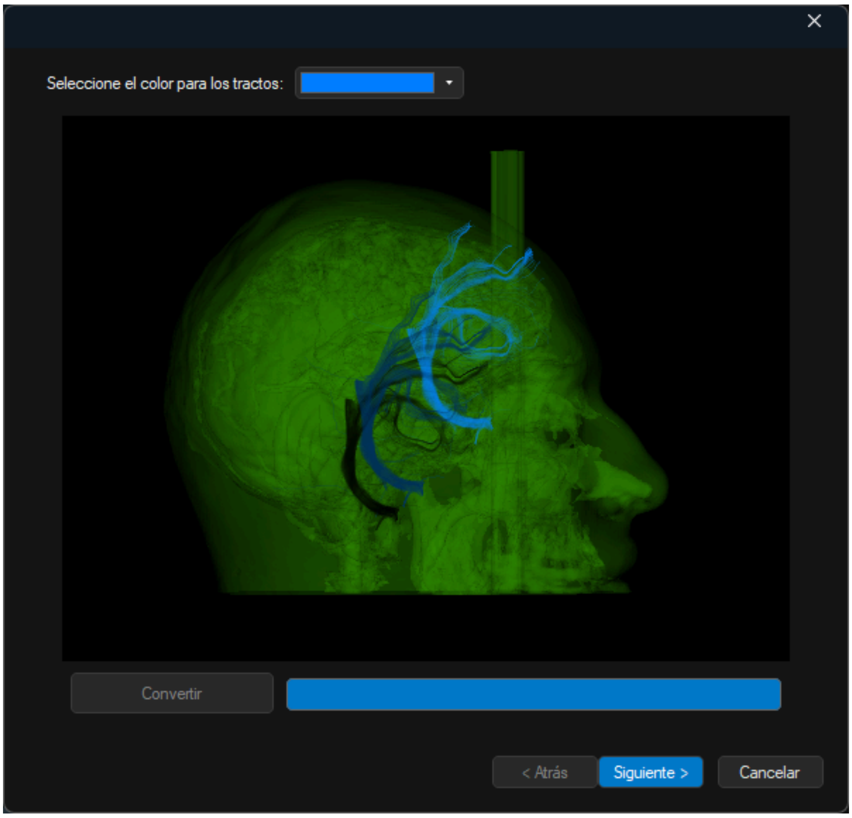

7.4. Clean up the tracts

On the next page, you can clean up the tracts to remove fibers you don't need. To remove them, hover over them and right-click, alternating between rotating the camera and deleting using the toggle at the top of the dialog.

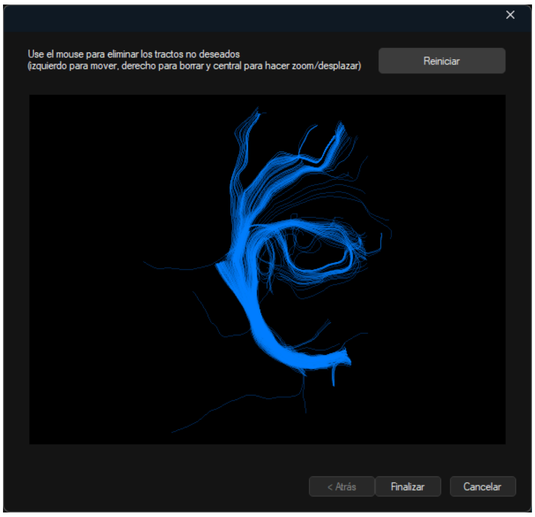

Antes de la limpieza

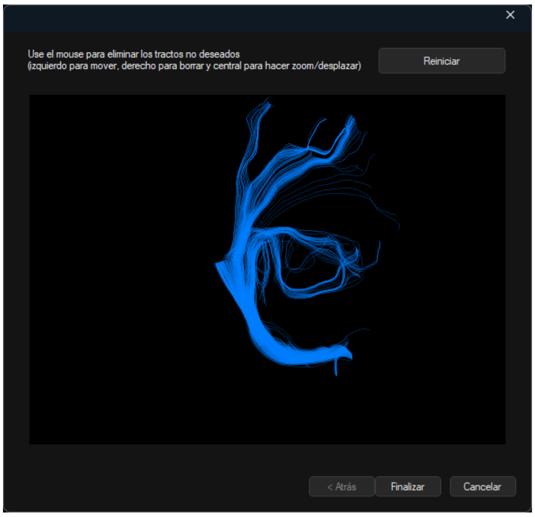

Después de la limpieza

⚠️ Cleaning should be done carefully; although some fibers may seem out of place, keep in mind that every patient's anatomy can be different, especially if there is a pathology that alters it

Once cleaning is complete, click `Finish` and review the result in the planner

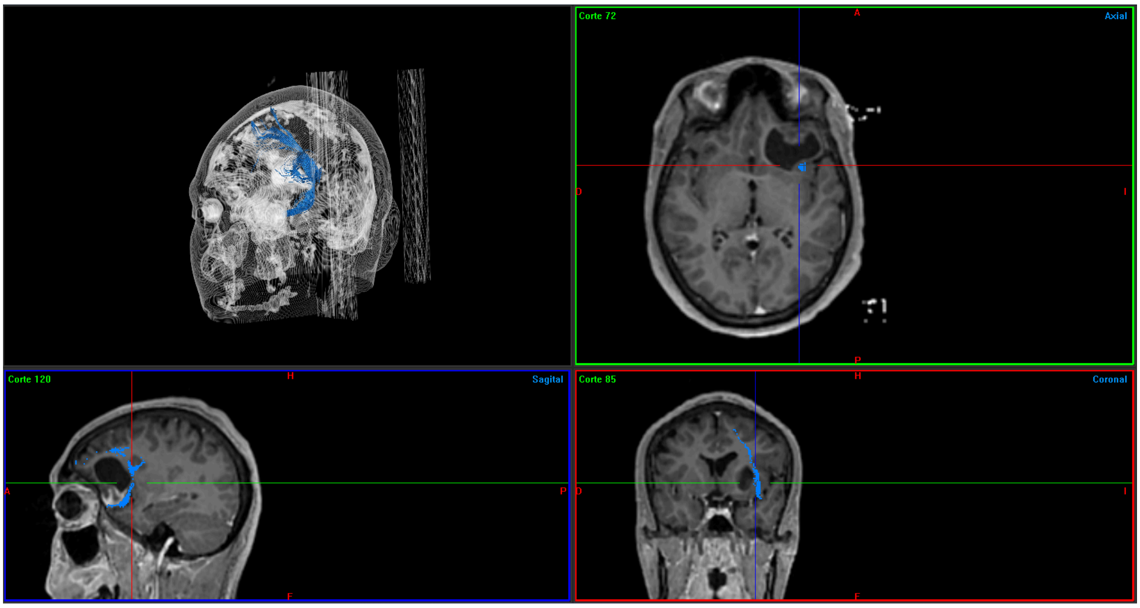

List of automatic regions

Automatic generation includes several datasets with predefined brain regions. Below is a list of all datasets and an image showing the regions. To access the complete list of region names, consult the online manual or view them within the application:

Deep white matter fiber bundles

DiFuMo Atlas (64 dimensions) (v1)

DiFuMo Atlas (64 dimensions) (v1)

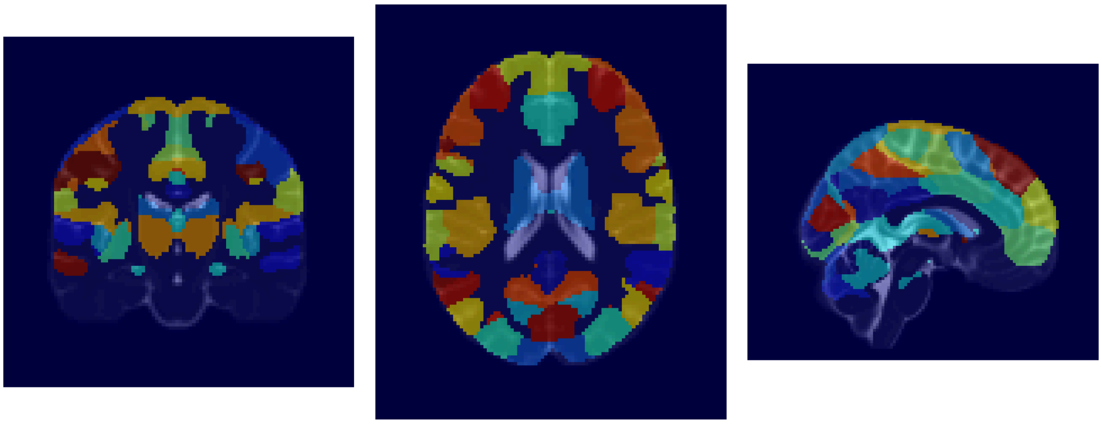

DiFuMo Atlas (128 dimensions) (v1)

DiFuMo Atlas (128 dimensions) (v1)

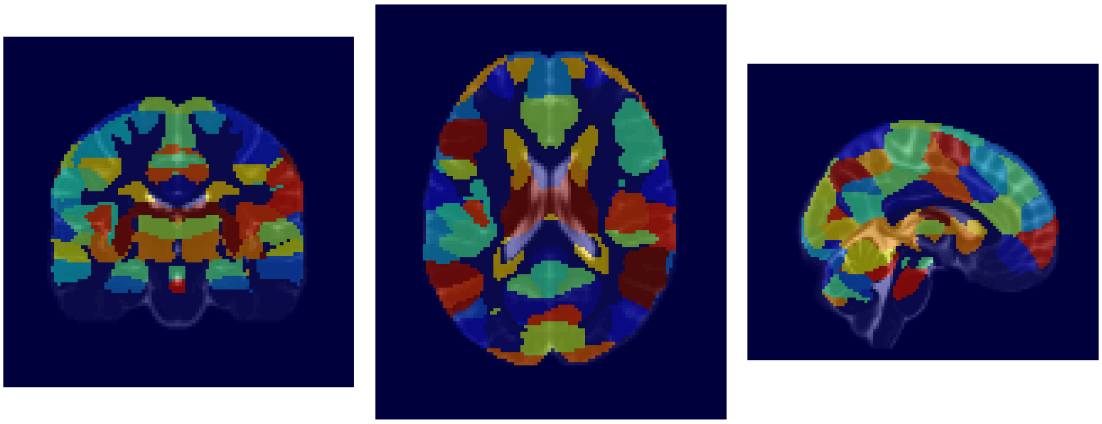

DiFuMo Atlas (256 dimensions) (v1)

-

Stanford HARDI

-

AAL Atlas

-

Destrieux 2009

-

Harvard-Oxford

Harvard-Oxford

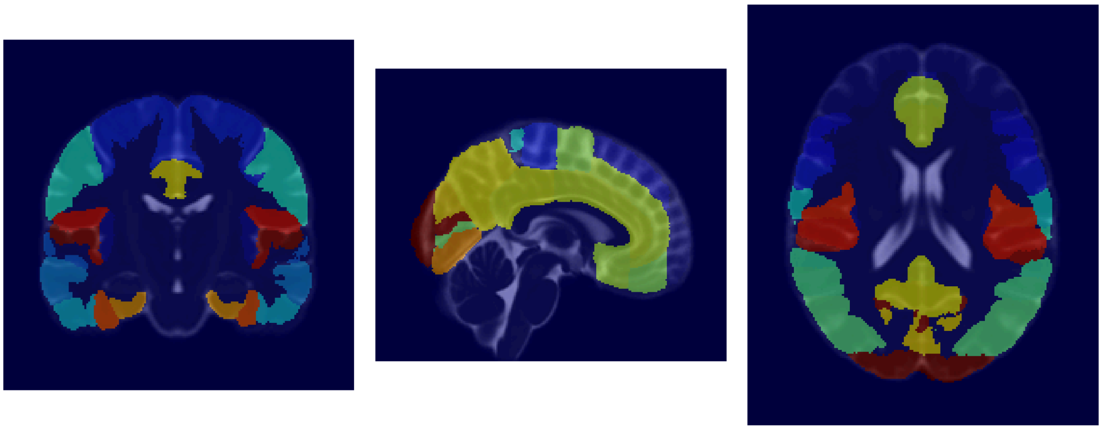

Jülich

Juelich

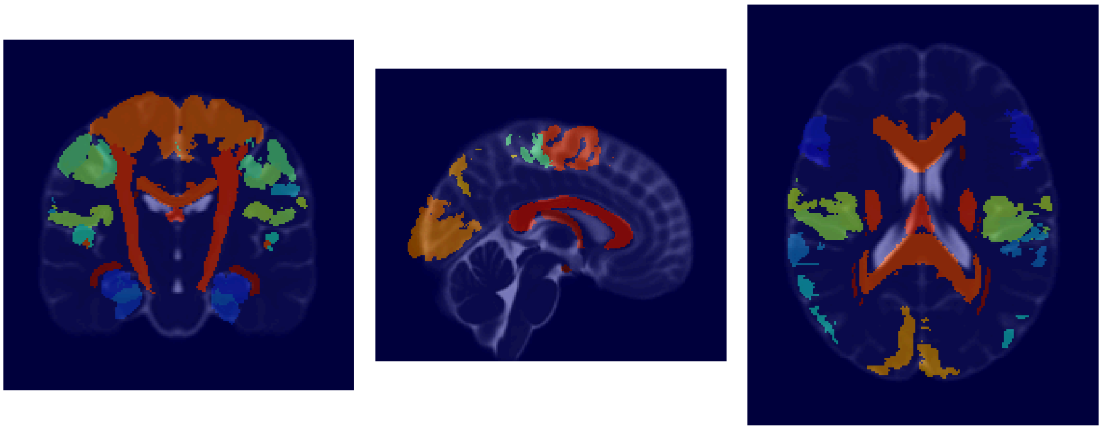

Pauli 2017

Schaefer 2018

Schaefer 2018

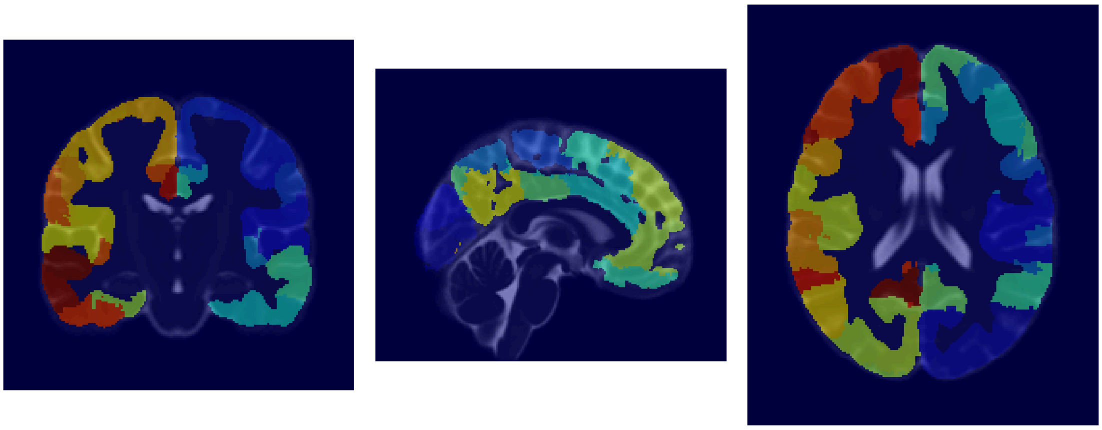

Fiber bundles from the HCP dMRI dataset

Jülich-Brain Cytoarchitectonic Atlas (v1.18)

Julich-Brain Cytoarchitectonic Atlas (v1.18)

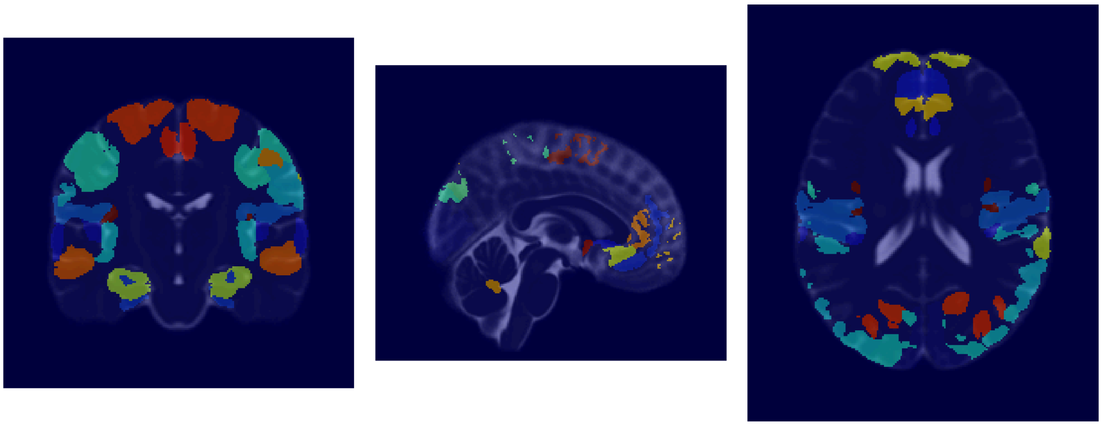

Julich-Brain Cytoarchitectonic Atlas (v2.9)

Julich-Brain Cytoarchitectonic Atlas (v3.0.3)

Julich-Brain Cytoarchitectonic Atlas (v3.1)

MarsAtlas cortical parcellation model

Von Economo-Koskinas Brain Atlas

Desikan-Killiany Atlas (2006)

Desikan-Killiany Atlas (2006)

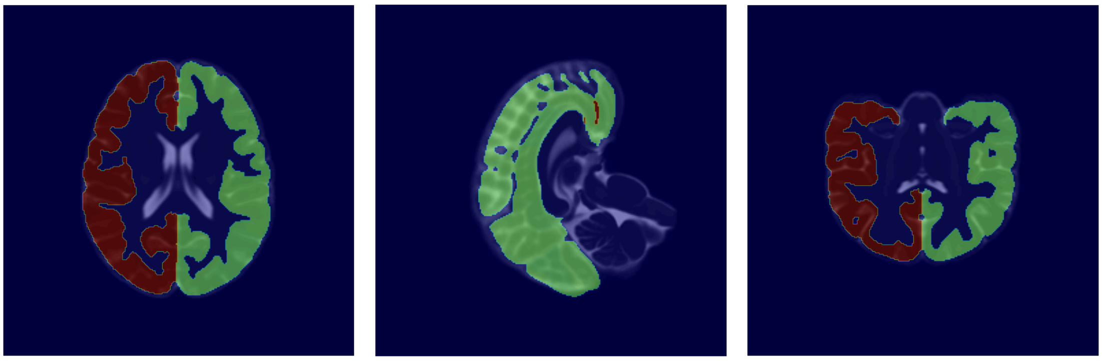

Supplement: Supplementary file 1 [file brainsci-16-01123-s001.zip › Supplementary file S1.pdf]
