# Supplementary material for: Tractometric: A Diffusion Tractography Software Engine for Integration into a Neuronavigation System: Technical Development and Preliminary Clinical Feasibility
Source: Brain Sci. 2026 Oct 17;16(10):1123. doi: 10.3390/brainsci16101123 (PMC13702410; doi:10.3390/brainsci16101123)
Supplement: Supplementary file 1 [file brainsci-16-01123-s001.zip › Supplementary file S2.pdf]

Supplementary file S2. Diffusion MRI acquisition and processing report: series-level description

Every value below was measured directly from the DICOM headers of the studies themselves, except the scanner hardware table, which is labelled as vendor-published documentation. Nothing is inferred: a field the scanner did not record is named as such rather than filled in.

Study design

- **Technical series: 30 patients**, one diffusion acquisition each. Patients were identified by DICOM Patient anonymized number rather than by archive folder, because several studies were stored under more than one spelling of the same name.
- **Clinical feasibility subset: 8 patients**, the most recent studies acquired on the most-used scanner (Aera, syngo MR E11), acquired between 2021-07-19 and 2024-10-12.

The two are reported separately because they answer different questions. The technical series shows that the engine runs on whatever a retrospective, multi-center archive contains, and is heterogeneous by construction. The clinical subset is a single protocol on a single machine, and is the only part of the material that can legitimately be described with one set of acquisition values.

1. Technical series (n = 30)

These studies were acquired retrospectively under routine clinical protocols at several institutions, not under a common research protocol.

They cannot be summarized by a single set of acquisition parameters, and the distributions below are the evidence for that statement rather than an estimate of it.

Scanner model

Model	Patients
Aera	15
MAGNETOM Sola Fit	5
SIGNA Explorer	3
SIGNA Prime	2
Avanto_DOT	2
Achieva	1
Optima MR360	1
Signa HDxt	1

Shells (non-zero b, s/mm2)

Shells	Patients
1000	28
not recorded	1
700	1

Unique diffusion directions

**Directions**	**Patients**
64	20
25	3
40	2
20	2
not recorded	1
15	1
24	1

b0 volumes

**b0**	**Patients**
3	14
1	6
12	5
16	2
Not recorded	1
10	1
2	1

Parameter ranges across the technical series

**Parameter**	**Range**

TR (ms)	2800 - 12249
TE (ms)	84 - 111.6
Slice thickness (mm)	3 - 5
Interslice spacing (mm)	3 - 6.5
Pixel bandwidth (Hz)	1235 - 1953

2. **Clinical subset (n = 8): a single protocol**

All 8 studies were acquired on the same Aera running syngo MR E11, between 2021-07-19 and 2024-10-12.
A parameter is *uniform* when all eight studies agree on the value.

**Parameter**	**Uniform**	**Value across the 8 studies**
Manufacturer and model	yes	Aera
Scanner software	yes	syngo MR E11
Field strength (T)	yes	1.5
TR (ms)	yes	2800
TE (ms)	yes	86
Flip angle (deg)	yes	90
Slice thickness (mm)	yes	5
Interslice spacing (mm)	yes	6.5
In-plane resolution (mm)	no	1.3068x1.3068 (6);<br>1.3636x1.3636 (1);<br>1.4205x1.4205 (1)

Acquisition matrix	yes	[176, 0, 0, 176]
Pixel bandwidth (Hz)	yes	1235
Echo train length	yes	65
In-plane acceleration (PAT/GRAPPA)	yes	2
Multiband / SMS factor	yes	2
Shells (s/mm2)	yes	1000
Unique directions	yes	64
b0 volumes	no	3 (7); 2 (1)
Volumes per series	no	131 (5); 96 (1); 128 (1); 130 (1)
Slices per volume	no	20 (5); 24 (2); 22 (1)
Phase-encoding axis	yes	anterior-posterior (AP)
Phase polarity	yes	positive (CSA)
Effective echo spacing (ms)	no	0.455 (6); 0.445 (2)
Total readout time (ms)	no	79.622 (6); 77.876 (2)

Acquisition paragraph for the clinical subset

Diffusion-weighted images were acquired with an Aera at 1.5 T (scanner software syngo MR E11) using a single-shot spin-echo EPI diffusion sequence (TR 2800 ms, TE 86 ms, flip angle 90 deg, pixel bandwidth 1235 Hz, acquisition matrix 176x176, in-plane acceleration factor 2, multiband/SMS factor 2). Slice thickness was 5 mm with 6.5 mm interslice spacing and 1.3068-1.4205 (isotropic in-plane) mm in-plane resolution, 20 - 24 slices per volume. The diffusion scheme used b = 1000 s/mm2 along 64 non-collinear directions with 2 - 3 b = 0 volumes (96 - 131 volumes per series). Phase encoding was along the
anterior-posterior (AP) axis with an effective echo spacing of 0.445 -
0.455 ms and a total readout time of 77.9 - 79.6 ms. No field map or reverse phase-encoding acquisition was available, so no susceptibility distortion correction was applied.

Where a parameter still varies, it is a per-patient adjustment rather than a

protocol change: field of view, number of slices, and the resulting volume count follow head size and the extent of the region to be covered.

The eight studies

Study date	Directions	b0	Volumes	Slices
2021-07-19	64	3	131	24
2021-11-17	64	3	131	24
2023-01-20	64	3	131	20
2023-03-25	64	2	96	20
2023-04-29	64	3	128	20
2024-02-07	64	3	131	20
2024-08-08	64	3	130	22
2024-10-12	64	3	131	20

3. Scanner hardware (vendor-published documentation)

The headers do not record gradient performance or bore, and gradient strength bounds the achievable b-value, minimum TE, and echo spacing. These values come from vendor documentation and are marked as such so they are never confused with measured data.

Property	Value
Scanner	Siemens MAGNETOM Aera
Field strength	1.5 T
Bore	70 cm

Gradient system	XJ 33 mT/m at 125 T/m/s (XQ option: 45 mT/m at 200 T/m/s)
Software line	syngo MR E11 (shared with MAGNETOM Skyra)
Source	Siemens Healthineers MAGNETOM Aera product documentation; syngo MR E11 operator manual

4. Processing pipeline and tracking parameters

The order in which the software applies each step. Steps marked optional are triggered by the operator and were not applied uniformly across cases.

#	Step	Library performing it
1	DICOM to NIfTI conversion *(optional)*	dicom2nifti.convert_directory
2	Gradient table	dipy.io.read_bvals_bvecs + dipy.core.gradients.gradient_table
3	Motion correction *(optional)*	dipy.align.motion_correction
4	Denoising *(optional)*	dipy.denoise.localpca + pca_noise_estimate
5	Isotropic resampling *(optional)*	dipy.align.reslice.reslice
6	Brain mask	dipy.segment.mask.median_otsu
7	Tissue response	dipy.reconst.csdeconv.auto_response_ssst

8	Diffusion model fit	dipy.reconst.dti / csa / shore / gqi / sfm
9	ODF peaks	dipy.direction.peaks_from_model
10	Stopping criterion	dipy.tracking.stopping_criterion.ThresholdStoppingCriterio
11	Deterministic tracking	src.model.custom_local_tracking.CustomLocalTracking (over dipy.tracking)
12	Probabilistic tracking	dipy.direction.ProbabilisticDirectionGetter.from_pmf
13	Streamline cleaning	own vectorized implementation
14	Shape-based pruning	dipy.segment.clustering.QuickBundles
15	Bundle metrics	dipy.segment.metric.mdf + own length and curvature computation

Default reconstruction and tracking parameters

The values the controls start with, used unless the operator changed them during a session.

Parameter	Default
Reconstruction model	'DTI'
Spherical harmonic order	6
Relative peak threshold	0.7
Minimum separation angle (deg)	60

Seed density (per voxel, per axis)	3
Step size (mm)	STEP_SIZE_MM (STEP_SIZE_MM = 0.5)
GFA stopping threshold	0.02
Minimum streamline length (points)	int (round(MIN_LENGTH_MM / STEP_SIZE_MM)) (MIN_LENGTH_MM = 30.0, STEP_SIZE_MM = 0.5)
Maximum streamline length (points)	600
Probabilistic maximum angle (deg)	35

Technical Limitations

1. No susceptibility distortion correction. Susceptibility distortion correction is not implemented in the software, and this is a real flaw rather than a formality. The distortion displaces signal along the phase-encoding axis, which was anterior-posterior in 39 of the 40 series. From the readout times recovered from vendor private tags (79.6 ms on the scanner used for the clinical subset), an off-resonance of 100 Hz, typical near the frontal sinuses and the petrous bone at 1.5 T, displaces tissue by about 8 voxels, close to 1 cm. Those regions are frontal and temporal, which is exactly where the language pathways reported here run, and because the error is geometric it is carried into the navigation space together with the tractogram.

The standard correction estimates the underlying field from a dedicated field map or from a pair of acquisitions with opposite phase-encoding polarity. Neither is part of the current workflow, so no such estimate is available to the pipeline, and the distortion is left uncorrected.

Future work. The data this correction needs is already acquired. A preoperative anatomical study is part of the routine neurosurgical protocol, and the software already registers those volumes into patient space with their own registration quality metrics, so an undistorted anatomical reference is already available, in the right space, for the cases that would be corrected. Extending that existing registration from a rigid transform to a nonlinear warp constrained to the phase-encoding axis is therefore the natural route to a correction, with no additional sequence and no change to the acquisition protocol. It requires validation before it

can be trusted, since a wrongly applied correction is worse than the limitation. For prospective studies, adding a reverse phase-encoded b = 0, about 20 seconds of scan time, would additionally enable the standard approach.
Susceptibility-induced EPI distortion was not corrected. Given the readout times used here, this may displace tissue by up to approximately 1 cm along the anterior-posterior axis in frontal and temporal regions, limiting geometric accuracy where the language pathways run, and it is an explicit limitation of the series. Correction against the co-registered preoperative anatomical volume, which the current acquisition protocol already provides, is planned.

2. Lack of b-vector reorientation after motion correction.
The motion correction step also adjusts for b-vector rotation. Head-motion correction was performed with DIPY. Each diffusion-weighted volume was registered to a b = 0 reference using a staged registration pipeline (center-of-mass alignment, translation, rigid-body, and a full 12-degree-of-freedom affine transform), optimized on mutual information over a three-level multi-resolution scheme (smoothing sigmas 3, 1 and 0 voxels; subsampling factors 4, 2 and 1; a maximum of 10,000, 1,000 and 100 iterations per level). Where more than one b = 0 volume was acquired, the b = 0 volumes were first co-registered to one another and averaged to form the reference.
Diffusion gradient directions were reoriented so that they remained consistent with the corrected data. For each volume, the rotational component of its registration transform was isolated by polar decomposition (singular-value decomposition, discarding the scale and shear terms), applied to the corresponding b-vector, and the result renormalized to unit length; b = 0 volumes carry no orientation and were left unchanged. The reoriented gradient table was already used for all subsequent model fitting and tractography. The same registration transforms were reused to derive per-subject motion estimates (mean and maximum translation and rotation relative to the reference, and framewise displacement between consecutive volumes), which are reported as quality-control measures.
The rotated vectors are written back over the .bvec file, because saving app state already overwrites the NIfTI with the corrected data, and leaving the original vectors beside it would pair corrected data with unrotated gradients on the next load.

3. No dedicated eddy-current correction. There is no dedicated step, and there will not be one: no pure-Python equivalent of FSL eddy exists, and FSL cannot ship inside a signed Windows installer. Reimplementing its Gaussian-process outlier model is research-grade work that would not survive validation in the time available.
Motion correction runs a 12-DOF affine per volume, whose shear and scale terms

along the phase-encoding axis absorb the first-order (linear) eddy-current distortion. That is a partial correction.
No dedicated eddy-current correction (for example FSL eddy) was applied. The 12-degree-of-freedom affine registration used for motion correction step includes scaling and shear terms, which absorb the first-order, linear component of eddy-current-induced distortion along the phase-encoding direction. Higher-order, non-linear eddy-current effects were not modelled, and no slice-wise outlier detection or signal replacement was performed.
Limitations. Because only the first-order component of eddy-current distortion is absorbed by the affine registration, residual eddy-current distortion may persist, and this should be taken into account when interpreting fibre orientations, particularly towards the edges of the field of view. The magnitude of the effect is bounded by the acquisition: the series were single-shell at b = 1000 s/mm2, where eddy-current distortion is expected to be smaller than at the higher b-values used in multi-shell protocols, since eddy currents scale with the amplitude and duration of the diffusion-encoding gradients. It is reduced, however, not eliminated.

4. No automated quality control. Quality control was clinical and quantitative, not automated. A neurosurgeon reviewed the reconstructed streamlines and the processing of each case before it was used for planning, and the software reports DIPY's own metrics alongside: per-tract length, curvature and coherence, and mutual information, normalised mutual information, cross-correlation, RMSE and Dice for every registration. Per-subject motion is now derived from the registration transforms as well. Those metrics are comparative rather than absolute, so they inform the review instead of gating it: nothing is excluded automatically.
Quality control combined expert review with quantitative metrics. Every reconstructed tractogram, and the processing applied to produce it, was reviewed by a neurosurgeon before the case was used for surgical planning. Streamline metrics (length, curvature and coherence), registration quality metrics (mutual information, normalised mutual information, cross-correlation, RMSE and Dice) and per-subject motion derived from the registration transforms were computed for each case. No volumes or subjects were excluded automatically.
Limitations. Quality control was descriptive rather than threshold-based: no predefined criteria for volume rejection were applied, and no reproducibility estimates were calculated. Standardised criteria form part of the prospective validation proposed.

5. Preprocessing was operator-driven rather than a fixed pipeline.
Denoising, motion correction and isotropic resampling are independent optional steps, not a fixed pipeline: studies differ in SNR, geometry and motion, and not every case needs every step. What it requires is that the choice be reported per

case.
The application shows the recommended order in each button's tooltip and warns, with the reason, when a step is requested out of order. The warning can be dismissed, so the order stays advisory; what it removes is the silent case.
Recommended order

1. denoising -> 2. motion correction -> 3. isotropic resampling
   ● Denoising first. Local PCA assumes spatially independent noise; any interpolation, motion correction included, correlates it and breaks that assumption.
   ● Resampling last. Each geometric step interpolates and the blur compounds. Resampling first would not help the registration either: interpolating 5 mm slices with a 1.5 mm gap adds no information.

Denoising, head-motion correction and isotropic resampling were available as independent, optional steps and were applied at the operator's discretion according to the characteristics of each study. The recommended order (denoising, motion correction, isotropic resampling) is presented within the application, which warns when a step is requested out of that order; it is advisory and is not enforced.

6. Anisotropic voxels with a slice gap. In the clinical subset, the slice thickness is 5 mm with 6.5 mm interslice spacing, a 1.5 mm gap. Through-plane information that was never acquired cannot be recovered by interpolation or resampling, and this bounds the anatomical accuracy of any reconstructed tract.

7. Single-shell acquisitions at b = 1000 s/mm2. Models of crossing fibres fitted to a single shell are limited by the data rather than by the software.

8. The technical series is retrospective and multi-center. Its heterogeneity is a property of the material, not a design choice, and no conclusion about acquisition-dependent performance can be drawn from it.
