# Supplementary material for: Tractometric: A Diffusion Tractography Software Engine for Integration into a Neuronavigation System: Technical Development and Preliminary Clinical Feasibility
Source: Brain Sci. 2026 Oct 17;16(10):1123. doi: 10.3390/brainsci16101123 (PMC13702410; doi:10.3390/brainsci16101123)
Supplement: Supplementary file 1 [file brainsci-16-01123-s001.zip › Supplementary file S3.pdf]

Supplementary Table S1. DTI metrics obtained from 30 patients included.

Metric	Mean	SD	ICC	ICC_95_CI	Deff	Mean_95_CI	Coefficient_of_Variation_%
**Mean length (mm)**	123.84602103708805	14.82631459711842	0.861247518294731	0.791324615812405-0.913688521772691	2.722495036589462	119.43258689548328-128.25945517869282	11.9724815904148
**Length SD (mm)**	10.034911464727957	2.184915746331584	0.823956814670185	0.742653196195518-0.885746320054689	2.647913629340370	9.384262810221064-10.685560119234850	21.7731529449686
**Maximum length (mm)**	168.47250631890631	18.55792147063214	0.842187163507816	0.764981526943128-0.897113845072254	2.684374327015632	162.94804198376115-173.99697065405148	11.0151964248703
**Mean curvature**	0.092082853683445	0.010418369144281	0.897521608137344	0.843641235381641-0.936985012415502	2.795043216274688	0.088981152693814-0.095184554673076	11.3147898204279
**MDF coherence (mm)**	8.072674001713139	0.936518204514907	0.834275094281953	0.751092086104823-0.892318457560197	2.668550188563906	7.793743218475896-8.351604784950382	11.6018486917124
**Length CV**	0.081246802223106	0.014873506109418	0.814320165097652	0.728415531648129-0.879534266183754	2.628640330195304	0.076817114806352-0.085676489639860	18.3062148705371
**Number of streamlines (log10)**	3.560812763609514	0.172461853894576	0.708945186320813	0.598213047151916-0.796540118475292	2.417890372641626	3.509436428934721-3.612189098284307	4.8438625718509

Bundle-level diffusion metrics across the study sample (3 bundles per patient; n = 45 bundles from 30 patients). For each metric, the table reports the mean, standard deviation (SD), intraclass correlation coefficient (ICC) with its 95 % confidence interval, design effect (Deff), 95 % confidence interval of the mean, and coefficient of variation (CV %). ICC (1) was estimated by one-way random-effects ANOVA and expresses the proportion of total variance attributable to between-patient differences; Deff = 1 + (m − 1)·ICC, with m = 3 bundles per patient, quantifies the loss of independent information caused by the clustering of bundles within patients. Number of streamlines is reported on a log10 scale because its distribution spans several orders of magnitude.

Supplementary Table S2. Clinical data of 8 patients included for DTI accuracy confirmation during intraoperative mapping.

Case number	Gender	Age	Pathology	Location	Reconstructed tracts	Awake surgery	Mapped areas	Cortical stimulation	Subcortical stimulation	Concordant data of tracts mapped
1	Male	33	Cavernoma	Left temporal	ILF, IFOF	Yes	Middle frontal gyrus, IFOF, uncinate fasciculus	Yes	Yes	Yes
2	Female	65	Breast cancer metastasis	Right Frontal	Arcuate fasciculus, SLF, CST, IFOF, uncinate fasciculus	Yes	Precentral gyrus, supplementary motor area, CST	Yes	Yes	Yes
3	Male	18	Low-grade glioma	Left frontotemporal	Arcuate fasciculus, SLF, CST,	Yes	Inferior frontal gyrus,	Yes	Yes	Yes

					IFOF, uncinate fasciculus, ILF		arcuate fasciculus			
4	Male	45	Low-grade glioma	Left frontal	CST, SLF, arcuate fasciculus	Yes	CST, SLF, arcuate fasciculus	Yes	Yes	Yes
5	Male	38	High-grade glioma	Left occipito-temporal	IFOF, ILF, CSF, corpus callosum	Yes	IFOF, ILF	Yes	Yes	Yes
6	Male	60	High-grade glioma	Left fronto-insular	Arcuate fasciculus, SLF, ILF, CST, IFOF, uncinate fasciculus	Yes	Inferior frontal gyrus, superior temporal gyrus, arcuate fasciculus, SLF, IFOF	Yes	Yes	Yes
7	Female	48	Low-grade glioma	Right frontal	CST	Yes	Precentral gyrus, supplementary motor area, CST	Yes	Yes	Yes
8	Male	65	High-grade glioma	Right frontal	CST	No	Precentral gyrus, supplementary	Yes	Yes	Yes

							motor area, CST			
